# Supplementary figures and images for: Hydroxychloroquine for the treatment of severe respiratory infection by COVID-19: A randomized controlled trial
Source: PLoS One. 2021 Sep 28;16(9):e0257238. doi: 10.1371/journal.pone.0257238 (PMC8478184; doi:10.1371/journal.pone.0257238)

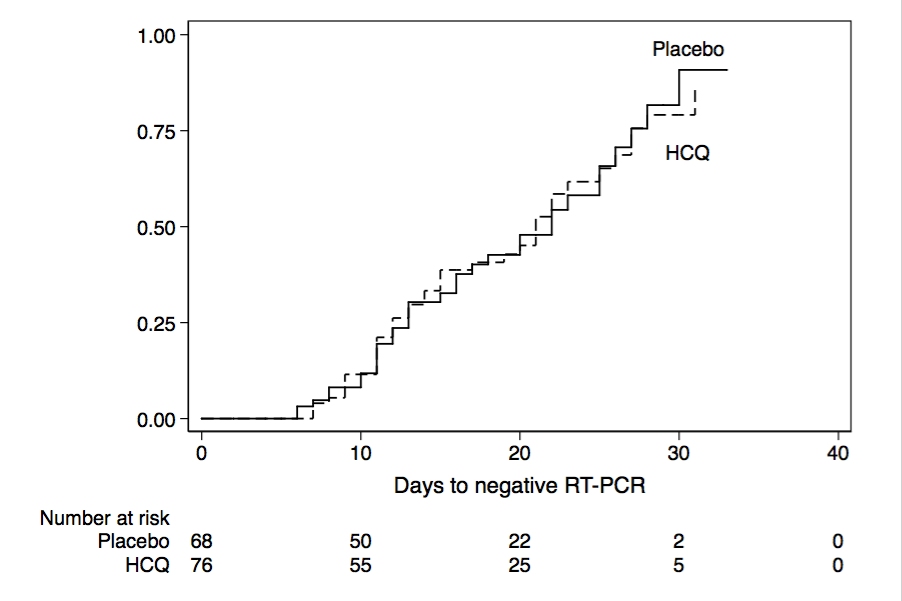

Supplement: S1 Fig — Time to a negative RT-PCR test during hospitalization in hydroxychloroquine (HCQ) and placebo groups, with overlapping curves and no significant difference between them. In the table, the number of individuals at risk as a function of time. Sixty nine of 214 participants only had the initial test, especially in Oaxaca, as the hospital sends the samples to a state laboratory and did not accept additional samples from the same patient. The number of tests done during hospitalization was proportional to duration of hospitalization. (TIF) [file pone.0257238.s001.tif]
