# Supplementary material for: Hydroxychloroquine for the treatment of severe respiratory infection by COVID-19: A randomized controlled trial
Source: PLoS One. 2021 Sep 28;16(9):e0257238. doi: 10.1371/journal.pone.0257238 (PMC8478184; doi:10.1371/journal.pone.0257238)
Supplement: S1 Table — (DOCX) [file pone.0257238.s002.docx]

**SUPPLEMENTARY INFORMATION**

**TABLE S1.** Key measurements at randomization.

| Variable | Treatment assigned | | Respiratory support at randomization | |
| --- | --- | --- | --- | --- |
|  | Placebo n=108(%) | Hydroxychloroquine  n=106(%) | No mechanical ventilation*  n=52(%) | Mechanical ventilation  n=162(%) |
| **On arrival to emergency room** | | | | |
| SpO_2_ (%) | 66 (20) | 63 (20) | 79(15) | 61(19) |
| Pulse rate (min^-1^) | 105 (18) | 111 (16) | 105(13) | 109(18) |
| Breathing frequency (min^-1^) | 32 (11) | 33 (10) | 27(9) | 34(10) |
| **On randomization** | | | | |
| PaO_2_ (mmHg) | 69(22) | 70(11) | 71(31) | 69(18) |
| SaO_2_ (%) | 88.2(12.7) | 90.9(9) | 90(10) | 90(11) |
| FiO_2_ (%) | 54(22) | 56(20) | 39(16) | 60(20) |
| PaO_2_/FIO_2_ (mmHg) | 148 (75) | 141 (59) | 207 (81) | 130 (54) |
| PaCO_2_ (mmHg) | 43(15) | 44(12) | 35(11) | 46(13) |
| pH | 7.37 (0.09) | 7.37(0.10) | 7.43(0.06) | 7.36(0.1) |
| Breathing frequency (min-^1^) | 25 (5) | 26 (4) | 24(6) | 26(4) |
| Heart rate (min^-1^) | 84 (19) | 84(19) | 85(18) | 83(20) |
| Systolic Blood pressure (mmHg) | 116(16) | 116(17) | 116(15) | 116(17) |
| Diastolic Blood pressure(mmHg) | 70(12) | 70(10) | 72(10) | 69(11) |
| Creatinine (mg/dL) | 1.2(1) | 1.3(1) | 0.83(0.3) | 1.4(1.1) |
| Lymphocytes (10^3^/mm^3^) | 0.95(0.73) | 0.91(0.91) | 1.15(1) | 0.86(0.72) |
| Lactate ** (mmol/L) | 2.2(1.5) | 2.1(1.7) | 1.4(0.7) | 2.4(1.7) |
| Glucose (mg/dL) | 152 (69) | 167(71) | 132 (44) | 167(73) |
| C-RP** (mg/dL) | 20(24) | 19(13) | 18(34) | 10(14) |
| Ferritin**(ng/dL) | 1412(1483) | 1159(1210) | 1444(1747) | 1243(1208) |
| D-dimer** (mcg/mL) | 69(179) | 51(151) | 59(153) | 60(170) |
| BNP **(pg/mL) | 75(115) | 132(273) | 43(52) | 127(243) |
| Procalcitonin**(ng/mL) | 8.3(49) | 7(34) | 0.58(1.3) | 9.7(48) |
| SOFA score (points) | 5(3) | 5(2) | 3(2) | 6(2) |

Continuous variables are expressed in mean and standard deviation; categorical variables are expressed as percentage. P was obtained by the student T test for continuous variables and chi2 for categorical variables.

SpO_2_: oxygen saturation by pulse oximeter; PaO_2_: partial pressure of oxygen in arterial blood; SaO_2_: arterial oxygen saturation ; FiO_2_: fraction of inspired oxygen ; PaO_2_/FiO_2_: ratio of arterial partial pressure of oxygen and fraction of inspired oxygen; PaCO_2_: partial pressure of carbon dioxide in arterial blood; C-RP: c-reactive protein; BNP: brain natriuretic peptide: SOFA: Sequential Organ Failure Assessment; HCQ: hydroxychloroquine

*Include patients with low-flow and high-flow oxygen therapy by nasal prongs.

**Values obtained in the first 3 days of the randomization
