# Supplementary material for: Hydroxychloroquine for the treatment of severe respiratory infection by COVID-19: A randomized controlled trial
Source: PLoS One. 2021 Sep 28;16(9):e0257238. doi: 10.1371/journal.pone.0257238 (PMC8478184; doi:10.1371/journal.pone.0257238)
Supplement: S2 Table — Isolated microorganisms in different clinical samples. (DOCX) [file pone.0257238.s003.docx]

**SUPPLEMENTARY INFORMATION**

**TABLE S2.** Isolated microorganisms in different clinical samples.

| Bacteria | Respiratory tract | Blood | Urine |
| --- | --- | --- | --- |
| *Pseudomonas aeruginosa* | 8(42%) | 1(7%) | 13(25%) |
| *Klebsiella pneumonie* | 8(42%) | 1(7%) | 6(12%) |
| *Escherichia coli* | 4(21%) | 1(7%) | 9(18%) |
| *Acinetobacter baumanni* | 2(10%) | 1(7%) | 9(18%) |
| *Staphylococcus aureus* | 1(5%) | 3(20%) | 7(14%) |
| *Candida* sp | 1(5%) | 0 | 7(14%) |
| *Streptoccoccus pneumoniae* | 1(5%) | 0 | 0 |
| *Enterobacter* | 0 | 1(7%) | 4(8%) |

*Except for Acinetobacter (more common in placebo), no differences were found between placebo and HCQ (10 in placebo and 3 in HCQ group, p=0.045)
