# Supplementary material for: Hydroxychloroquine for the treatment of severe respiratory infection by COVID-19: A randomized controlled trial
Source: PLoS One. 2021 Sep 28;16(9):e0257238. doi: 10.1371/journal.pone.0257238 (PMC8478184; doi:10.1371/journal.pone.0257238)
Supplement: S3 Table — (DOCX) [file pone.0257238.s004.docx]

**S3 Table.** Current published randomized controlled trials on HCQ

| **Study** | **Publication status** | **Country** | **Number of patients** | **Type of patients recruited** | **Drug tested** | **HCQ dose** | **Results** |
| --- | --- | --- | --- | --- | --- | --- | --- |
| Tang et al. | Peer reviewed | China | 150 | Overall hospitalized patients | HCQ | LD: 1200mg + 200mg t.i.d; 3w | No benefit in viral clearance |
| Mitjà et al. | Peer reviewed | Spain | 353 | Ambulatory patients | HCQ | 3200mg | No benefit in viral clearance or hospitalization rate |
| Molina et al. | Peer reviewed | France | 11 | Only moderate to severe hospitalized patients | HCQ+AZM | 6000mg | No benefit in viral clearance |
| Abd-Elsalam et al. | Peer reviewed | Egypt | 194 | - | HCQ | - | No difference in mortality or hospital days |
| RECOVERY Collaborative Group | Peer reviewed | United Kingdom | 7513 | - | HCQ | - | No difference in mortality |
| WHO Solidarity Trial Consortium | Peer reviewed | 30 countries | 11330 | Overall hospitalized patients | HCQ + REM + LOP + INF | LD: 800 mg + 400mg b.i.d.;10d | No difference in mortality, MV requirements or hospital stay |
| Self et al. | Peer reviewed | United States | 479 | Overall hospitalized patients | HCQ | LD: 400mg b.i.d. 2 doses + 200mg b.i.d; 5d | No benefit in clinical status at day 14 |
| Horby et al. | Preprint | United Kingdom | 4686 | Overall hospitalized patients | HCQ, HCQ+AZM | LD: 800mg q.6.h 2 doses + 400mg b.i.d; 10d | No difference in mortality |
| Chen et al. | Preprint | China | 62 | Only mild hospitalized patients | HCQ | 2000mg | Shorter symptom remision time in HCQ group |
| Cavalcanti et al. | Preprint | Brazil | 667 | Only mild to moderate hospitalized patients | HCQ, HCQ+AZM | 5600mg | - |
| Lyngbakken et al. | Preprint | Norway | 53 | Overall hospitalized patients | HCQ | 400mg b.i.d; 7d | - |

AZM= Azithromycin; HCQ= Hydroxicloroquine; INF= Interferon B1a; LOP= Lopinavir; REM=Remdesivir.

Reference:

1. Tang W, Cao Z, Han M, et al. Hydroxychloroquine in patients with mainly mild to moderate coronavirus disease 2019: open label, randomised controlled trial. BMJ 2020; 369: m1849.
2. Mitjà O, Corbacho-Monné M, Ubals M, Tebe C, Peñafiel J, Tobias A, et al. Hydroxychloroquine for early treatment of adults with mild COVID-19: a randomized – controlled trial. Clin Infect Dis 2020.
3. Molina JM, Delaugerre C, Le GoffJ, Mela-Lima B, Ponscarme D, Goldwirt L, et al. No evidence of rapid antiviral clearance or clinical benefit with the combination of hydroxychloroquine and azithromycin in patients with severe COVID-19 infection. Med Mal Infect 2020;50: 30085 – 8.
4. Abd-Elsalam S, Esmail E, Khalaf M et al. Hydroxychloroquine in the treatment of COVID-19: A multicenter randomized controlled study. American Journal of Tropical Medicine and Hygiene, (2020), 1635-1639, 103(4)
5. The RECOVERY Collaborative Group. Effect of Hydroxychloroquine in Hospitalized Patients with Covid-19. New England Journal of Medicine, (2020), 2030-2040, 383(21)
6. WHO Solidarity Trial Consortium. Repurposed Antiviral Drugs for Covid-19 - Interim WHO Solidarity Trial Results. New England Journal of Medicine, (2021), 497-511, 384(6)
7. Self W, Semler M, Leither L et al. Effect of Hydroxychloroquine on Clinical Status at 14 Days in Hospitalized Patients with COVID-19: A Randomized Clinical Trial. JAMA - Journal of the American Medical Association, (2020), 2165-2176, 324(21)
8. Horby P, Mafham M, Linsell L, Bell JL, Staplin N, Emberson JR, et al. Effect of hydroxychloroquine in hospitalized patients with COVID-19: preliminary results from a multi-centre, randomized, controlled trial. medRxiv 2020.
9. Chen Z, Hu J, Zhang Z et al. Efficacy of hydroxychloroquine in patients with COVID-19: results of a randomized clinical trial. medRxiv 2020; doi: 10.1101/2020.03.22.20040758.
10. Cavalcanti AB, Zampieri FG, Azevedo LC, Rosa RG, Avezum A, Veiga VC, et al. Hydroxychloroquine alone or in combination with azithromycin to prevent major clinical events in hospitalised patients with coronavirus infection (COVID-19): rationale and design of a randomised, controlled clinical trial. medRxiv 2020.
11. Lyngbakken M.N, Berdal J, Eskesen A et al. A pragmatic randomized controlled trial reports the ecacy of hydroxychloroquine on coronavirus disease 2019 viral kinetics.
