# Supplementary material for: Hydroxychloroquine for the treatment of severe respiratory infection by COVID-19: A randomized controlled trial
Source: PLoS One. 2021 Sep 28;16(9):e0257238. doi: 10.1371/journal.pone.0257238 (PMC8478184; doi:10.1371/journal.pone.0257238)
Supplement: S1 File — (PDF) [file pone.0257238.s005.pdf]

HYDROXYCHLOROQUINE FOR THE TREATMENT OF SEVERE RESPIRATORY INFECTION BY COVID-19: A RANDOMIZED CONTROLLED TRIAL, CONSORT GUIDELINES

Item	Description	Reported on-line number
Title	Identification of the study as randomized	2
Authors *	Contact details for the corresponding author	Front page
Trial design	Description of the trial design (e.g. parallel, cluster, non-inferiority)	Methods
Methods		
Participants	Eligibility criteria for participants and the settings where the data were collected	Methods
Interventions	Interventions intended for each group	Methods
Objective	Specific objective or hypothesis	Introduction
Outcome	Clearly defined primary outcome for this report	Methods
Randomization	How participants were allocated to interventions	Methods
Blinding (masking)	Whether or not participants, care givers, and those assessing the outcomes were blinded to group assignment	Methods
Results		
Numbers randomized	Number of participants randomized to each group	Results, flow chart
Recruitment	Trial status	Results, flow chart
Numbers analyzed	Number of participants analyzed in each group	Flow chart
Outcome	For the primary outcome, a result for each group and the estimated effect size and its precision	Results
Harms	Important adverse events or side effects	Results, Table
Conclusions	General interpretation of the results	Yes
Trial registration	Registration number and name of trial register	Yes
Funding	Source of funding	Acknowledgments

**This item is specific to conference abstracts*
