# Supplementary material for: Hydroxychloroquine for the treatment of severe respiratory infection by COVID-19: A randomized controlled trial
Source: PLoS One. 2021 Sep 28;16(9):e0257238. doi: 10.1371/journal.pone.0257238 (PMC8478184; doi:10.1371/journal.pone.0257238)
Supplement: S2 File — (PDF) [file pone.0257238.s006.pdf]

ClinicalTrials.gov Protocol Registration and Results System (PRS) Receipt
Release Date: March 18, 2020

ClinicalTrials.gov ID: [Not yet assigned]

Study Identification

Unique Protocol ID: HidroxycloroquinaCOVID19

Brief Title: Hydroxycloroquine Treatment for Severe COVID-19 Pulmonary Infection (HYDRA Trial)

Official Title: Hydroxycloroquine Treatment for Severe COVID-19 Respiratory Disease: Randomised Clinical Trial (HYDRA Trial)

Secondary IDs:

Study Status

Record Verification: March 2020

Overall Status: Not yet recruiting

Study Start: March 23, 2020 [Anticipated]

Primary Completion: October 31, 2020 [Anticipated]

Study Completion: March 22, 2021 [Anticipated]

Sponsor/Collaborators

Sponsor: National Institute of Respiratory Diseases, Mexico

Responsible Party: Sponsor

Collaborators: Sanofi

Oversight

U.S. FDA-regulated Drug: No

U.S. FDA-regulated Device: No

U.S. FDA IND/IDE: No

Human Subjects Review: Board Status: Pending
Board Name: Ethics and Bioethics in Research Committee
Board Affiliation: National Institute of Respiratory Diseases
Phone:
Email: ccybiner@yahoo.com.mx
Address:

Calzada de Tlalpan 4502. Col. Sección XVI.
Del. Tlalpan. Mexico City 14080

Data Monitoring: Yes

FDA Regulated Intervention: No

Study Description

Brief Summary: Double blinded randomized clinical trial designed to evaluate the security and efficacy of hydroxycloroquine as treatment for COVID-19 severe respiratory disease. We hypothesize that a 400mg per day dose of hydroxycloroquine for 10 days will reduce all-causehospital mortality in patients with severe respiratory COVID-19 disease.

Detailed Description:

Conditions

Conditions: COVID-19
Severe Acute Respiratory Syndrome Coronavirus 2

Keywords: COVID-19
SARS

Study Design

Study Type: Interventional

Primary Purpose: Treatment

Study Phase: Phase 3

Interventional Study Model: Parallel Assignment
Double blinded, randomized controlled trial

Number of Arms: 2

Masking: Quadruple (Participant, Care Provider, Investigator, Outcomes Assessor)
Clinical practitioners and data analysts will remain blinded all through the study. Blinding will end in case the attending physician considers the patient should abandon the study or some of the exclusion/elimination criteria apply.

Allocation: Randomized

Enrollment: 500 [Anticipated]

Arms and Interventions

Arms	Assigned Interventions
Active Comparator: treatment<br>Hydroxychloroquine tablet 200mg every 12 hours for 10 days.	Drug: Hydroxychloroquine<br>hydroxychloroquine 400mg day for 10 days
Placebo Comparator: placebo<br>identical placebo, one tablet every 12 hours for 10 days	Drug: Placebo oral tablet<br>Placebo oral tablet

Outcome Measures

Primary Outcome Measure:

1. All-cause hospital mortality
   incidence of all-cause mortality

   [Time Frame: Until Hospital Discharge of Death]

Secondary Outcome Measure:

2. Length of hospital stay
   Days from ER admission to hospital discharge

   [Time Frame: Until Hospital Discharge or Death]
3. Need of mechanical ventilation
   need of invasive or non invasive mechanical ventilation

   [Time Frame: Until Hospital Discharge of Death]
4. Ventilator free days
   28 minus days without invasive ventilation support in patients with invasive mechanical ventilation at randomization

   [Time Frame: Until Hospital Discharge of Death]
5. Grade 3-4 adverse reaction
   Adverse Reactions

   [Time Frame: Until Hospital Discharge of Death]

Eligibility

Minimum Age: 18 Years

Maximum Age: 80 Years

Sex: All

Gender Based:

Accepts Healthy Volunteers: No

Criteria: Inclusion Criteria:

1. Signed informed consent
2. negative pregnancy test in women
3. COVID -19 confirmed by rtPCR in any respiratory sample.
4. Severe COVID - 19 disease defined as any from the following:

   a. SpO2 less than 91% or a 3% drop from base SpO2 or need to increase supplementary oxygen in chronic hypoxia
   b. need for mechanical ventilation (invasive or non invasive)
   c. sepsis/septic shock.

Exclusion Criteria:

1. history of anaphylactic shock to hydroxychloroquine.
2. History of previous administration of chloroquine or hydroxychloroquine (within 1 month)
3. decision of attending physician by any reason.
4. History of chronic hepatic disease (Child-Pugh B or C)
5. History of Chronic renal disease (GFR less than 30)

Contacts/Locations

Central Contact Person: Carmen Hernandez-Cárdenas, MD. MSc
Telephone: 54871700 Ext. 5213
Email: cmhcar@hotmail.com

Central Contact Backup: Rogelio Perez-Padilla, MD. PhD.
Telephone: 54871700
Email: perezpad@gmail.com

Study Officials: Carmen Hernandez-Cárdenas, MD. MSc.
Study Principal Investigator
National Institute of Respiratory Diseases - México

Luis-Felipe Jurado-Camacho, MD
Study Director
National Institute of Respiratory Diseases - México

Ireri Thirion-Romero, MD. MSc
Study Chair
National Institute of Respiratory Diseases - México

Sebastian Rodriguez-Llamazares, MD.MPH
Study Chair
National Institute of Respiratory Diseases - México

Rogelio Perez-Padilla, MD. PhD
Study Director
National Institute of Respiratory Diseases - México

Cristobal Guadarrama, MD MSc
Study Chair
National Institute of Respiratory Diseases - México

Joel Vasquez-Pérez, MD
Study Chair
National Institute of Respiratory Diseases - México

Locations:

IPDSharing

Plan to Share IPD: Undecided
As requested by other investigators.

References

Citations:

Links:

Available IPD/Information:

U.S. National Library of Medicine | U.S. National Institutes of Health | U.S. Department of Health & Human Services

DIRECCIÓN GENERAL

OFICIO: HRAEO/DG/267/2020

San Bartolo Coyotepec, Oaxaca; 06 de mayo 2020.

Asunto: Carta de Apoyo Institucional.

Lic. Adriana Espinosa Jove
Jefa del Departamento de Apoyo Técnico
Unidad de Investigación, INER
Presente

En el Hospital Regional de Alta Especialidad de Oaxaca, participamos con convicción y mantenemos el compromiso permanente de realizar Investigación es por eso que manifiesto el interés y Apoyo Institucional para que se lleve a cabo el protocolo denominado "Hidroxicloroquina para el tratamiento de infección respiratoria grave por COVID-19: ensayo clínico controlado. Protocolo Número C13-20", y teniendo como Investigador principal en el Hospital al Dr. Néstor Gabriel Cruz Ruíz, Médico Intensivista.

Sin más por el momento, reciba un cordial saludo.

Atentamente

SALUD HRAEO
HOSPITAL REGIONAL DE ALTA ESPECIALIDAD DE OAXACA.

Dra. Alba Vásquez Palacios
Directora General
Hospital Regional de Alta Especialidad de Oaxaca

Validó

Dra. Liliam Irasema García Pérez
Directora de Planeación, Enseñanza e Investigación
Hospital Regional de Alta Especialidad de Oaxaca
ensenanza@hraeoaxaca.gob.mx
Tel.951515018080 ext.1095

MJPC*

Ixtapaluca, Estado de México a 27 de abril de 2020
Oficio No. DG/HRAEI/0721/2020

DR. JORGE SALAS HERNÁNDEZ
DIRECTOR GENERAL DEL INSTITUTO NACIONAL
DE ENFERMEDADES RESPIRATORIAS
ISMAEL COSÍO VILLEGAS
P R E S E N T E

A efecto de combatir el virus conocido como SARS-CoV-2 (COVID – 19), se ha puesto en marcha un protocolo para el manejo y tratamiento de los pacientes con infección; que se actualiza permanentemente. Este documento complementa este protocolo y actualiza los medicamentos disponibles para la infección.

Algunos de los tratamientos disponibles son moléculas de nuevo desarrollo y otras son usos nuevos de medicamentos ya autorizados en otras indicaciones. De todo ello se genera constantemente una gran cantidad de información que va siendo analizada conforme está disponible. Además, es necesario actualizar e informar de la forma en la que se puede acceder a los medicamentos disponibles.

A este protocolo se le ha denominado "HIDROXICLOROQUINA PARA EL TRATAMIENTO DE INFECCIÓN RESPIRATORIA GRAVE POR COVID-19: ENSAYO CLÍNICO CONTROLADO MULTICÉNTRICO", con código C13-20, con aprobación por medio del Comité Único, así como aprobación por COFEPRIS.

Por lo anterior, manifiesto el deseo del Hospital Regional de Alta Especialidad de Ixtapaluca de adherirse al citado protocolo y la aceptación incondicional de todas sus cláusulas.

Quedando como investigadora responsable del Hospital Regional de Alta Especialidad de Ixtapaluca, Dra. Patricia Alejandra Meza Meneses.

Sin otro particular reciba saludos cordiales.

A T E N T A M E N T E
DIRECTORA GENERAL

DRA. ALMA ROSA SÁNCHEZ CONEJO
HOSPITAL REGIONAL DE ALTA ESPECIALIDAD DE IXTAPALUCA
c.c.p. **Dr. Pérez Padilla José Rogelio**

ANEXO 1

FORMATO DE OFICIO DE SOLICITUD DE ADHESIÓN A LAS BASES

Ixtapaluca, Estado de México a 27 de abril de 2020
Oficio No. DG/HRAEI/0722/2020
Asunto: Solicitud de adhesión a las "Bases de colaboración para la conformación de Comités Únicos".

DR. GUSTAVO REYES TERÁN
TITULAR DE LA COMISIÓN COORDINADORA DE INSTITUTOS NACIONALES DE SALUD Y HOSPITALES DE ALTA ESPECIALIDAD (CCINSHAE).
P R E S E N T E

Me refiero a las *"Bases de colaboración para la conformación de comités únicos (de investigación, ética en investigación y bioseguridad) en los casos de estudios clínicos multicéntricos",* suscritas el diecisiete de septiembre de dos mil catorce, entre los Institutos Nacionales de Salud, Hospitales Federales de Referencia y Hospitales Regionales de Alta Especialidad, cuyo objeto es el reconocimiento mutuo entre los firmantes, respecto de los dictámenes emitidos por el Comité Único que se constituya entre las instituciones colaboradoras y con aval de la CCINSHAE, cuando se trate de estudios multicéntricos en seres humanos y sus partes biológicas, sin necesidad de que los referidos proyectos sean dictaminados nuevamente por el comité de otra institución adherida a "LAS BASES".

Al respecto las unidades hospitalarias, instituciones de salud y centros de investigación que pretendan ser colaboradoras, deberán adherirse a "LAS BASES" solicitándolo por escrito y cumpliendo con los requisitos establecidos en el citado instrumento.

Por lo anterior, manifiesto el deseo del Hospital Regional de Alta Especialidad de Ixtapaluca de adherirse a "LAS BASES" y la aceptación incondicional de todas sus cláusulas.

En razón de lo anterior, solicito de la manera más atenta se sirva tenerme por presentado para suscribir el correspondiente convenio para la adhesión a "LAS BASES", para lo cual anexo copia simple del nombramiento que acredita mi personalidad jurídica.

A T E N T A M E N T E
DIRECTORA GENERAL

DRA. ALMA ROSA SÁNCHEZ CONEJO
HOSPITAL REGIONAL DE ALTA ESPECIALIDAD DE IXTAPALUCA

c.c.p. **Dr. Rodolfo Cano Jiménez.-** Director General de Políticas de Investigación en Salud, CCINSHAE.
c.c.p. **Dr. Francisco Javier Díaz Vásquez.-** Director de Concertación y Difusión Académica, CCINSHAE.

RECOMENDACIONES PARA LLENADO DEL FORMATO

1. Todo proyecto de investigación que sea sometido a evaluación del Comité de Ética en Investigación o al Comité de Investigación, debe ajustarse al formato establecido.

2. Todos los proyectos deben ajustarse a los espacios requeridos, los cuales están diseñados para agilizar la revisión del proyecto.

3. Si algún apartado no aplica para un estudio en particular favor de indicarlo.

4. Es indispensable que siempre se llenen los apartados de consideraciones éticas y de desglose de recursos requeridos para el estudio.

5. En caso de estudios de investigación en humanos, siempre debe anexarse la carta de Consentimiento Informado.

6. Cualquier instrumento de nueva aplicación, como cuestionarios, etc., que se pretendan incluir en el estudio, se deberán anexar a este formato.

7. En caso de presentar a revisión protocolo en colaboración con Industria Farmacéutica es indispensable requisitar la última hoja, *Beneficios para la Institución.*

8. Únicamente se podrá extender en el apartado de *Métodos,* en caso de requerir utilizar más páginas, colocar el cursor por arriba de la línea, oprimir al mismo tiempo la tecla Ctrl y enter.

9. Es indispensable anotar el tipo de riesgo de la investigación, éste deberá ser considerado de acuerdo a lo establecido en el Art. 17 del REGLAMENTO de la Ley General de Salud en Materia de Investigación para la Salud.
   Investigación sin riesgo.
   Investigación con riesgo mínimo.
   Investigación con riesgo mayor que el mínimo.

TITULO DEL PROYECTO
HIDROXICLOROQUINA PARA EL TRATAMIENTO DE INFECCIÓN RESPIRATORIA GRAVE POR COVID-19: ENSAYO CLÍNICO CONTROLADO.

INVESTIGADOR RESPONSABLE Y ADSCRIPCIÓN
Debe contener: nombre, título y adscripción del investigador principal
Dra. Carmen Margarita Hernández Cárdenas
Jefe Áreas Críticas

INVESTIGADORES PARTICIPANTES Y ADSCRIPCIONES
Debe contener: nombres, títulos y adscripciones de los investigadores participantes.
Dr. Cristóbal Guadarrama Adscrito Unidad de Urgencias Respiratorias
Dr. Luis Felipe Jurado Camacho Adscrito Unidad de Cuidados Intensivos Respiratorios
Dr. José Rogelio Pérez Padilla Departamento en Investigación de Tabaquismo y EPOC
Dra. Ireri Isadora Thirión Romero Departamento en Investigación de Tabaquismo y EPOC
Dr. Joel Armando Vasquez Pérez Departamento en Investigación de Tabaquismo y EPOC

FECHA DE INICIO	FECHA DE TÉRMINO
10 – abril 2020	10 abril 2021

CRONOGRAMA

Periodos en: **Meses:** xxxx **Bimestres:** ______ **Otros** ______

	1	2	3	4	5	6	7	8	9	10	11	21	13	14	15	16	17	18	19	20	21	22	23	24
Ejecución	x	x	x	x	x	x	x	x	x	X	x	x	x	x	x	x	x	x	x	x	X			
Análisis																						x	x	X
Preparación de la publicación																							x	x

ÚLTIMAS 5 PUBLICACIONES Y/O PROYECTOS DEL INVESTIGADOR RESPONSABLE

Acute Respiratory Distress Syndrome Secondary to Influenza A(H1N1)pdm09: Clinical Characteristics and Mortality Predictors. Hernández-Cárdenas CM, Serna-Secundino H, García-Olazarán JG, Aguilar-Pérez CL, Rocha-Machado J, Campos-Calderón LF, Lugo-Goytia G. Rev Invest Clin. 2016 Sep-Oct;68(5):235-244. PMID: 27941959 Classifying Acute Respiratory Distress Syndrome Severity: Correcting the Arterial Oxygen Partial Pressure to Fractional Inspired Oxygen at Altitude. Pérez-Padilla R, Hernández-Cárdenas CM, Lugo-Goytia G. Rev Invest Clin. 2016 Jul-Aug;68(4):169-70. PMID: 27623033 Clinical Characteristics and Mortality of Influenza A H1N1 and Influenza-Like Illness in Mexico City in the 2013-2014 Winter Season. Martínez-Briseño D, Torre-Bouscoulet L, Herrera-Zamora Jde J, Díaz-Rico J, Sandoval-Macías G, Pérez-Padilla R, Hernández-Cárdenas C, Regalado-Pineda J, Salas-Hernández J, Santillán-Doherty P. Rev Invest Clin. 2016 May-Jun;68(3):147-53. PMID: 27409002 Influenza A (H1N1pdm09)-Related Critical Illness and Mortality in Mexico and Canada, 2014. Dominguez-Cherit G, De la Torre A, Rishu A, Pinto R, Ñamendys-Silva SA, Camacho-Ortiz A, Silva-Medina MA, Hernández-Cárdenas C, Martínez-Franco M, Quesada-Sánchez A, López-Gallegos GC, Mosqueda-Gómez JL, Rivera-Martinez NE, Campos-Calderón F, Rivero-Sigarroa E, Hernández-Gilsoul T, Espinosa-Pérez L, Macías AE, Lue-Martínez DM, Buelna-Cano C, Ramírez-García Luna AS, Cruz-Ruiz NG, Poblano-Morales M, Molinar-Ramos F, Hernandez-Torre M, León-Gutiérrez MA, Rosaldo-Abundis O, Baltazar-Torres JÁ, Stelfox HT, Light B, Jouvet P, Reynolds S, Hall R, Shindo N, Daneman N, Fowler RA.
Crit Care Med. 2016 Oct;44(10):1861-70. doi: 10.1097/CCM.0000000000001830.
PMID: 2735908 Population Pharmacokinetics of Gentamicin in Mexican Children with severe malnutrition Lares-Asseff I, Pérz-Guillé MG, Camacho Vieyra GA, Pérez AG, Peregrina NB, **Lugo Goytia** G. Pediatr Infect Dis J. 2016 Aug;35(8):872-8. doi: 10.1097/INF.0000000000001204. PMID: 27420805

TITULO DEL PROYECTO

HIDROXICLOROQUINA PARA EL TRATAMIENTO DE INFECCIÓN RESPIRATORIA GRAVE POR COVID-19: ENSAYO CLÍNICO CONTROLADO.

DEFINICIÓN DEL PROBLEMA

El COVID – 19 es un nuevo tipo de neumonía viral descrito en diciembre 2019 en Wuhan – China; representa un problema de salud público importante por su rápida propagación y la capacidad de generar neumonía grave en pacientes susceptibles; al momento no se a descrito ningún tratamiento específico para el virus siendo su manejo principalmente el soporte.

PREGUNTA DE INVESTIGACIÓN

¿El tratamiento por 10 días con 400 mg/día de hidroxicloroquina comparada con placebo reduce la mortalidad intrahospitalaria en sujetos con enfermedad grave por COVID - 19?

ANTECEDENTES

Se deberá proveer información de base para poder entender la relevancia del presente estudio y el propósito y fundamento del estudio.

Se deberá incluir un resumen de los datos de estudios no clínicos que tengan potencialmente una importancia clínica y de estudios clínicos y epidemiológicos relevantes para el presente estudio, así como las referencias de la literatura relevantes al estudio y que provean un sustento para el estudio.

El brote de infección respiratoria por el coronavirus 2019 (2019-nCOV) inició en diciembre del 2019, en la ciudad de Wuhan, provincia de Hubei (China) [1-5]. De esta ciudad, el brote se ha ido extendiendo a varias provincias de China y a otros países [6]. Hasta el 16 de Febrero del 2020, se habían reconocido 28 países con casos confirmados, con 70,548 infecciones y 1770 muertes en China, y 413 infecciones en Japón [6]. Como en otros coronavirus, el del SARS, y el del MERS, la infección original humana probablemente se obtuvo de contacto con animales, pero se ha demostrado una eficaz transmisión entre humanos, con una tasa de crecimiento mayor a 1, estimada de 2.6 [7], y una duplicación rápida de casos que aseguran no solo el mantenimiento de la epidemia si no su expansión.

Hasta el momento actual no se cuenta con un tratamiento efectivo disponible para el COVID-19, sin embargo in vitro, se reporto recientemente la efectividad in vitro del Remdesivir, y de la cloroquina, esta ultima en concentraciones capaces de ser obtenidas in vivo en pacientes que la usan [8]. La cloroquina e hidroxicloroquina, son medicamentos originalmente antimaláricos, en uso regular por décadas y con una toxicidad limitada, a un costo muy bajo, y en caso de ser efectivo in vivo, podría ser un medicamento de gran utilidad. El efecto antiviral amplio, se ha atribuido al incremento del pH de los endosomas, que se requieren para la fusión del virus con la célula y también por interferencia con la glicosilación de los receptores celulares del SARS-COV[9-11]
Adicionalmente al efecto antiviral, se le han atribuido efectos inmunomoduladores que podrían potenciar el efecto antiviral y el impacto sistémico de las infecciones graves.
La gama de presentaciones clínicas del 2019-CoV, es amplia, desde infecciones asintomáticas hasta infecciones fatales, con daño pulmonar grave, falla respiratoria, y falla multiorgánica. El daño grave se ha asociado, como en la influenza grave, a una reacción inflamatoria sistémica o una tormenta de citocinas, que podría ser atenuada por el efecto inmunomodulador de la cloroquina o hidroxicloroquina.

La tasa de letalidad general de la enfermedad por COVID-19 se ha estimado alrededor del 2%, pero en los casos graves en falla respiratoria, o requiriendo cuidados críticos, es considerablemente mayor, entre 10-15%, pero fácilmente podría incrementarse hasta el 20-30% dependiendo de los criterios de entrada a una unidad de terapia intensiva.
Recientemente se publicó una carta [12] que describe que en Febrero 17, el Consejo Estatal de China dio un aviso de prensa indicando que la Cloroquina habia demostrado marcada eficacia y seguridad aceptable para el tratamiento de la neumonía por COVID-19 en ensayos multicéntricos de China con más de 100 pacientes de 10 hospitales.

JUSTIFICACIÓN

Se carece de un tratamiento farmacológico aprobado para el COVID - 19, y basados en la efectividad in Vitro de la cloroquina, su seguridad demostrada por décadas, su costo bajo, se propone un ECC, doble ciego, para evaluar la eficacia y seguridad del compuesto derivado hidroxicloroquina en sujetos hospitalizados con enfermedad respiratoria grave por COVID - 19.

HIPÓTESIS

El tratamiento con Hidroxicloroquina 400 mg/día por 10 días reduce la mortalidad intrahospitalaria comparada con placebo en sujetos con enfermedad respiratoria grave por COVID-19.

OBJETIVO (S) GENERAL (ES)

Estimar si el tratamiento por 10 días con 400 mg/día de hidroxicloroquina reduce la mortalidad intrahospitalaria comparada con placebo en sujetos con enfermedad grave por COVID – 19.

OBJETIVOS ESPECÍFICOS

A) Días de hospitalización
B) Necesidad de apoyo ventilatorio mecánico
C) Días de soporte ventilatorio mecánico.
D) Incidencia de efectos adversos grado 3 y 4.

DISEÑO EXPERIMENTAL

1. INVESTIGACIÓN CLÍNICA x
2. INVESTIGACIÓN BÁSICA
3. INVESTIGACIÓN SOCIAL
4. INVESTIGACIÓN EPIDEMIOLÓGICA

FAVOR DE INDICAR EL TIPO DE ESTUDIO

OBSERVACIONAL
X EXPERIMENTAL
RETROSPECTIVO
X LONGITUDINAL
X PROSPECTIVO
TRANSVERSAL
OTRO FAVOR DE INDICAR:

"Indicar lo que se pretende obtener para el conocimiento en el tema de estudio al desarrollar esta investigación"

1. Comprensión
2. Prevención
3. Diagnóstico
4. Tratamiento X
5. Rehabilitación

Indicar la línea de investigación de la que se deriva este proyecto:

1. Programa de Estudio sobre la Influenza y otros Virus Respiratorios. x
2. Programa de Estudio sobre Problemas Respiratorios asociados a la exposición al Tabaco y Humo de Leña.
3. Programa de Estudio sobre Tuberculosis.
4. Programa de Estudio sobre Enfermedades Fibrosantes del Pulmón.
5. Programa de Estudio sobre Cáncer Pulmonar.
6. Programa de Estudio sobre Asma.
7. Programa de Estudio de la Clínica del Sueño.
8. Programa de Estudio sobre VIH y SIDA.
9. Programa de Estudio en Cirugía y Trasplante Pulmonar Experimental.
10. Programa de Estudio de Investigación en Inmunogenética y Alergia.
11. Programa de Estudio sobre Infecciones Respiratorias Agudas.
12. Programa de Estudio sobre Enfermedades del Oído y vías Respiratorias Altas.
13. Programa de Estudio sobre Enfermedades Reumáticas.
14. Programa de Estudio sobre Enfermedades Metabólicas asociadas a Enfermedades Respiratorias.
15. Programa de Estudio sobre Fisiología Respiratoria.
16. Programa de Estudio sobre Tromboembolia Pulmonar.
17. Programa de Estudio sobre Enfermedades Cardiovasculares y Respiratorias.
18. Programa de Investigación sobre Salud Respiratoria.
19. Programa de Estudio sobre Farmacología.
20. Programa de Desarrollo Biotecnológico.
21. Otro
    Especificar:

MATERIAL Y MÉTODOS

UTILIZAR LAS HOJAS NECESARIAS

A) Lugar del estudio
B) Descripción de la población de estudio
C) Procedimientos del estudio
D) Número necesario de sujetos de investigación
E) Criterios de inclusión y exclusión
F) Captura, procesamiento, análisis e interpretación de la información.

A) Áreas críticas y Hospitalización del Instituto Nacional de Enfermedades Respiratorias Ismael Cosío Villegas

B) Mayores de 18 años con diagnóstico confirmado de COVID – 19 por RT-PCR por muestra faríngea, naso-faríngea o en aspirado traqueal/lavado bronquioalveolar, con diagnóstico de enfermedad grave por COVID - 19

C) Ensayo clínico controlado aleatorizado, doble ciego, fase III.

   a. Se aleatorizará de forma estratificada a sujetos que ingresen al INER con diagnóstico de enfermedad grave por COVID – 19. La aleatorización será central realizada en software dedicado. Los médicos tratantes, enfermería y resto del equipo tratante y los evaluadores de los desenlaces serán cegados al grupo que corresponden cada uno de los sujetos.
   b. Se estratificarán a los sujetos incluidos en dos grupos para la aleatorización:
      i. Grupo A: Sujetos con diagnostico de enfermedad grave por COVID 19 que requieran hospitalización.
      ii. Grupo B: Sujetos con diagnostico de enfermedad grave por COVID 19 que requieran manejo ventilatorio invasivo y/o manejo en la unidad de cuidados intensivos respiratorios al momento de la aleatorización.
   c. **Desenlace principal:**
      i. Mortalidad intrahospitalaria
   d. **Desenlaces secundarios:**
      i. Días de hospitalización
      ii. Necesidad de apoyo ventilatorio mecánico
      iii. Días de soporte ventilatorio mecánico.
      iv. Incidencia de efectos adversos grado 3 y 4.
   e. **Pruebas realizadas:**
      i. Panel de 10 virus respiratorios y de COVID-19 basal.
      ii. **Cada semana:** prueba viral RT-PCR en aspirado faríngeo, nasofaríngeo, o en muestra bronquial o de lavado bronquial o aspirado traqueal en intubados. Y semanalmente laboratorios como biometría hemática, química sanguínea y pruebas de funcionamiento hepático para seguimiento de reacciones adversas.
   f. **Intervenciones específicas del protocolo:**
      i. Los sujetos que ingresen al grupo experimental recibirán hidroxicloroquina por vía oral o por sonda nasogástrica, 400 mg al día por 10 días. El grupo placebo recibirá una tableta idéntica por 10 días. Se propone un periodo de tratamiento de 10 días principalmente por que no se conoce un periodo óptimo; los grupos de trabajo sugieren un uso entre 5 a 14 días; la caja de hidroxicloroquina contiene 20 tabletas de 200mg; justificamos el periodo de 10 días por encontrarse en el punto intermedio entre las varias recomendaciones y por ser más práctico utilizar una caja por sujeto

ii. No se intervendrá en ningún otro aspecto del manejo del sujeto.

ANALISIS ESTADÍSTICO

Se realizará el análisis en software estadístico R-Rstudio. Se utilizará estadística descriptiva de acuerdo al tipo de variable y distribución. Se comparará la mortalidad entre el grupo placebo e intervención mediante prueba exacta de fisher. Se compararán tiempos a los desenlaces y supervivencia global y por grupo ajustando por variables confusoras (edad, tiempo del inicio de síntomas al inicio de tratamiento, comorbilidades) mediante modelo de riesgos proporcionales de cox.

D) TAMAÑO DE MUESTRA

La mortalidad reportada en el COVID-19 grave se encuentra entre el 8% a incluso al 60% dependiendo la fuente[1,2] y la OMS sugiere al menos 400 sujetos para la estimación de la muestra en ensayos clínicos que evalúen terapias para el COVD-19 (3). Al momento se desconocen muchas características del comportamiento y desenlaces del COVID-19 especialmente en nuestro medio; por eso utilizamos la estimación de una mortalidad alrededor del 15% en COVID-19 grave con una reducción del 50% de la mortalidad con el tratamiento obteniendo en el cálculo de muestra un número de sujetos similar al recomendado por la OMS.

Nivel de significancia de 2 colas 95, alfa 5%
Potencia (1-beta, posibilidad de detección): 80%
Tratados vs controles 1:1
Mortalidad estimada en controles 15%
Mortalidad estimada en experimentales 7.5%, reducción de mortalidad de 7.5%, que es el 50% (RR 0.5).

<u>Estimaciones (Open-Epi)</u>

	Kelsey	Fleiss	Fleiss with CC	
Muestra experimental	280	279	305	
Muestra control		280	279	305
Muestra total :	560	558	610	

Se decide incluir 500 sujetos para el ensayo clínico.

E) <u>Criterios de inclusión</u>

- Firma de Consentimiento informado por el sujeto (o representante legal) entendiendo y aceptando los procedimientos y medicación del estudio descritos a continuación:
    - Aleatorización a hidroxocloroquina o placebo idéntico.
    - Recolección de muestra orofaringea (o aspirado traqueal o lavado bronquioalveolar) y muestras sanguíneas para confirmación y seguimiento de COVID – 19.
    - Hospitalización o ingreso a unidad de cuidados intensivos.
- En caso de ser mujer prueba de embarazo negativa.
- Al menos 18 años cumplidos al momento de la aleatorización.
- Confirmación por laboratorio de infección por SARS-CoV-2 determinada por RT-PCR de muestra faríngea, nasofaríngea, lavado broncoalveolar o aspirado traqueal antes de la aleatorización.
- Enfermedad grave por COVID – 19 definida por al menos uno de los siguientes:
    - SpO2 menor o igual a 90 % a una altura de 2240 m, en el valle de México o Puebla o disminución de más del 3% de saturación basal
    - Requiere ventilación mecánica y/o oxígeno suplementario (o incremento del apoyo de oxigeno suplementario en hipoxemia crónica)
    - Cumple criterios de sepsis/choque séptico.

<u>Criterios de exclusión:</u>

- Mayor de 80 años
- Hipersensibilidad conocida a hidroxicloroquina manifestada por anafilaxia.

- Consumo previo a la hospitalización de hidroxicloroquina o cloroquina.
- Decisión del médico responsable de excluir al participante del estudio.
- Antecedente de enfermedad hepática crónica. (Child-Pugh B o C).
- Antecedente de enfermedad renal crónica. (TFG menor o igual a 30).

. **Criterios de retiro/eliminación:**

- Decisión del médico responsable de retirar al participante del estudio.
- Transferencia del paciente a otra unidad hospitalaria.
- Hipersensibilidad nueva a hidroxicloroquina manifestada por anafilaxia.
- Efecto adverso grado 3 o 4
- Decisión del participante o representante de retirarse del estudio.

.

CONSIDERACIONES ÉTICAS

a) Indique el tipo de riesgo de la investigación.

El tipo de riesgo deberá ser considerado de a cuerdo a lo establecido en el Art. 17 del REGLAMENTO de la Ley General de Salud en Materia de Investigación para la Salud:

- Investigación sin riesgo.
- Investigación con riesgo mínimo.
- Investigación con riesgo mayor que el mínimo.

b) Para los protocolos de investigación en animales, hacer mención de la reglamentación vigente:

- Reglamento de la Ley General de Salud en Materia de Investigación para la Salud, Título Séptimo, de la Investigación que incluya la utilización de animales de experimentación.
- Ley Federal de Sanidad Animal.
- Reglamento de la Ley Federal de Sanidad Animal.
- NOM-062-ZOO-1999, Especificaciones técnicas para la producción, cuidado y uso de animales de laboratorio.
- Guía para el cuidado y uso de los animales de laboratorio “Guide for the Care and use of Laboratory Animals eigth Edition, Institute for Laboratory Animal Research Council”.
- Lineamientos para el sacrificio humanitario de los animales “AVMA Guidelines for the Euthanasia of Animals: 2013 Edition”.

COMENTARIOS Y CONSIDERACIONES DE LOS INVESTIGADORES
FORMACIÓN DE NUEVOS RECURSOS HUMANOS DURANTE EL PROYECTO
(INDICAR NIVEL)

No coresponde

REFERENCIAS BIBLIOGRÁFICAS

UTILIZAR LAS HOJAS NECESARIAS

Chan JF, Yuan S, Kok KH, To KK, Chu H, Yang J, Xing F, Liu J, Yip CC, Poon RW, Tsoi HW, Lo SK, Chan KH, Poon VK, Chan WM, Ip JD, Cai JP, Cheng VC, Chen H, Hui CK, Yuen KY. A familial cluster of pneumonia associated with the 2019 novel coronavirus indicating person-to-person transmission: a study of a family cluster. *Lancet* 2020.

2. Chen N, Zhou M, Dong X, Qu J, Gong F, Han Y, Qiu Y, Wang J, Liu Y, Wei Y, Xia J, Yu T, Zhang X, Zhang L. Epidemiological and clinical characteristics of 99 cases of 2019 novel coronavirus pneumonia in Wuhan, China: a descriptive study. *Lancet* 2020.
3. Holshue ML, DeBolt C, Lindquist S, Lofy KH, Wiesman J, Bruce H, Spitters C, Ericson K, Wilkerson S, Tural A, Diaz G, Cohn A, Fox L, Patel A, Gerber SI, Kim L, Tong S, Lu X, Lindstrom S, Pallansch MA, Weldon WC, Biggs HM, Uyeki TM, Pillai SK, Washington State -nCo VCIT. First Case of 2019 Novel Coronavirus in the United States. *The New England journal of medicine* 2020.
4. Zhou P, Yang XL, Wang XG, Hu B, Zhang L, Zhang W, Si HR, Zhu Y, Li B, Huang CL, Chen HD, Chen J, Luo Y, Guo H, Jiang RD, Liu MQ, Chen Y, Shen XR, Wang X, Zheng XS, Zhao K, Chen QJ, Deng F, Liu LL, Yan B, Zhan FX, Wang YY, Xiao GF, Shi ZL. A pneumonia outbreak associated with a new coronavirus of probable bat origin. *Nature* 2020.
5. Zhu N, Zhang D, Wang W, Li X, Yang B, Song J, Zhao X, Huang B, Shi W, Lu R, Niu P, Zhan F, Ma X, Wang D, Xu W, Wu G, Gao GF, Tan W, China Novel Coronavirus I, Research T. A Novel Coronavirus from Patients with Pneumonia in China, 2019. *The New England journal of medicine* 2020.
6. Centers of Disease Control. 2019 Novel Coronavirus. 2020 [cited 2020 February 7]; Available from: https://http://www.cdc.gov/coronavirus/2019-ncov/locations-confirmed-cases.html
7. Zhao S, Musa SS, Lin Q, Ran J, Yang G, Wang W, Lou Y, Yang L, Gao D, He D, Wang MH. Estimating the Unreported Number of Novel Coronavirus (2019-nCoV) Cases in China in the First Half of January 2020: A Data-Driven Modelling Analysis of the Early Outbreak. *Journal of clinical medicine* 2020: 9(2).
8. Wang M, Cao R, Zhang L, Yang X, Liu J, Xu M, Shi Z, Hu Z, Zhong W, Xiao G. Remdesivir and chloroquine effectively inhibit the recently emerged novel coronavirus (2019-nCoV) in vitro. *Cell research* 2020.
9. Savarino A. Use of chloroquine in viral diseases. *The Lancet Infectious diseases* 2011: 11(9): 653-654.
10. Savarino A, Boelaert JR, Cassone A, Majori G, Cauda R. Effects of chloroquine on viral infections: an old drug against today's diseases? *The Lancet Infectious diseases* 2003: 3(11): 722-727.
11. Yan Y, Zou Z, Sun Y, Li X, Xu KF, Wei Y, Jin N, Jiang C. Anti-malaria drug chloroquine is highly effective in treating avian influenza A H5N1 virus infection in an animal model. *Cell research* 2013: 23(2): 300-302.
12. Gao J, Tian Z, Yang X. Breakthrough: Chloroquine phosphate has shown apparent efficacy in treatment of COVID-19 associated pneumonia in clinical studies. *Bioscience trends* 2020.

DESGLOSE DE RECURSOS HUMANOS Y TÉCNICOS REQUERIDOS PARA EL ESTUDIO

Especificar aquellos que requieran ser autorizados por la Subdirección de Servicios Auxiliares de Diagnóstico y Paramédicos

RECURSO	NÚMERO REQUERIDO	ORIGEN O SITIO DONDE SE REALIZA	FUENTE DEL FINANCIAMIENTO
Un Investigador principal	1	INER	CONACYT
Subinvetigadores	8	INER	INER
Coordinador de estudio	1	INER	CONACYT
Ejecutadores	3	INER	CONACYT
Oxigeno	500	INER	INER
Hospitalizacion urgencias	500	INER	INER
Hospitalizacion pabellón	500	INER	INER
Hospitalizacion UCIR	500	INER	INER
Kits de laboratorio	500	INER	INER
Ventilación mecánica	500	INER	INER
Estudios de Tomografía	500	INER	INER
Medicamentos	500	INER	INER

<u>ÚNICAMENTE PARA PROTOCOLOS FINANCIADOS POR LA INDUSTRIA FARMACÉUTICA</u>
BENEFICIOS PARA LA INSTITUCIÓN

Explicar los beneficios: económicos para pacientes, infraestructura u otros, que tendrá el Instituto Nacional de Enfermedades Respiratorias Ismael Cosío Villegas, al participar en el presente protocolo de investigación.

RECOMENDACIONES PARA LLENADO DEL FORMATO

1. Todo proyecto de investigación que sea sometido a evaluación del Comité de Ética en Investigación o al Comité de Investigación, debe ajustarse al formato establecido.

2. Todos los proyectos deben ajustarse a los espacios requeridos, los cuales están diseñados para agilizar la revisión del proyecto.

3. Si algún apartado no aplica para un estudio en particular favor de indicarlo.

4. Es indispensable que siempre se llenen los apartados de consideraciones éticas y de desglose de recursos requeridos para el estudio.

5. En caso de estudios de investigación en humanos, siempre debe anexarse la carta de Consentimiento Informado.

6. Cualquier instrumento de nueva aplicación, como cuestionarios, etc., que se pretendan incluir en el estudio, se deberán anexar a este formato.

7. En caso de presentar a revisión protocolo en colaboración con Industria Farmacéutica es indispensable requisitar la última hoja, *Beneficios para la Institución.*

8. Únicamente se podrá extender en el apartado de *Métodos,* en caso de requerir utilizar más páginas, colocar el cursor por arriba de la línea, oprimir al mismo tiempo la tecla Ctrl y enter.

9. Es indispensable anotar el tipo de riesgo de la investigación, éste deberá ser considerado de acuerdo a lo establecido en el Art. 17 del REGLAMENTO de la Ley General de Salud en Materia de Investigación para la Salud.
   Investigación sin riesgo.
   Investigación con riesgo mínimo.
   Investigación con riesgo mayor que el mínimo.

TITULO DEL PROYECTO

HIDROXICLOROQUINA PARA EL TRATAMIENTO DE INFECCIÓN RESPIRATORIA GRAVE POR COVID-19: ENSAYO CLÍNICO CONTROLADO.

INVESTIGADOR RESPONSABLE Y ADSCRIPCIÓN

Debe contener: nombre, título y adscripción del investigador principal

Dra. Carmen Margarita Hernández Cárdenas
Jefe Áreas Críticas

INVESTIGADORES PARTICIPANTES Y ADSCRIPCIONES

Debe contener: nombres, títulos y adscripciones de los investigadores participantes.

Dr. Cristóbal Guadarrama Adscrito Unidad de Urgencias Respiratorias
Dr. Luis Felipe Jurado Camacho Adscrito Unidad de Cuidados Intensivos Respiratorios
Dr. José Rogelio Pérez Padilla Departamento en Investigación de Tabaquismo y EPOC
Dra. Ireri Isadora Thirión Romero Departamento en Investigación de Tabaquismo y EPOC
Dr. Joel Armando Vasquez Pérez Departamento en Investigación de Tabaquismo y EPOC

FECHA DE INICIO	FECHA DE TÉRMINO
10 – abril 2020	10 abril 2021

CRONOGRAMA

Periodos en: Meses: _______ Bimestres: _______ Otros _______

	1	2	3	4	5	6	7	8	9	10	11	21	13	14	15	16	17	18	19	20	21	22	23	24
Ejecución	x	x	x	x	x	x	X																	
Análisis								x	x	X														
Preparación de la publicación										x	x	x												

ÚLTIMAS 5 PUBLICACIONES Y/O PROYECTOS DEL INVESTIGADOR RESPONSABLE

Acute Respiratory Distress Syndrome Secondary to Influenza A(H1N1)pdm09: Clinical Characteristics and Mortality Predictors. Hernández-Cárdenas CM, Serna-Secundino H, García-Olazarán JG, Aguilar-Pérez CL, Rocha-Machado J, Campos-Calderón LF, Lugo-Goytia G. Rev Invest Clin. 2016 Sep-Oct;68(5):235-244. PMID: 27941959 Classifying Acute Respiratory Distress Syndrome Severity: Correcting the Arterial Oxygen Partial Pressure to Fractional Inspired Oxygen at Altitude. Pérez-Padilla R, Hernández-Cárdenas CM, Lugo-Goytia G. Rev Invest Clin. 2016 Jul-Aug;68(4):169-70. PMID: 27623033 Clinical Characteristics and Mortality of Influenza A H1N1 and Influenza-Like Illness in Mexico City in the 2013-2014 Winter Season. Martínez-Briseño D, Torre-Bouscoulet L, Herrera-Zamora Jde J, Díaz-Rico J, Sandoval-Macías G, Pérez-Padilla R, Hernández-Cárdenas C, Regalado-Pineda J, Salas-Hernández J, Santillán-Doherty P. Rev Invest Clin. 2016 May-Jun;68(3):147-53. PMID: 27409002 Influenza A (H1N1pdm09)-Related Critical Illness and Mortality in Mexico and Canada, 2014. Dominguez-Cherit G, De la Torre A, Rishu A, Pinto R, Ñamendys-Silva SA, Camacho-Ortiz A, Silva-Medina MA, Hernández-Cárdenas C, Martínez-Franco M, Quesada-Sánchez A, López-Gallegos GC, Mosqueda-Gómez JL, Rivera-Martinez NE, Campos-Calderón F, Rivero-Sigarroa E, Hernández-Gilsoul T, Espinosa-Pérez L, Macías AE, Lue-Martínez DM, Buelna-Cano C, Ramírez-García Luna AS, Cruz-Ruiz NG, Poblano-Morales M, Molinar-Ramos F, Hernandez-Torre M, León-Gutiérrez MA, Rosaldo-Abundis O, Baltazar-Torres JÁ, Stelfox HT, Light B, Jouvet P, Reynolds S, Hall R, Shindo N, Daneman N, Fowler RA.
Crit Care Med. 2016 Oct;44(10):1861-70. doi: 10.1097/CCM.0000000000001830.
PMID: 2735908 Population Pharmacokinetics of Gentamicin in Mexican Children with severe malnutrition Lares-Asseff I, Pérz-Guillé MG, Camacho Vieyra GA, Pérez AG, Peregrina NB, **Lugo Goytia** G. Pediatr Infect Dis J. 2016 Aug;35(8):872-8. doi: 10.1097/INF.0000000000001204. PMID: 27420805

TITULO DEL PROYECTO

HIDROXICLOROQUINA PARA EL TRATAMIENTO DE INFECCIÓN RESPIRATORIA GRAVE POR COVID-19: ENSAYO CLÍNICO CONTROLADO.

DEFINICIÓN DEL PROBLEMA

El COVID – 19 es un nuevo tipo de neumonía viral descrito en diciembre 2019 en Wuhan – China; representa un problema de salud público importante por su rápida propagación y la capacidad de generar neumonía grave en pacientes susceptibles; al momento no se a descrito ningún tratamiento específico para el virus siendo su manejo principalmente el soporte.

PREGUNTA DE INVESTIGACIÓN

¿El tratamiento por 10 días con 400 mg/día de hidroxicloroquina comparada con placebo reduce la mortalidad intrahospitalaria en sujetos con enfermedad grave por COVID - 19?

ANTECEDENTES

Se deberá proveer información de base para poder entender la relevancia del presente estudio y el propósito y fundamento del estudio.

Se deberá incluir un resumen de los datos de estudios no clínicos que tengan potencialmente una importancia clínica y de estudios clínicos y epidemiológicos relevantes para el presente estudio, así como las referencias de la literatura relevantes al estudio y que provean un sustento para el estudio.

El brote de infección respiratoria por el coronavirus 2019 (2019-nCOV) inició en diciembre del 2019, en la ciudad de Wuhan, provincia de Hubei (China) [1-5]. De esta ciudad, el brote se ha ido extendiendo a varias provincias de China y a otros países [6]. Hasta el 16 de Febrero del 2020, se habían reconocido 28 países con casos confirmados, con 70,548 infecciones y 1770 muertes en China, y 413 infecciones en Japón [6]. Como en otros coronavirus, el del SARS, y el del MERS, la infección original humana probablemente se obtuvo de contacto con animales, pero se ha demostrado una eficaz transmisión entre humanos, con una tasa de crecimiento mayor a 1, estimada de 2.6 [7], y una duplicación rápida de casos que aseguran no solo el mantenimiento de la epidemia si no su expansión.

Hasta el momento actual no se cuenta con un tratamiento efectivo disponible para el COVID-19, sin embargo in vitro, se reporto recientemente la efectividad in vitro del Remdesivir, y de la cloroquina, esta ultima en concentraciones capaces de ser obtenidas in vivo en pacientes que la usan [8]. La cloroquina e hidroxicloroquina, son medicamentos originalmente antimaláricos, en uso regular por décadas y con una toxicidad limitada, a un costo muy bajo, y en caso de ser efectivo in vivo, podría ser un medicamento de gran utilidad. El efecto antiviral amplio, se ha atribuido al incremento del pH de los endosomas, que se requieren para la fusión del virus con la célula y también por interferencia con la glicosilación de los receptores celulares del SARS-COV[9-11]
Adicionalmente al efecto antiviral, se le han atribuido efectos inmunomoduladores que podrían potenciar el efecto antiviral y el impacto sistémico de las infecciones graves.
La gama de presentaciones clínicas del 2019-CoV, es amplia, desde infecciones asintomáticas hasta infecciones fatales, con daño pulmonar grave, falla respiratoria, y falla multiorgánica. El daño grave se ha asociado, como en la influenza grave, a una reacción inflamatoria sistémica o una tormenta de citocinas, que podría ser atenuada por el efecto inmunomodulador de la cloroquina o hidroxicloroquina.

La tasa de letalidad general de la enfermedad por COVID-19 se ha estimado alrededor del 2%, pero en los casos graves en falla respiratoria, o requiriendo cuidados críticos, es considerablemente mayor, entre 10-15%, pero fácilmente podría incrementarse hasta el 20-30% dependiendo de los criterios de entrada a una unidad de terapia intensiva.
Recientemente se publicó una carta [12] que describe que en Febrero 17, el Consejo Estatal de China dio un aviso de prensa indicando que la Cloroquina habia demostrado marcada eficacia y seguridad aceptable para el tratamiento de la neumonía por COVID-19 en ensayos multicéntricos de China con más de 100 pacientes de 10 hospitales.

JUSTIFICACIÓN

Se carece de un tratamiento farmacológico aprobado para el COVID - 19, y basados en la efectividad in Vitro de la cloroquina, su seguridad demostrada por décadas, su costo bajo, se propone un ECC, doble ciego, para evaluar la eficacia y seguridad del compuesto derivado hidroxicloroquina en sujetos hospitalizados con enfermedad respiratoria grave por COVID - 19.

HIPÓTESIS

El tratamiento con Hidroxicloroquina 400 mg/día por 10 días reduce la mortalidad intrahospitalaria comparada con placebo en sujetos con enfermedad respiratoria grave por COVID-19.

OBJETIVO (S) GENERAL (ES)

Estimar si el tratamiento por 10 días con 400 mg/día de hidroxicloroquina reduce la mortalidad intrahospitalaria comparada con placebo en sujetos con enfermedad grave por COVID – 19.

OBJETIVOS ESPECÍFICOS

A) Días de hospitalización
B) Necesidad de apoyo ventilatorio mecánico
C) Días de soporte ventilatorio mecánico.
D) Incidencia de efectos adversos grado 3 y 4.

DISEÑO EXPERIMENTAL

1. INVESTIGACIÓN CLÍNICA x
2. INVESTIGACIÓN BÁSICA
3. INVESTIGACIÓN SOCIAL
4. INVESTIGACIÓN EPIDEMIOLÓGICA

FAVOR DE INDICAR EL TIPO DE ESTUDIO

OBSERVACIONAL
x EXPERIMENTAL
RETROSPECTIVO
x LONGITUDINAL
x PROSPECTIVO
TRANSVERSAL
OTRO FAVOR DE INDICAR:

"Indicar lo que se pretende obtener para el conocimiento en el tema de estudio al desarrollar esta investigación"

1. Comprensión
2. Prevención
3. Diagnóstico
4. Tratamiento x
5. Rehabilitación

Indicar la línea de investigación de la que se deriva este proyecto:

1. Programa de Estudio sobre la Influenza y otros Virus Respiratorios. x
2. Programa de Estudio sobre Problemas Respiratorios asociados a la exposición al Tabaco y Humo de Leña.
3. Programa de Estudio sobre Tuberculosis.
4. Programa de Estudio sobre Enfermedades Fibrosantes del Pulmón.
5. Programa de Estudio sobre Cáncer Pulmonar.
6. Programa de Estudio sobre Asma.
7. Programa de Estudio de la Clínica del Sueño.
8. Programa de Estudio sobre VIH y SIDA.
9. Programa de Estudio en Cirugía y Trasplante Pulmonar Experimental.
10. Programa de Estudio de Investigación en Inmunogenética y Alergia.
11. Programa de Estudio sobre Infecciones Respiratorias Agudas.
12. Programa de Estudio sobre Enfermedades del Oído y vías Respiratorias Altas.
13. Programa de Estudio sobre Enfermedades Reumáticas.
14. Programa de Estudio sobre Enfermedades Metabólicas asociadas a Enfermedades Respiratorias.
15. Programa de Estudio sobre Fisiología Respiratoria.
16. Programa de Estudio sobre Tromboembolia Pulmonar.
17. Programa de Estudio sobre Enfermedades Cardiovasculares y Respiratorias.
18. Programa de Investigación sobre Salud Respiratoria.
19. Programa de Estudio sobre Farmacología.
20. Programa de Desarrollo Biotecnológico.
21. Otro
    Especificar:

MATERIAL Y MÉTODOS

UTILIZAR LAS HOJAS NECESARIAS

A) Lugar del estudio
B) Descripción de la población de estudio
C) Procedimientos del estudio
D) Número necesario de sujetos de investigación
E) Criterios de inclusión y exclusión
F) Captura, procesamiento, análisis e interpretación de la información.

A) Áreas críticas y Hospitalización del Instituto Nacional de Enfermedades Respiratorias Ismael Cosío Villegas
B) Mayores de 18 años con diagnóstico confirmado de COVID – 19 por RT-PCR por muestra faríngea, naso-faríngea o en aspirado traqueal/lavado bronquioalveolar, con diagnóstico de enfermedad grave por COVID - 19
C) Ensayo clínico controlado aleatorizado, doble ciego, fase III.
   a. Se aleatorizará de forma estratificada a sujetos que ingresen al INER con diagnóstico de enfermedad grave por COVID – 19. La aleatorización será central realizada en software dedicado. Los médicos tratantes, enfermería y resto del equipo tratante y los evaluadores de los desenlaces serán cegados al grupo que corresponden cada uno de los sujetos.
   b. Se estratificarán a los sujetos incluidos en dos grupos para la aleatorización:
      i. Grupo A: Sujetos con diagnostico de enfermedad grave por COVID 19 que requieran hospitalización.
      ii. Grupo B: Sujetos con diagnostico de enfermedad grave por COVID 19 que requieran manejo ventilatorio invasivo y/o manejo en la unidad de cuidados intensivos respiratorios al momento de la aleatorización.
   c. **Desenlace principal:**

En sujetos del grupo A: Proporción de pacientes que requieren ventilación mecánica invasiva y/o tasa de muerte a 30 días posterior a la aleatorización
En sujetos del grupo B: Duración en días de ventilación mecánica invasiva o tasa de muerte a 30 días posterior a la aleatorización

   d. **Desenlaces secundarios:**
      i. Días de hospitalización
      ii. Necesidad de apoyo ventilatorio mecánico
      iii. Días de soporte ventilatorio mecánico.
      iv. Incidencia de efectos adversos grado 3 y 4.
   e. **<u>Pruebas realizadas:</u>**
      i. Panel de 10 virus respiratorios y de COVID-19 basal, laboratorios como biometría hemática, química sanguínea y pruebas de funcionamiento hepático que corresponden a una toma de muestra venosa de 5 mL el equivalente a una cucharada de postre.
      ii. **<u>Cada semana:</u>** prueba viral RT-PCR en aspirado faríngeo, nasofaríngeo, o en muestra bronquial o de lavado bronquial o aspirado traqueal en intubados. Y semanalmente laboratorios como biometría hemática, química sanguínea y pruebas de funcionamiento hepático -que corresponden a una toma de muestra venosa de 5 mL el equivalente a una cucharada de postre- para seguimiento de reacciones adversas. Dentro de esta toma sanguínea en el tubo de química sanguínea se analizará kit de citosinas al primer y séptimo día.

f. **Intervenciones específicas del protocolo:**

i. Los sujetos que ingresen al grupo experimental recibirán hidroxicloroquina por vía oral o por sonda nasogástrica, 400 mg al día por 10 días. El grupo placebo recibirá una tableta idéntica por 10 días. Se propone un periodo de tratamiento de 10 días principalmente por que no se conoce un periodo óptimo; los grupos de trabajo sugieren un uso entre 5 a 14 días; la caja de hidroxicloroquina contiene 20 tabletas de 200mg; justificamos el periodo de 10 días por encontrarse en el punto intermedio entre las varias recomendaciones y por ser más práctico utilizar una caja por sujeto

Seguimiento: El seguimiento durará hasta el alta hospitalaria.
Los efectos adversos serán reportados al comité de ética, COFEPRIS y al productor del fármaco (Sanofi México, S.A. de C.V.)

ANALISIS ESTADÍSTICO

Se realizará el análisis en software estadístico R-Rstudio. Se utilizará estadística descriptiva de acuerdo al tipo de variable y distribución. Se comparará la mortalidad entre el grupo placebo e intervención mediante prueba exacta de fisher. Se compararán tiempos a los desenlaces y supervivencia global y por grupo ajustando por variables confusoras (edad, tiempo del inicio de síntomas al inicio de tratamiento, comorbilidades) mediante modelo de riesgos proporcionales de cox.

Análisis:

- La tasa de mortalidad (30 días), la proporción de pacientes que necesitan ventilación mecánica y los días de ventilación mecánica, se compararán en un modelo multivariado, que incluye un indicador de estratos de aleatorización, ajustando las variables de confusión (edad, tiempo desde el inicio de los síntomas hasta el inicio de tratamiento, comorbilidades), se realizará una comparación similar para los resultados secundarios: duración de la hospitalización, duración de la oxigenoterapia.
- También compararemos el tiempo hasta la muerte ajustando las variables de confusión (edad, tiempo desde el inicio de los síntomas hasta el inicio del tratamiento, comorbilidades) utilizando el modelo de riesgos proporcionales de Cox.
- Habrá un CMDS, un grupo asesor multidisciplinario, compuesto por un experto en estadística, un investigador clínico, un experto en ética y un clínico relacionado con la influenza y el coronavirus, ninguno involucrado en el ensayo clínico y, por lo tanto, independiente, que en el medio de los ensayos tendrá acceso a los datos y al código de aleatorización para evaluar los datos, incluida la seguridad y los resultados.

  Investigador clínico: Gustavo Lugo, farmacólogo clínico, anestesiólogo e intensivista que actuará como coordinador. Dra. Angélica Portillo, especialista en oído, nariz y garganta y con una maestría en ciencias médicas.

  Estadísticas: Dra. Rosario Fernández, Jefa del Departamento de Epidemiología del INER.

  Ética: Dr. Patricio Santillán, director médico de INER.

  Clínico: Dr. Arturo Martínez, Infectólogo, experto en infecciones similares a la gripe y ahora en infección por COVID-19.

D) TAMAÑO DE MUESTRA

La mortalidad reportada en el COVID-19 grave se encuentra entre el 8% a incluso al 60% dependiendo la fuente[1,2] y la OMS sugiere al menos 400 sujetos para la estimación de la muestra en ensayos clínicos que evalúen terapias para el COVD-19 (3). Al momento se desconocen muchas características del comportamiento y desenlaces del COVID-19 especialmente en nuestro medio; por eso utilizamos la estimación de una mortalidad alrededor del 15% en COVID-19 grave con una reducción del 50% de la mortalidad con el tratamiento obteniendo en el cálculo de muestra un número de sujetos similar al recomendado por la OMS.

Nivel de significancia de 2 colas 95, alfa 5%

Potencia (1-beta, posibilidad de detección): 80%

Tratados vs controles 1:1

Mortalidad estimada en controles 15%

Mortalidad estimada en experimentales 7.5%, reducción de mortalidad de 7.5%, que es el 50% (RR 0.5).

<u>Estimaciones (Open-Epi)</u>

	Kelsey	Fleiss	Fleiss with CC	
Muestra experimental	280	279	305	
Muestra control		280	279	305
Muestra total :	560	558	610	

Se decide incluir 600 sujetos para el ensayo clínico, es decir 300 sujetos en el grupo de tratamiento experimental y 300 sujetos en el grupo control.

E) <u>Criterios de inclusión</u>

E) Firma de Consentimiento informado por el sujeto (o representante legal) entendiendo y aceptando los procedimientos y medicación del estudio descritos a continuación:
   a. Aleatorización a hidroxocloroquina o placebo idéntico.
   b. Recolección de muestra orofaringea (o aspirado traqueal o lavado bronquioalveolar) y muestras sanguíneas para confirmación y seguimiento de COVID – 19.
   c. Hospitalización o ingreso a unidad de cuidados intensivos.

F) En caso de ser mujer prueba de embarazo negativa.

G) Inicio de síntomas menor a 14 días.

H) Al menos 18 años cumplidos al momento de la aleatorización.

I) Confirmación por laboratorio de infección por SARS-CoV-2 determinada por RT-PCR de muestra faríngea, nasofaríngea, lavado broncoalveolar o aspirado traqueal antes de la aleatorización y/o infección pulmonar documentada en placa de tórax o tomografía definida por lesiones difusas predominantemente periféricas y subpleurales del tipo consolidación y vidrio deslustrado.

J) Enfermedad grave por COVID – 19 definida por al menos uno de los siguientes:
   a. SpO2 menor o igual a 90 % a una altura de 2240 m, en el valle de México o Puebla o disminución de más del 3% de saturación basal
   b. Requiere ventilación mecánica y/o oxígeno suplementario (o incremento del apoyo de oxigeno suplementario en hipoxemia crónica)
   c. Cumple criterios de sepsis/choque séptico.

<u>Criterios de exclusión:</u>

K) Mayor de 80 años

L) Hipersensibilidad conocida a hidroxicloroquina manifestada por anafilaxia.

M) Consumo previo a la hospitalización de hidroxicloroquina o cloroquina. Tratamiento actual o del mes pasado.

N) Decisión del médico responsable de excluir al participante del estudio.

O) Antecedente de enfermedad hepática crónica. (Child-Pugh B o C).

P) Antecedente de enfermedad renal crónica. (TFG menor o igual a 30).

Q) Prueba de embarazo positiva.
R) Contraindicación para iniciar o continuar con hidroxicloroquina (incluidos, entre otros, antecedentes de anafilaxia, un QTc> 450 / 500mmseg por ECG, interacción farmacológica peligrosa o patología retiniana).
S) Infección previa por COVID-19
T) Traslado del paciente a otra unidad hospitalaria.

. **Criterios de retiro/eliminación:**

U) Decisión del médico responsable de retirar al participante del estudio.
V) Transferencia del paciente a otra unidad hospitalaria.
W) Hipersensibilidad nueva a hidroxicloroquina manifestada por anafilaxia.
X) Efecto adverso grado 3 o 4
Y) Decisión del participante o representante de retirarse del estudio.

Centros que pueden participar además del INER (Centros COVID)

- Instituto Nacional de Ciencias Médicas y Nutrición "Salvador Zubirán"
- Hospital Juárez de México
- Hospital General de México "Dr. Eduardo Liceaga"
- Hospital Regional de Alta Especialidad de Iztapaluca
- Hospital Regional de Alta Especialidad de Mérida
- Hospital Regional de Alta Especialidad de Oaxaca

CONSIDERACIONES ÉTICAS

a) Indique el tipo de riesgo de la investigación.

El tipo de riesgo deberá ser considerado de a cuerdo a lo establecido en el Art. 17 del REGLAMENTO de la Ley General de Salud en Materia de Investigación para la Salud:

- Investigación sin riesgo.
- Investigación con riesgo mínimo.
- Investigación con riesgo mayor que el mínimo.

b) Para los protocolos de investigación en animales, hacer mención de la reglamentación vigente:

- Reglamento de la Ley General de Salud en Materia de Investigación para la Salud, Título Séptimo, de la Investigación que incluya la utilización de animales de experimentación.
- Ley Federal de Sanidad Animal.
- Reglamento de la Ley Federal de Sanidad Animal.
- NOM-062-ZOO-1999, Especificaciones técnicas para la producción, cuidado y uso de animales de laboratorio.
- Guía para el cuidado y uso de los animales de laboratorio “Guide for the Care and use of Laboratory Animals eigth Edition, Institute for Laboratory Animal Research Council”.
- Lineamientos para el sacrificio humanitario de los animales “AVMA Guidelines for the Euthanasia of Animals: 2013 Edition”.

Este estudio implica un **riesgo mayor al mínimo** para el participante; esto quiere decir que los riesgos esperados son superiores a los relacionados con el tratamiento que tendría si decidiera no participar; los riesgos potenciales son: posibles reacciones adversas a la hidroxicloroquina, posibles lesiones asociadas con la toma de muestra nasofaríngea y posibles lesiones asociadas a la toma de muestras venosas.

COMENTARIOS Y CONSIDERACIONES DE LOS INVESTIGADORES
FORMACIÓN DE NUEVOS RECURSOS HUMANOS DURANTE EL PROYECTO

(INDICAR NIVEL)

No coresponde

REFERENCIAS BIBLIOGRÁFICAS

UTILIZAR LAS HOJAS NECESARIAS

1. Chan JF, Yuan S, Kok KH, To KK, Chu H, Yang J, Xing F, Liu J, Yip CC, Poon RW, Tsoi HW, Lo SK, Chan KH, Poon VK, Chan WM, Ip JD, Cai JP, Cheng VC, Chen H, Hui CK, Yuen KY. A familial cluster of pneumonia associated with the 2019 novel coronavirus indicating person-to-person transmission: a study of a family cluster. *Lancet* 2020.

2. Chen N, Zhou M, Dong X, Qu J, Gong F, Han Y, Qiu Y, Wang J, Liu Y, Wei Y, Xia J, Yu T, Zhang X, Zhang L. Epidemiological and clinical characteristics of 99 cases of 2019 novel coronavirus pneumonia in Wuhan, China: a descriptive study. *Lancet* 2020.

3. Holshue ML, DeBolt C, Lindquist S, Lofy KH, Wiesman J, Bruce H, Spitters C, Ericson K, Wilkerson S, Tural A, Diaz G, Cohn A, Fox L, Patel A, Gerber SI, Kim L, Tong S, Lu X, Lindstrom S, Pallansch MA, Weldon WC, Biggs HM, Uyeki TM, Pillai SK, Washington State -nCo VCIT. First Case of 2019 Novel Coronavirus in the United States. *The New England journal of medicine* 2020.

4. Zhou P, Yang XL, Wang XG, Hu B, Zhang L, Zhang W, Si HR, Zhu Y, Li B, Huang CL, Chen HD, Chen J, Luo Y, Guo H, Jiang RD, Liu MQ, Chen Y, Shen XR, Wang X, Zheng XS, Zhao K, Chen QJ, Deng F, Liu LL, Yan B, Zhan FX, Wang YY, Xiao GF, Shi ZL. A pneumonia outbreak associated with a new coronavirus of probable bat origin. *Nature* 2020.

5. Zhu N, Zhang D, Wang W, Li X, Yang B, Song J, Zhao X, Huang B, Shi W, Lu R, Niu P, Zhan F, Ma X, Wang D, Xu W, Wu G, Gao GF, Tan W, China Novel Coronavirus I, Research T. A Novel Coronavirus from Patients with Pneumonia in China, 2019. *The New England journal of medicine* 2020.

6. Centers of Disease Control. 2019 Novel Coronavirus. 2020 [cited 2020 February 7]; Available from: https://http://www.cdc.gov/coronavirus/2019-ncov/locations-confirmed-cases.html

7. Zhao S, Musa SS, Lin Q, Ran J, Yang G, Wang W, Lou Y, Yang L, Gao D, He D, Wang MH. Estimating the Unreported Number of Novel Coronavirus (2019-nCoV) Cases in China in the First Half of January 2020: A Data-Driven Modelling Analysis of the Early Outbreak. *Journal of clinical medicine* 2020: 9(2).

8. Wang M, Cao R, Zhang L, Yang X, Liu J, Xu M, Shi Z, Hu Z, Zhong W, Xiao G. Remdesivir and chloroquine effectively inhibit the recently emerged novel coronavirus (2019-nCoV) in vitro. *Cell research* 2020.

9. Savarino A. Use of chloroquine in viral diseases. *The Lancet Infectious diseases* 2011: 11(9): 653-654.

10. Savarino A, Boelaert JR, Cassone A, Majori G, Cauda R. Effects of chloroquine on viral infections: an old drug against today's diseases? *The Lancet Infectious diseases* 2003: 3(11): 722-727.

11. Yan Y, Zou Z, Sun Y, Li X, Xu KF, Wei Y, Jin N, Jiang C. Anti-malaria drug chloroquine is highly effective in treating avian influenza A H5N1 virus infection in an animal model. *Cell research* 2013: 23(2): 300-302.

12. Gao J, Tian Z, Yang X. Breakthrough: Chloroquine phosphate has shown apparent efficacy in treatment of COVID-19 associated pneumonia in clinical studies. *Bioscience trends* 2020.

DESGLOSE DE RECURSOS HUMANOS Y TÉCNICOS REQUERIDOS PARA EL ESTUDIO

Especificar aquellos que requieran ser autorizados por la Subdirección de Servicios Auxiliares de Diagnóstico y Paramédicos

RECURSO	NÚMERO REQUERIDO	ORIGEN O SITIO DONDE SE REALIZA	FUENTE DEL FINANCIAMIENTO
Investigador principal	1	INER	INER
Subinvetigadores	8	INER	INER
Coordinador de estudio	1	INER	CONACYT
Ejecutadores	3	INER	CONACYT
Oxígeno	500	INER	INER
Hospitalización urgencias	500	INER	INER
Hospitalización pabellón	500	INER	INER
Hospitalización UCIR	500	INER	INER
Kits de laboratorio	500	INER	INER
Ventilación mecánica	500	INER	INER
Medicamentos	500	INER	INER

ÚNICAMENTE PARA PROTOCOLOS FINANCIADOS POR LA INDUSTRIA FARMACÉUTICA
BENEFICIOS PARA LA INSTITUCIÓN

Explicar los beneficios: económicos para pacientes, infraestructura u otros, que tendrá el Instituto Nacional de Enfermedades Respiratorias Ismael Cosío Villegas, al participar en el presente protocolo de investigación.

CONSENTIMIENTO INFORMADO PARA PARTICIPAR EN UN ESTUDIO DE INVESTIGACIÓN MÉDICA

Título del estudio:“*Hidroxicloroquina para el tratamiento de infección respiratoria grave por COVID-19: ensayo clínico controlado”.*

Investigador Principal: Dra. Carmen Margarita Hernández Cárdenas.

Sede dónde se realizará el estudio: Instituto nacional de Enfermedades Respiratorias Ismael Cosío Villegas

Nombre del participante: ____

A usted se le está invitando a participaren este estudio de investigación médica. Antes de decidir si acepta o no, debe conocer y comprender cada uno de los siguientes apartados. Este proceso se conoce como consentimiento informado. Siéntase con absoluta libertad para preguntar sobre cualquier aspecto que le ayude a aclarar sus dudas al respecto.

¨El presente estudio tiene el objetivo de evaluar si la hidroxicloroquina es un tratamiento ***eficaz y seguro*** *en pacientes con enfermedad pulmonar gravepor COVID -19 (también conocido como coronavirus)”.*

“Al momento no se cuenta con un tratamiento para esta enfermedad por lo que estudiar la hidroxicloroquina podría ser beneficioso para usted yotros sujetos con enfermedad pulmonar grave por COVID-19 o coronavirus¨.

“Los beneficios que esperan encontrarse con la hidroxicloroquina en el tratamiento de la enfermedad pulmonar grave por COVID-19 o coronavirus son los siguientes: menor riesgo de morir por complicaciones, menor tiempo de internamiento en el hospital, menor número de ***complicaciones pulmonares graves****”*

Nota: “¿Qué son las ***complicaciones pulmonares graves****?: Cuando una persona sufre de una enfermedad respiratoria grave como el COVID-19 o coronavirus sus pulmones pueden dañarse tanto que no son capaces de proveer oxigeno a la sangre; cuando el nivel de oxígeno en la sangre llega a niveles muy bajos es necesario remplazar el funcionamiento de los pulmones con aparatos conocidos como* ***ventiladores mecánicos****; esta es una complicación muy grave de la enfermedad donde el riesgo de morir es mucho más elevado”.*

CONSENTIMIENTO INFORMADO PARA PARTICIPAR EN UN ESTUDIO DE INVESTIGACIÓN MÉDICA

Título del estudio: “*Hidroxicloroquina para el tratamiento de infección respiratoria grave por COVID-19: ensayo clínico controlado”.*

“Su participación en el estudio, las pruebas de laboratorio y tratamiento serán gratuitos y la información se mantendrá resguardada como confidencia tomando todas las medidas de protección necesarias”.

Una vez que haya comprendido el estudio y si usted desea participar se le pedirá que firme esta carta de consentimiento, de la cual se le entregará una copia firmada y fechada.**Cabe destacar que la participación en este estudio o la negación a participar no intervendrá de ninguna forma en su tratamiento y seguimiento y que cualquier momento puede decidir dejar de participar en el estudio.**

Su participación consiste en:

*“Si usted desea participar voluntariamente y firma esta carta de consentimiento procederemos a asignarle a uno de dos grupos al **azar**; el primer grupo recibirá **hidroxicloroquina**400mg cada 24 horas por vía oral durante 10 días y el segundo grupo recibirá un **placebo** idéntico cada 24 horas por 10 días. Para poder lograr nuestros objetivos requerimos la participación de 500 sujetos”*

*Nota: ¿Que es el **azar** y por qué se asigna quien recibirá el tratamiento de esta forma?: La forma para evaluar nuevos tratamientos está establecida por normas y organismos tanto internacionales como nacionales; se asigna el tratamiento al azar para evitar que tanto el participante como el grupo que analiza los datos y los resultados conozcan quien recibe el tratamiento y quien el placebo; de esta manera puede confirmarse la utilidad del tratamiento sin la posibilidad de que existan otros factores que modifiquen los resultados; el azar se establece mediante programas de computación que indican que sujeto recibirá tratamiento o placebo; esto quiere decir que nadie puede saber de antemano que grupo le corresponderá.*

*Nota: ¿Qué es la **hidroxicloroquina**? La hidroxicloroquina es un medicamente ampliamente estudiado y usado para otras enfermedades como la malaria y la artritis reumatoide; se cree que la hidroxicloroquina podría tener efectos sobre el virus que produce la enfermedad COVID-19 o coronavirus, pero antes de poder utilizar este tratamiento en todos los sujetos con esta enfermedad debe confirmarse su eficacia y seguridad en un estudio como el que está siendo invitado a participar.*

CONSENTIMIENTO INFORMADO PARA PARTICIPAR EN UN ESTUDIO DE INVESTIGACIÓN MÉDICA

Título del estudio: "*Hidroxicloroquina para el tratamiento de infección respiratoria grave por COVID-19: ensayo clínico controlado".*

*Nota: ¿Qué es un **placebo**? Un placebo es un medicamento similar a la hidroxicloroquina en forma, sabor y olor, pero que no tiene ningún efecto ni beneficioso ni nocivo; puede estar hecho de sustancias como azúcar o almidón; un nuevo tratamiento siempre debe compararse con placebo para saber si su efecto se puede atribuir al medicamento o a otros factores.*

"Se tomarán datos de su expediente clínico relacionados con su tratamiento y seguimiento además de datos personales relacionados con su estado de salud actual y previo, garantizando suconfidencialidad.".

*"Se tomarán muestras semanales para detectar COVID-19 o coronavirus en muestras obtenidas de **hisopado nasofaríngeo** o de secreciones respiratorias durante su hospitalización".*

*Nota: ¿Qué es el **hisopadonasofaríngeo**? Es una técnica para tomar muestras de vía aérea que consiste en introducir un hisopo por la nariz hasta la rinofaringe; esta se enviará a un laboratorio para determinar la presencia de COVID-19.*

"Se tomarán muestras de sangre por punción venosa, llamadas biometría hemática y química sanguínea durante su hospitalización, la cantidad de sangre tomada será de 5 mL el equivalente a una cucharada de postre, cada día por 10 días y posteriormente y hasta su alta hospitalaria de forma semanal; estas pruebas se realizarán para buscar si presenta efectos adversos a la medicación y evalúan la función de sus riñones y de su hígado".

"Lo único que se modificará en su tratamiento es el medicamento llamado hidroxicloroquina o placebo comentado anteriormente, el resto del tratamiento siempre será decidido por su equipo médico y nosotros no tendremos influencia en él"

"El estudio concluirá el momento de su alta hospitalaria; no asistirá a ninguna consulta adicional por parte de este estudio".

"Sus responsabilidades como participante se limitan a concluir con el tratamiento (a menos que desee retirarse del estudio) y compartir datos relacionados con sus antecedentes de salud sin faltar a la verdad"

Calzada de Tlalpan No. 4502, Col. Sección XVI, Deleg. Tlalpan, México, D.F., C.P. 14080
Tel. (55) 54 87 17 00 www.iner.salud.gob.mx

CONSENTIMIENTO INFORMADO PARA PARTICIPAR EN UN ESTUDIO DE INVESTIGACIÓN MÉDICA

Título del estudio: "*Hidroxicloroquina para el tratamiento de infección respiratoria grave por COVID-19: ensayo clínico controlado".*

Riesgos asociados con el estudio: Este estudio implica un **riesgomayor al mínimo** para el participante; esto quiere decir que los riesgos esperados son superiores a los relacionados con el tratamiento que tendría si decidiera no participar; los riesgos potenciales son: posibles reacciones adversas a la hidroxicloroquina, posibles lesiones asociadas con la toma de muestra nasofaríngea y posibles lesiones asociadas a la toma de muestras venosas.

*"La **hidroxicloroquina** es un medicamento conocido y relativamente seguro, algunas personas pueden experimentar **molestias abdominales** manifestadas por **nauseas**, **vómitos** y/o **diarrea**; estos efectos son leves y se resuelven de forma espontánea al terminar el tratamiento o con medidas sintomáticas".*

*"En algunos casos se puede encontrar perdida parcial de la visión, es decir, usted puede empezar a ver borroso, a esto se le llama **retinopatía progresiva** (1 al 10% tras cinco años de consumo de hidroxicloroquina y con dosis mucho mayores a las administradas en este estudio) se limita la frecuencia de esta complicación al usar una dosis de 400mg al día por sólo 10 días; se realizará una evaluación por oftalmología sólo en caso de presentar molestias visuales ya que se aconseja monitorizar esta complicación a partir de un año de tratamiento; existen grupos con un riesgo mayor para esta complicación como ser sujetos con enfermedad crónica del hígado y enfermedad crónica del riñón; por lo que si usted presenta alguna de esas enfermedades no será invitado a participar".*

*"Los potenciales riesgos de la toma de un **hisopado nasofaríngeo** son molestias por el procedimiento manifestadas por tos y lagrimeo y en casos raros sangrado nasal que se limita realizando presión sobre la nariz"*

*"Los potenciales riesgos de la **punción venosa** son dolor y hematoma en el área de punción, esta complicación se limita realizando presión sobre la vena tras la punción y se resuelve espontáneamente"*

Calzada de Tlalpan No. 4502, Col. Sección XVI, Deleg. Tlalpan, México, D.F., C.P. 14080
Tel. (55) 54 87 17 00 www.iner.salud.gob.mx

CONSENTIMIENTO INFORMADO PARA PARTICIPAR EN UN ESTUDIO DE INVESTIGACIÓN MÉDICA

Título del estudio: “*Hidroxicloroquina para el tratamiento de infección respiratoria grave por COVID-19: ensayo clínico controlado*”.

“Se toman medidas para que su información personal esté protegida y no sea usada por personas no autorizadas; todos los archivos son almacenados bajo llave y los documentos electrónicos protegidos mediante contraseña; sólo las personas involucradas en la planificación, supervisión, monitoreo o auditorias del estudio tendrán acceso a la información”.

“Toda publicación de este ensayo utilizará información sin identificar a los participantes; las organizaciones que podrían inspeccionar y/o copiar la información para evaluación de la calidad y el análisis de los datos se limitan a grupos como el Comité de Ética en Investigación Institucional y otras autoridades regulatorias pertinentes”.

Aclaraciones:

- Su decisión de aceptar o no en el estudio es completamente voluntaria.
- No habrá ninguna consecuencia desfavorable en caso de no aceptar la invitación o en el caso de que desee retirarse del estudio una vez iniciado este.
- Si decide autorizar su participación en el estudio; usted puede solicitar retirarse del mismo en el momento que lo desee pudiendo informar o no las razones de su decisión, las cuales será respetada en su integridad.
- No tendrá un costo extra a la atención habitual
- No recibirá pago por su participación.
- En el transcurso del estudio ustedpodrá solicitar información actualizada sobre el mismo al investigador responsable y podrá retirarse del estudio si lo desea.

Calzada de Tlalpan No. 4502, Col. Sección XVI, Deleg. Tlalpan, México, D.F., C.P. 14080
Tel. (55) 54 87 17 00 www.iner.salud.gob.mx

CARTA DE CONSENTIMIENTO INFORMADO

Título del estudio: "*Hidroxicloroquina para el tratamiento de infección respiratoria grave por COVID-19: ensayo clínico controlado".*

- La información obtenida en este estudio, utilizada para la identificación de cada paciente, será mantenida con estricta confidencialidad por el grupo de investigadores.
- El estudio incluye un sistema de monitoreo interno con reglas para suspensión o eliminación de sujetos que estén participando; si usted u otro participante presenta algún tipo de reacción adversa grave durante el estudio se procederá a informarle, terminar con su participación con el estudio e iniciar el seguimiento y tratamiento de la complicación en caso de ser necesario.

Si considera que no hay dudas ni preguntas acerca de su participación, puede, si así lo desea, firmar la Carta de Consentimiento Informado que forma parte de este documento

Para dudas sobre sus derechos o el protocolo de estudio en general, comentarios, problemas relacionados con el estudio, reclamos o aclaraciones favor de contactarse con:

Contacto Investigador Principal: Dra. Carmen Hernández 54871700ext 5213
Contacto Presidenta Comité de Ética: Lic. Adriana Espinosa Jove 54871700 ext 5254

CARTA DE CONSENTIMIENTO INFORMADO

Título del estudio: “*Hidroxicloroquina para el tratamiento de infección respiratoria grave por COVID-19: ensayo clínico controlado”.*

Yo, ____________________________________ he leído y comprendido la información anterior y mis preguntas han sido respondidas de manera satisfactoria. He sido informado y entiendo que los datos obtenidos en el estudio pueden ser publicados o difundidos con fines científicos. Convengo en autorizar mi participación en este estudio de investigación. Recibiré una copia firmada y fechada de esta forma de consentimiento.

_____________________________________ _____________________
(Participante o nombre y firma de representante legal)
Firma Fecha

_____________________________________ _____________________
Testigo 1:Nombre y firma Dirección
Relación con el participante______________ Fecha______________

_____________________________________ _____________________
Testigo 2: Nombre y firma Dirección
Relación con el participante ______________ Fecha______________

Esta parte debe ser completada por el Investigador (o su representante):

He explicado al Sr(a). ___________________ la naturaleza y los propósitos de la investigación; le he explicado acerca de los riesgos y beneficios que implica la participaciónen el estudio. He contestado a las preguntas en la medida de lo posible y he preguntado si tiene alguna duda. Acepto que he leído y conozco la normatividad correspondiente para realizar investigación con seres humanos y me apego a ella.

Una vez concluida la sesión de preguntas y respuestas, se procedió a firmar el presente documento.

_____________________________________ _____________________

Nombre y firma del investigador Fecha

MANUAL DE PROCEDIMIENTOS
HIDROXICLOROQUINA PARA EL TRATAMIENTO DE INFECCIÓN RESPIRATORIA GRAVE POR COVID-19: ENSAYO CLÍNICO CONTROLADO.
Número de aprobación por comité Institucional de Ética:
Centro Coordinador:
Instituto Nacional de Enfermedades Respiratorias Ismael Cosío Villegas

Contenidos

Introducción 2
Resumen del protocolo: 2
Tipo de investigación 2
Planteamiento del problema 2
Antecedentes 3
Justificación 3
Hipótesis 3
Objetivo primario y secundario 3
Metodología 4
Personal del estudio y responsabilidades 4
Cronograma de evaluaciones 5
Reclutamiento y plan de retención 5
Reclutamiento 5
Criterios de inclusión 5
Criterios de exclusión: 6
Criterios de retiro/eliminación: 6
Plan de compensación para sujetos 7
Intervención del estudio 7
Aleatorización 7
Cegado y fin del cegado 8
Reporte de seguridad 8
Riesgos potenciales conocidos 8
Riesgos potenciales de la hidroxicloroquina 8
Riesgos a la privacidad 8
Retención 9
Colección de datos y forma de reporte de caso 9
Instrucciones generales para completar las formas de reporte de caso 9

Instrucciones generales para completar la base de datos 10
Almacenamiento de documentos de estudio 10
Manejo de datos *10*
Procedimientos de control de calidad 10
Actividades de monitoreo de seguridad de datos y seguridad 11
Procedimientos de confidencialidad 11
Seguridad 11
Operacionalización de variables *11*
Plan de análisis de datos detallado *19*
Estadística descriptiva y bivariada. 19

Introducción:

El COVID–19 es un nuevo tipo de neumonía viral descrito en diciembre 2019 en Wuhan – China; representa un problema de salud pública importante por su rápida propagación y la capacidad de generar neumonía grave en pacientes susceptibles; al momento no se a descrito ningún tratamiento específico para el virus siendo su manejo principalmente el soporte vital en los casos más graves. Se describió la actividad in vitro de la cloroquina contra el virus SARS-COV pero al momento no se cuenta con evidencia de que este tratamiento ni su derivado hidroxicloroquina pueda disminuir la presencia de desenlaces clínicos de interés (requerimiento de soporte ventilatorio invasivo, mortalidad, tiempo de asistencia ventilatoria mecánica invasiva y tiempo de hospitalización) en sujetos con enfermedad grave por COVID – 19; el objetivo del ensayo es estimar si es que el tratamiento con hidroxicloroquina comparado con placebo en enfermedad pulmonar grave por COVID – 19 puede disminuir la mortalidad intrahospitalaria.

Resumen del protocolo:

Tipo de investigación
Ensayo clínico controlado y aleatorizado doble ciego diseñado para evaluar la seguridad y eficacia de la hidroxicloroquina para el tratamiento de la enfermedad grave por COVID – 19.

Planteamiento del problema
El COVID – 19 es un nuevo tipo de neumonía viral descrito en diciembre 2019 en Wuhan – China; representa un problema de salud público

importante por su rápida propagación y la capacidad de generar neumonía grave en pacientes susceptibles; al momento no se a descrito ningún tratamiento específico para el virus siendo su manejo principalmente el soporte.

Antecedentes El brote de infección respiratoria por el coronavirus 2019 (2019-nCOV) inició en China en diciembre 2019, en los últimos días el brote se ha ido extendiendo a otros países llegando a declararse el riesgo de una epidemia internacional. Hasta el momento no se cuenta con un tratamiento efectivo disponible para el COVID - 19, sin embargo, in vitro se reportó la efectividad de la cloroquina en concentraciones capaces de ser obtenidas in vivo en pacientes que la usan. La cloroquina e hidroxicloroquina, son medicamentos originalmente antimaláricos, en uso regular por décadas y con una toxicidad limitada, a un costo muy bajo, y en caso de ser efectivo in vivo, podría ser un medicamento de gran utilidad tanto en países desarrollados como en desarrollo. El efecto antiviral amplio, se ha atribuido al incremento del pH de los endosomas, que se requieren para la fusión del virus con la célula y también por interferencia con la glicosilación de los receptores celulares del SARS-COV.

La tasa de letalidad general de la infección por COVID - 19 se ha estimado alrededor del 2%, pero en los casos graves en falla respiratoria, o requiriendo cuidados críticos, es considerablemente mayor, entre 10-15%, y fácilmente podría incrementarse hasta el 20-30% dependiendo de los criterios de entrada a una unidad de terapia intensiva y la disponibilidad de unidades especializadas.

Justificación Se carece de un tratamiento farmacológico aprobado para el COVID - 19, y basados en la efectividad in Vitro de la cloroquina, su seguridad demostrada por décadas, su costo bajo, se propone un ECC, doble ciego, para evaluar la eficacia y seguridad de la hidroxicloroquina en enfermos hospitalizados con enfermedad respiratoria grave por COVID - 19.

Hipótesis El tratamiento con hidroxicloroquina 400 mg/día por 10 días comparado con placebo reduce mortalidad intrahospitalaria en sujetos con enfermedad respiratoria grave por COVID – 19.

Objetivo primario y secundario Estimar si el tratamiento por 10 días con 400 mg/día de hidroxicloroquina comparada con placebo reduce mortalidad intrahospitalaria, medida a los 30 dias. Los desenlaces secundarios serán días de ventilación mecánica,

requerimiento de soporte ventilatorio mecánico, días de hospitalización y la incidencia acumulada de eventos adversos serios y eventos adversos grado 3 y 4.

Metodología Ensayo clínico aleatorizado doble ciego. Se incluirá a sujetos mayores de 18 años con diagnóstico de enfermedad grave de COVID-19 confirmada por RT-PCR y enfermedad grave que acepten participar. La aleatorización será realizada en software dedicado por personal no involucrado en el tratamiento, diseño ni análisis del estudio. Se estratificarán a los sujetos en dos grupos (sujetos que requieran hospitalización y sujetos que requieran soporte ventilatorio al momento de la aleatorización). Se realizará el análisis en STATA y en software estadístico R-Rstudio. Se compararán los desenlaces principales mediante pruebas paramétricas y no paramétricas de acuerdo al tipo de variable y distribución. Se Calcula el tamaño de muestra de acuerdo a la mortalidad en la población con enfermedad grave por COVID – 19 estimándose una muestra de 500 sujetos.

Personal del estudio y responsabilidades

Nombre	Afiliación	Institución	Responsabilidades
Dra Carmen Hernandez Cárdenas	Unidad de Cuidados Intensivos Respiratorios	INER	Investigador Principal
Dr. Cristobal Guadarrama	Unidad de Urgencias Respiratorias	INER	Subinvestigador
Dr. Rogelio Perez Padilla	Departamento de Investigación en Tabaquismo y EPOC	INER	Subinvestigador
Dr. Joel Armando Vasquez	Departamento de Investigación en Tabaquismo y EPOC	INER	Subinvestigador
Dra Ireri Isadora Thirion Romero	Departamento de Investigación en Tabaquismo y EPOC	INER	Subinvestigador
Dr. Luis Felipe Jurado Camacho	Unidad de Cuidados Intensivos Respiratorios	INER	Subinvestigador
Dra Arantxa	Departamento de Investigación en Tabaquismo y EPOC	INER	Coordinadora Institucional

Cronograma de evaluaciones

	CRIBADO	TIEMPO BASAL	DIARIO HASTA DÍA 10	DIARIO HASTA ALTA HOSPITALARIA
**VENTANA EN DÍAS +/-**	-1 o 1	1		
**CRIBADO**				
Elegibilidad	X			
Verificar resultado de sars-cov-2	X			
Consentimiento informado	X			
Toma de mediciones basales		X		
**INTERVENCION**				
Aleatorización		x		
Administracion de hidroxicloroquina/placebo		Diario hasta el día 10		
Procedimientos del estudio				
Registro de po2 y fio2				Diario hasta el alta hospitalaria

Reclutamiento y plan de retención

Reclutamiento

- Se anticipa que los sujetos con COVID-19 se presentarán a los centros participantes; por tanto, no se realizarán otros esfuerzos para reclutar a los sujetos necesarios.
- Se diseminará información acerca del ensayo a otros centros y profesionales médicos.
- Se evaluará para elegibilidad a todos los sujetos que ingresen a urgencias con sospecha de enfermedad grave por COVID-19; se aleatorizará e iniciará la intervención sólo tras la firma del consentimiento informado.

Criterios de inclusión

1. Firma de Consentimiento informado por el sujeto (o representante legal) entendiendo y aceptando los procedimientos y medicación del estudio descritos a continuación.
    a. Aleatorización a hidroxocloroquina o placebo idéntico.

    b. Recolección de muestra orofaringea y muestras sanguíneas para confirmación y seguimiento de COVID – 19.
    c. Hospitalización o ingreso a unidad de cuidados intensivos.
2. En caso de ser mujer prueba de embarazo negativa.
3. Al menos 18 años cumplidos al momento de la aleatorización.
4. Confirmación por laboratorio de infección por SARS-CoV-2 determinada por RT-PCR de muestra faríngea, nasofaríngea, lavado broncoalveolar o aspirado traqueal antes de la aleatorización.
5. Enfermedad grave por COVID – 19 definida por al menos uno de los siguientes:
    a. SpO2 menor o igual a 90 % a una altura de 2240 m, en el valle de México o Puebla o disminución de más del 3% de saturación basal
    b. Requiere ventilación mecánica y/o oxígeno suplementario (o incremento del apoyo de oxigeno suplementario en hipoxemia crónica)
    c. Cumple criterios de sepsis/choque séptico.

Criterios de exclusión:

1. Mayor de 80 años.
2. Hipersensibilidad nueva o conocida a hidroxicloroquina manifestada por anafilaxia.
3. Consumo previo a la hospitalización de hidroxicloroquina o cloroquina
4. Decisión del médico responsable de eliminarlo del estudio.
5. Transferencia del paciente a otra unidad hospitalaria.
6. Antecedente de enfermedad hepática crónica. (Child-Pugh B o C)
7. Antecedente de enfermedad renal crónica. (TFG menor o igual a 30)

Criterios de retiro/eliminación:

1. Decisión del médico responsable de retirar al participante del estudio.
2. Transferencia del paciente a otra unidad hospitalaria.
3. Hipersensibilidad nueva a hidroxicloroquina manifestada por anafilaxia.
4. Efecto adverso grado 3 o 4
5. Decisión del participante o representante de retirarse del estudio.

Plan de compensación para sujetos

El INER en su carácter de Patrocinador de la investigación vigilará el seguimiento de los participantes y la presencia de reacciones adversas; dando seguimiento clínico a los sujetos afectados.

Intervención del estudio

Los sujetos serán aleatorizados tras la firma del consentimiento informado a uno de los siguientes grupos:

- Grupo intervención: Hidroxicloroquina 200mg VO o por sonda nasogástrica/orogástrica cada 12 horas por 10 días. En pacientes que no toleren la vía oral se administrará la tableta tras el procedimiento estándar de administración por sonda.
- Grupo control: placebo idéntico, 1 tableta VO o por sonda nasogástrica/orogástrica cada 12 horas por 10 días. En pacientes que no toleren la vía oral se administrará la tableta tras el procedimiento estándar de administración por sonda.

Nota: Para administrar la tableta por sonda oro/nasogástrica se procederá a molerla en mortero, se mezcla con 10cc de agua destilada y se la administra con una jeringa; tras la administración se lavará la sonda orogástrica con 20 cc de agua destilada.

Aleatorización

Los sujetos serán aleatorizados tras la firma de consentimiento informado al grupo intervención o placebo; el médico tratante se comunicará vía telefónica al COORDINADOR INSTITUCIONAL quien registrará el estrato correspondiente e indicará el número de sobre que contiene la medicación par administración subsecuente. Sólo el COORDINADOR INSTITUCIONAL tendrá la lista de aleatorización con los códigos de los sujetos.

La aleatorización será estratificada por:

1. Centro reclutador
2. Gravedad de la enfermedad:

a. COVID-19 crítica: requiere ventilación mecánica invasiva o no invasiva.
b. COVID-19 grave con necesidad de hospitalización: SpO2 menor a 90% a aire ambiente o caída de 3% de la SpO2 basal o incremento de los requerimientos de oxígeno, taquipnea (>25 rpm)

Cegado y fin del cegado

El grupo al que pertenece el sujeto será cegado a los médicos y personal de enfermería tratante y al analista de datos.

Se finalizará el cegado en caso de que el médico tratante indique la necesidad de retirar del estudio o se cumplan otros criterios de retiro/eliminación.

Reporte de seguridad

Riesgos potenciales conocidos

Los riesgos potenciales asociados en este estudio incluyen posibles reacciones adversas a hidroxicloroquina y fallas en el mantenimiento de la confidencialidad.

Riesgos potenciales de la hidroxicloroquina

La hidroxicloroquina es un agente terapéutico conocido y relativamente seguro; algunos sujetos pueden experimentar molestias abdominales manifestadas por nausea, vómitos y/o diarrea; efectos leves de resolución espontánea al terminar el tratamiento o con medidas sintomática.

En algunos casos se encuentra retinopatía progresiva; (frecuencia de 1% al 10% tras cinco años de consumo y con dosis mayores a 6mg/Kg/día); se limita la frecuencia de esta complicación al no superar una dosis de 400mg/día y el tratamiento por 10 días. Se produce debido a la unión a melanina y depósito en la retina. Se monitorizará la presencia de reacciones adversas graves y grado 3 y 4 durante la duración del tratamiento y hasta el alta hospitalaria.

Riesgos a la privacidad

Los sujetos proveerán información personal relacionada con su estado de salud y antecedentes patológicos y no patológicos; se intentará mantener esta información confidencial dentro de los límites de la ley; de todas formas, existe una pequeña probabilidad de que personas no autorizadas vean los datos del sujeto. Todos los archivos y evaluaciones serán almacenadas en un gabinete bajo llave y dentro una

oficina cerrada con llave en el centro. Los archivos electrónicos serán protegidos mediante password.

Solo las personas involucradas en la planificación, supervisión, monitoreo o auditorias del estudio tendrán acceso a la información colectada.

Toda publicación de este ensayo utilizará información de-identificada; organizaciones que podrían inspeccionar y/o copiar la información de la investigación para evaluación de la calidad y el análisis de datos se limitan a grupos como el Comité de ética e investigación institucional, sponsor y otras autoridades regulatorias pertinentes.

Retención

Al incluir en el estudio sólo sujetos que requieran hospitalización no se esperan pérdidas más que cuando se solicite alta por parte de los familiares o cumplan criterios de retiro/eliminación.

Colección de datos y forma de reporte de caso

Instrucciones generales para completar las formas de reporte de caso

- La Forma de Reporte de Caso debe ser completada para todos los participantes que acepten y firmen el consentimiento informado.
- A excepción de la hoja correspondiente a “INFORMACION DE CONTACTO” ninguna hoja de la Forma de Reporte de Caso debe llevar el nombre ni otros datos que identifiquen al participante.
- La hoja correspondiente a “INFORMACION DE CONTACTO” debe ser resguardada bajo llave por separado del resto de la forma.
- Llenar todas las entradas con mayúsculas.
- Todo el texto y comentarios deben ser breves; en caso de que falte espacio se puede usar el reverso de la hoja; todo comentario debe ir fechado y con nombre y firma del responsable.
- Responder todas las preguntas explícitamente.
- No dejar preguntas sin responder. Si la respuesta no se conoce escribir “D”(Desconoce). Si no se realizó “NR” (No se realizó). Si la pregunta no es aplicable escribir “NA” (No aplicable).
- Si se requiere una selección tachar con X.

- Todas las entradas de fecha deben aparecer en el formato DD—MMM-AAAA, en caso de que se desconozca la fecha exacta de un evento debe anotarse DD.
- Las horas deben llenarse en un formato de 24-horas; las entradas de media noche se anotan 00:00.
- En caso de errores:
  - No escriba encima, no use líquido corrector ni borradores.
  - Tache el dato erróneo sin obliterarlo.
  - Claramente corrija el dato erróneo al lado de la entrada incorrecta.
  - Coloque la fecha y las iniciales de la persona que hace la corrección.

Instrucciones generales para completar la base de datos

- La base de datos será llenada en Redcap o en un programa de base de datos; deben transcribirse los datos tal y como están en la forma de recolección de datos.
- Fechas en formato mes/día/año.
- No usar mayúsculas ni espacios; los espacios deben ser remplazados por _.
- En caso de no contar con algún dato llenar con NA.

Almacenamiento de documentos de estudio

Las CRF serán almacenadas en el centro coordinador bajo llave; con acceso sólo al coordinador del centro e investigador principal hasta la conclusión del estudio.

Manejo de datos

Procedimientos de control de calidad

Los datos serán transcritos de los CRF a la base de datos y cuando completa y limpia se transferirá a STATA o al programa de análisis; tendrá mensajes de validación y errores de transcripción activados.

Se verificará aleatoriamente el 20% de las CRF con el expediente de cada sujeto buscando errores en el llenado de las CRF, se reportará el porcentaje de CRF con errores.

Actividades de monitoreo de seguridad de datos y seguridad

Procedimientos de confidencialidad

La base de datos será llenada con un código por sujeto; se eliminarán datos que identifiquen a los sujetos como numero de expediente, nombre y fecha de ingreso a la institución.

Seguridad

EL equipo del protocolo revisará de forma cegada datos de eventos adversos grado 3 o 4 y eventos adversos graves cada día por 10 días y el momento del alta hospitalaria. Si se encuentra un numero preocupante de eventos adversos inesperados o las muertes están disparejas en los grupos aleatorizados, se solicitará al Comité de Ética e Investigación que revise los datos sin cegamiento en una reunión ad hoc.

El Comité de Ética e Investigación solicitará información o reuniones ad hoc extra si existen otras preocupaciones acerca de la seguridad. El estudio no parará el reclutamiento esperando las revisiones del Comité de Ética e Investigación, aunque el DSMB puede recomendar suspensión temporal o permanente del reclutamiento en base a las revisiones de seguridad.

Operacionalización de variables

Variable	Periodo	Definición	Código
**Criterios de inclusión**	Aleatorización	Características que se deben tener para participar en el estudio.	1: Cumple todos<br>2: No los cumple
**Fecha de ingreso a urgencias**	Aleatorización	Fecha de hospitalización a urgencias	Fecha (Dd/Mm/Aa)<br>Hora (Hh:Mm)
**Edad**	Aleatorización	Edad en años cumplidos	Años
**Genero**	Aleatorización	Género reportado por sujeto o familiares	1: Masculino<br>0: Femenino
**Fecha de inclusión**	Aleatorización	Fecha de reclutamiento a protocolo	Fecha (Dd/Mm/Aa)
**Días de inicio de sintomatología**	Aleatorización	Días cumplidos desde el inicio de síntomas reportado por el sujeto o familiares.	1, 2, 3, etc…
**Comorbilidades**	Aleatorización	Presencia de comorbilidades reportada por sujeto o familiares.<br><br>Se reportan hasta tres comorbilidades.	0: ninguna<br>1: Respiratoria crónica<br>2: Cardiovascular<br>3: Renal<br>4: Metabólica<br>5: otras (anotar)
**Ventilación no**	Aleatorización	Uso de asistencia ventilatoria	0: No

**invasiva**		no invasiva.	1: Si (BiPAP/CPAP)<br>2: Si (Puntas de alto flujo)
**Fecha de inicio de VNI**	Aleatorización	Fecha de inicio de ventilación no invasiva	Fecha (Dd/Mm/Aa)
**Ventilación invasiva**	Aleatorización	Uso de asistencia ventilatoria invasiva	0: No<br>1: Si
**Fecha de intubación orotraqueal**	Aleatorización	Fecha en que se realizó la intubación orotraqueal	Fecha (Dd/Mm/Aa)
**Uso de aminas**	Aleatorización	Uso de aminas vasoactivas (dopamina, norepinefrina o adrenalina)	0: No<br>1: Si
**Lactato**	-Aleatorización<br>-Seguimiento día 2 a día 10.<br>-Día de conclusión (alta o fallecimiento).	Forma ionizada del lactato. Un intermediario normal en la fermentación (oxidación, metabolismo) del carbohidratos.	mmol/L
**PaO2**	-Aleatorización<br>-Seguimiento día 2 a día 10.<br>-Día de conclusión (alta o fallecimiento).	Presión arterial de oxígeno	mmHg
**FiO2**	-Aleatorización<br>-Seguimiento día 2 a día 10.<br>-Día de conclusión (alta o fallecimiento).	Fracción inspiratoria de oxígeno	10^9/L
**Pco2**	-Aleatorización<br>-Seguimiento día 2 a día 10.<br>-Día de conclusión (alta o fallecimiento).	Presión parcial de CO2	mmHg
**Ph**	-Aleatorización<br>-Seguimiento día 2 a día 10.<br>-Día de conclusión (alta o fallecimiento).	Concentración de hidrogeniones	Unidades

**Leucocitos**	-Aleatorización -Seguimiento día 2 a día 10. -Día de conclusión (alta o fallecimiento).	Células nucleadas en sangre	$10^3/mm^3$
**Plaquetas**	-Aleatorización -Seguimiento día 2 a día 10. -Día de conclusión (alta o fallecimiento).	Las células en forma de disco no nucleadas se formaron en el megacariocito y se encuentran en la sangre de todos los mamíferos. Están involucrados principalmente en la coagulación de la sangre.	$10^9/L$
**Creatinina**	-Aleatorización -Seguimiento día 2 a día 10. -Día de conclusión (alta o fallecimiento).	Producto resultante del catabolismo muscular.	mg/dL
**Potasio**	-Aleatorización -Seguimiento día 2 a día 10. -Día de conclusión (alta o fallecimiento).	Catión principal en el líquido intracelular del músculo y otras células.	mEq/L
**Neutrofilos**	-Aleatorización -Seguimiento día 2 a día 10. -Día de conclusión (alta o fallecimiento).	Leucocitos granulares que tienen un núcleo con tres a cinco lóbulos conectados por hilos delgados de cromatina, y citoplasma que contiene gránulos finos discretos y se puede teñir con tintes neutros.	$/10^9/L$
**Eosinofilos**	-Aleatorización -Seguimiento día 2 a día 10. -Día de conclusión (alta o fallecimiento).	Leucocitos granulares con un núcleo que generalmente tiene dos lóbulos conectados por un hilo delgado de cromatina y citoplasma que contiene gránulos gruesos y redondos de tamaño uniforme y manchables por la eosina.	$10^9/L$
**BUN**	-Aleatorización -Seguimiento día 2 a día 10. -Día de	Producto final del catabolismo proteico.	mg/dL

	conclusión (alta o fallecimiento).		
**TP**	-Aleatorización<br>-Seguimiento día 2 a día 10.<br>-Día de conclusión (alta o fallecimiento).	Determinación del tiempo de coagulación del plasma citratado, tras la adición de un exceso de tromboplastina tisular y calcio. Sirve para medir la vía extrínseca de la coagulación, así como la vía común	S
**INR**	-Aleatorización<br>-Seguimiento día 2 a día 10.<br>-Día de conclusión (alta o fallecimiento).	Sistema establecido por la Organización Mundial de la Salud y Comité internacional en trombosis y hemostasia para monitorear e informar las pruebas de coagulación sanguínea. Bajo este sistema, los resultados se estandarizan usando el Índice de sensibilidad internacional para la combinación particular de reactivo de prueba / instrumento utilizado.	Unidades
**TTPA**	-Aleatorización<br>-Seguimiento día 2 a día 10.<br>-Día de conclusión (alta o fallecimiento).	Consiste en medir el tiempo de coagulación del plasma citratado, en contacto con calcio y fosfolípidos (cefalina). Mide la vía intrínseca de la coagulación y la vía común.	segundos
**Sodio**	-Aleatorización<br>-Seguimiento día 2 a día 10.<br>-Día de conclusión (alta o fallecimiento).	Determinante primario de la osmolaridad plasmática y contribuye a la regulación del volumen extracelular	mEq/L
**Bilirrubina directa**	-Aleatorización<br>-Seguimiento día 2 a día 10.<br>-Día de conclusión (alta o fallecimiento).	Bilirrubina conjugada por el hígado, está unida a ácido glucorónico.	mg/dL
**Bilirrubina total**	-Aleatorización	Suma de bilirrubina indirecta y	mg/dL

	-Seguimiento día 2 a día 10.<br>-Día de conclusión (alta o fallecimiento).	directa.	
**AST**	-Aleatorización<br>-Seguimiento día 2 a día 10.<br>-Día de conclusión (alta o fallecimiento).	Miembro de un grupo de enzimas que catalizan la transferencia de un grupo amino de un aminoácido a un cetoácido, dando lugar a la formación de un nuevo aminoácido y dejando como residuo un nuevo tipo de cetoácido. AST o TGO.	UI/L
**ALT**	-Aleatorización<br>-Seguimiento día 2 a día 10.<br>-Día de conclusión (alta o fallecimiento).	Son enzimas que transforman proteínas en ATP para las células hepáticas. Alaninoaminotransferasa o transaminasa glutámico-pirúvica (ALT TGP)	UI/L
**Volumen tidal espiratorio**	-Aleatorización<br>-Seguimiento día 2 a día 10.<br>-Día de conclusión (alta o fallecimiento).	Volumen espiratorio que queda al final del volumen corriente.	ml
**PEEP**	-Aleatorización<br>-Seguimiento día 2 a día 10.<br>-Día de conclusión (alta o fallecimiento).	Presión supratmosferica aplicada por el ventilador al final de la espiración	cmH2O
**Frecuencia respiratoria**	-Aleatorización<br>-Seguimiento día 2 a día 10.<br>-Día de conclusión (alta o fallecimiento).	Número de respiraciones durante un periodo de tiempo	Rpm
**Presión meseta**	-Aleatorización<br>-Seguimiento día 2 a día 10.<br>-Día de	Presión a los dos segundos de una maniobra de pausa inspiratoria. Verificar flujo cero.	cmH2O

	conclusión (alta o fallecimiento).		
**Presión media**	-Aleatorización<br>-Seguimiento día 2 a día 10.<br>-Día de conclusión (alta o fallecimiento).	Presión promedio durante un ciclo respiratorio completo.	CmH2O
**Efectos adversos**	-Aleatorización<br>-Seguimiento día 2 a día 10.<br>-Día de conclusión (alta o fallecimiento).	Cualquier otra respuesta del fármaco que es nociva, no intencionada y que se produce a dosis habituales para la profilaxis, diagnóstico o tratamiento.	0: No<br>1: Si
**Efectos adversos visuales**	-Aleatorización<br>-Seguimiento día 2 a día 10.<br>-Día de conclusión (alta o fallecimiento).	Cualquier respuesta del sistema ocular al fármaco que es nociva, no intencionada y que se produce a dosis habituales para la profilaxis, diagnóstico o tratamiento.	0: No<br>1: Si<br>Pérdida de visión<br>Otro____
**Efectos adversos cardiovasculares**	-Aleatorización<br>-Seguimiento día 2 a día 10.<br>-Día de conclusión (alta o fallecimiento).	Cualquier respuesta del sistema cardiovascular al fármaco que es nociva, no intencionada y que se produce a dosis habituales para la profilaxis, diagnóstico o tratamiento.	0: No<br>1: Si<br>Hipotensión<br>QTc prolongado<br>Depresión onda T<br>Otro___
**Efectos adversos neurológicos**	-Aleatorización<br>-Seguimiento día 2 a día 10.<br>-Día de conclusión (alta o fallecimiento).	Cualquier respuesta del sistema neurológico al fármaco que es nociva, no intencionada y que se produce a dosis habituales para la profilaxis, diagnóstico o tratamiento.	0: No<br>1: Si<br>Confusión<br>Mareo<br>Cefalea<br>Parestesias<br>Convulsiones<br>Neuropatía<br>Otros____
**Efectos adversos hepatobiliar**	-Aleatorización<br>-Seguimiento día 2 a día 10.<br>-Día de conclusión (alta o fallecimiento).	Cualquier respuesta del sistema hepatobiliar al fármaco que es nociva, no intencionada y que se produce a dosis habituales para la profilaxis, diagnóstico o tratamiento.	0: No<br>1: Si<br>Elevación de transaminasas

**Efectos adversos gastrointestinales**	-Aleatorización<br>-Seguimiento día 2 a día 10.<br>-Día de conclusión (alta o fallecimiento).	Cualquier respuesta del sistema gastrointestinal al fármaco que es nociva, no intencionada y que se produce a dosis habituales para la profilaxis, diagnóstico o tratamiento.	0: No<br>1: Si<br>Vomito/nausea<br>Diarrea<br>Otro____
**Efectos adversos dermatológicos**	-Aleatorización<br>-Seguimiento día 2 a día 10.<br>-Día de conclusión (alta o fallecimiento).	Cualquier respuesta dermatológica al fármaco que es nociva, no intencionada y que se produce a dosis habituales para la profilaxis, diagnóstico o tratamiento.	0: No<br>1: Si<br>Prurito<br>Alopecia<br>Eritema/urticaria<br>Otro___
**Efectos adversos hematológicos**	-Aleatorización<br>-Seguimiento día 2 a día 10.<br>-Día de conclusión (alta o fallecimiento).	Cualquier respuesta hematológica al fármaco que es nociva, no intencionada y que se produce a dosis habituales para la profilaxis, diagnóstico o tratamiento.	0: No<br>1: Si<br>Agranulocitosis/trombocitopenia<br>Pancitopenia<br>Otro_____
**Efectos adversos auditivos**	-Aleatorización<br>-Seguimiento día 2 a día 10.<br>-Día de conclusión (alta o fallecimiento).	Cualquier respuesta del sistema auditivo al fármaco que es nociva, no intencionada y que se produce a dosis habituales para la profilaxis, diagnóstico o tratamiento.	0: No<br>1: Si<br>Tinitus<br>Pérdida de audición<br>Otro____
**Efectos adversos otros**	-Aleatorización<br>-Seguimiento día 2 a día 10.<br>-Día de conclusión (alta o fallecimiento).	Cualquier otra respuesta del fármaco que es nociva, no intencionada y que se produce a dosis habituales para la profilaxis, diagnóstico o tratamiento.	0: No<br>1: Si<br>_________
**Detección de reacciones adversas grado 3 o 4**	-Aleatorización<br>-Seguimiento día 2 a día 10.<br>-Día de conclusión (alta o fallecimiento).	Detecciones de efectos adversos grado 3 o 4.	0: No<br>1: Si
**Reacciones adversas Grado 3 (Grave)**	-Aleatorización<br>-Seguimiento día 2 a día 10.<br>-Día de conclusión	El resultado para el paciente es sintomático y exige una intervención quirúrgica o médica mayor, acorta la esperanza de vida o causa un	0: No<br>1: Si

	(alta o fallecimiento).	daño o una pérdida funcional importante y permanente o de largo duración.<br>Especificar_______	
**Reacciones adversas Grado 4 (Muerte)**	-Aleatorización<br>-Seguimiento día 2 a día 10.<br>-Día de conclusión (alta o fallecimiento).	Sopesando las probabilidades, el incidente causó la muerte o la propicio a corto plazo<br>Especificar___________	0: No<br>1: Si
**Administración fármaco**	Desenlace	Se administró el medicamento durante 10 días	0: Si<br>1: No
**Fecha de alta hospitalaria o fallecimiento**	Desenlace	Si fallece o se va de alta, fecha en la que ocurre el evento	0: Fallece<br>1: Alta<br>Fecha (Dd/Mm/Aa)
**Días totales de hospitalización**	Desenlace	Días cumplidos desde el inicio desde la aleatorización hasta el alta hospitalaria o fallecimiento	1, 2, 3, etc...
**Días totales de asistencia ventilatoria mecánica invasiva**	Desenlace	Días cumplidos desde el inicio desde la aleatorización hasta el alta hospitalaria o fallecimiento	1, 2, 3, etc.
**Ventilación no invasiva**	Desenlace	Uso de asistencia ventilatoria no invasiva.	0: No<br>1: Si (BiPAP/CPAP)<br>2: Si (Puntas de alto flujo)
**Fecha de inicio de ventilación no invasiva**		Fecha en la que se inició el uso de asistencia ventilatoria no invasiva	Fecha (Dd/Mm/Aa)
**Fecha de término de ventilación no invasiva**		Fecha en la que se suspendió el uso de asistencia ventilatoria no invasiva	Fecha (Dd/Mm/Aa)
**Ventilación invasiva**	Desenlace	Uso de asistencia ventilatoria invasiva	0: No<br>1: Si
**Fecha de inicio de ventilación invasiva**	-	Fecha en la que se inició el uso de asistencia ventilatoria invasiva	Fecha (Dd/Mm/Aa)
**Fecha de término de ventilación invasiva**	-	Fecha en la que se suspendió el uso de asistencia ventilatoria invasiva<br>Desconexión de AVM si traqueostomizado	Fecha (Dd/Mm/Aa)

Plan de análisis de datos

El análisis estadístico se realizará software estadístico STATA o R-R studio utilizando la última actualización disponible al momento del análisis de datos. Será realizado de forma cegada al tratamiento recibido por los individuos y tras de-identificación de los datos.

Tamaño de muestra

A) **TAMAÑO DE MUESTRA**

La mortalidad reportada en el COVID-19 grave se encuentra entre el 8% a incluso al 60% dependiendo la fuente y la OMS sugiere al menos 400 sujetos para la estimación de la muestra en ensayos clínicos que evalúen terapias para el COVD-19 (3). Al momento se desconocen muchas características del comportamiento y desenlaces del COVID-19 especialmente en nuestro medio; por eso utilizamos la estimación de una mortalidad alrededor del 15% en COVID-19 grave con una reducción del 50% de la mortalidad con el tratamiento obteniendo en el cálculo de muestra un número de sujetos similar al recomendado por la OMS.

Nivel de significancia de 2 colas 95, alfa 5%

Potencia (1-beta, posibilidad de detección): 80%

Tratados vs controles 1:1

Mortalidad estimada en controles 15%

Mortalidad estimada en experimentales 7.5%, reducción de mortalidad de 7.5%, que es el 50% (RR 0.5).

<u>Estimaciones (Open-Epi)</u>

	Kelsey		Fleiss	Fleiss with CC
Muestra experimental		280	279	305
Muestra control		280	279	305
Muestra total :	560	558	610	

Se decide incluir 600 sujetos para el ensayo clínico, es decir 300 sujetos en el grupo de tratamiento experimental y 300 sujetos en el grupo control.

Estadística descriptiva y bivariada.

Se utilizará estadística descriptiva de acuerdo al tipo de variable y distribución. Se comparará la mortalidad entre el grupo placebo e intervención mediante prueba exacta de fisher. Se compararán tiempos a los desenlaces y supervivencia global y por grupo ajustando por variables confusoras (edad, tiempo del inicio de síntomas al inicio de tratamiento, comorbilidades) mediante modelo de riesgos proporcionales de cox.

Análisis:

- La tasa de mortalidad (30 días), la proporción de pacientes que necesitan ventilación mecánica y los días de ventilación mecánica, se compararán en un

modelo multivariado, que incluye un indicador de estratos de aleatorización, ajustando las variables de confusión (edad, tiempo desde el inicio de los síntomas hasta el inicio de tratamiento, comorbilidades), se realizará una comparación similar para los resultados secundarios: duración de la hospitalización, duración de la oxigenoterapia.

- También compararemos el tiempo hasta la muerte, en forma cruda y ajustando las variables de confusión (edad, tiempo desde el inicio de los síntomas hasta el inicio del tratamiento, comorbilidades) utilizando el modelo de riesgos proporcionales de Cox. El mismo modelo se utilizará para estimar el tiempo a egreso y el tiempo a mejoría.
-
-
- Habrá un Comité de eventos adversos y evaluación de riesgos y análisis intermedio, un grupo asesor multidisciplinario, compuesto por un experto en estadística, un investigador clínico, un experto en ética y un clínico relacionado con la influenza y el coronavirus, ninguno involucrado en el ensayo clínico y, por lo tanto, independiente, que en el medio de los ensayos tendrá acceso a los datos y al código de aleatorización para evaluar los datos, incluida la seguridad y los resultados.

Investigador clínico: Gustavo Lugo, farmacólogo clínico, anestesiólogo e intensivista que actuará como coordinador. Dra. Angélica Portillo, especialista en oído, nariz y garganta y con una maestría en ciencias médicas.

Estadísticas: Dra. Rosario Fernández, Jefa del Departamento de Epidemiología del INER.

Ética: Dr. Patricio Santillán, director médico de INER.

Clínico: Dr. Arturo Martínez, Infectólogo, experto en infecciones similares a la gripe y ahora en infección por COVID-19.

Instrucciones generales para el llenado de la forma:

Llenado de la Forma

- Esta Forma debe ser completada para todos los sujetos que autoricen su participación.
- Por razones de confidencialidad no debe aparecer en esta forma el nombre ni las iniciales del paciente.

General

- Por favor llenar todas las entradas con mayúsculas.
- Todo el texto y comentarios deben ser breves.
- Responder todas las preguntas explícitamente.
- No dejar preguntas sin responder. Si la respuesta no se conoce escribir **"D"(Desconocido).** SI no se realiza **"NR"(No realizado).** Si la pregunta no es aplicable escribir **"NA" (No aplicable).**
- Si se requiere una selección tachar con **X**

Fecha y Hora

- Todas las entradas de fecha deben aparecer en el formato DD-MMM-AAAA e.g. 05-May-2009. Las abreviaciones de meses son las siguientes:

Enero	=	Ene	Mayo	=	May	Septiembre	=	Sep
Febrero	=	Feb	Junio	=	Jun	Octubre	=	Oct
Marzo	=	Mar	Julio	=	Jul	Noviembre	=	Nov
Abril	=	Abr	Agosto	=	Ago	Diciembre	=	Dic

En caso de que se desconozca la fecha exacta de un evento debe anotarse **DD**

D D	E N E	2 0 0 9
DD	MMM	AAAA

- Las horas deben llenarse en un formato de **24-horas** e.g. 13:00. Las entradas de media noche se anotan 00:00

Errores

- **No escriba encima, no use liquido corrector ni borradores.**
- Tache el dato erróneo sin obliterarlo.
- Claramente corrija el dato erróneo al lado de la entrada incorrecta.
- Coloque la fecha y las iniciales de la persona que hace la corrección.

**INFORMACIÓN DEL PARTICIPANTE**		
Nombre del centro de estudio	INSTITUTO NACIONAL DE ENFERMEDADES RESPIRATORIAS<br>Ismael Cosío Villegas	
Criterios de inclusión:	☐ Mayor de 18 años<br>☐ Consentimiento informado firmado<br>☐ **Infección confirmada por COVID-19:** Por RT-PCR y muestra faríngea y nasofaríngea, o en lavado broncoalveolar o aspirado traqueal y/o infección pulmonar documentada por opacidades nuevas en placa de tórax o tomografía<br>☐ Inicio de síntomas menor a 14 dias<br>☐ Enfermedad grave por COVID – 19 definida por al menos uno de los siguientes:<br>☐ SpO2 menor o igual a 90 % a una altura de 2240 m, en el valle de México o Puebla o disminución de más del 3% de saturación basal.<br>☐ Requiere ventilación mecánica y/o oxígeno suplementario (o incremento del apoyo de oxigeno suplementario en hipoxemia crónica)<br>☐ Cumple criterios de sepsis/choque séptico.	
Criterios de exclusión	☐ Mayor de 80 años<br>☐ Prueba de Embarazo positiva.<br>☐ Alergia previa a la cloroquina, hidroxicloroquina o amodiaquina<br>☐ QTc>450/500mmsec hombre/mujer, ECG, Escala de riesgo de prolongacion QT, muy alto (>=11)<br>☐ **M**edicamento con interacción peligrosa con la hidroxicloroquina e incapacidad para evitarla<br>☐ Patología retiniana<br>☐ Deficiencia de G6PD conocida<br>☐ Historia de falla cardíaca, infarto miocárdico, arritmias<br>☐ Infección previa por COVID 2019<br>☐ Decisión del médico responsable de excluir del estudio.<br>☐ Transferencia del paciente a otra unidad hospitalaria<br>☐ Tratamiento actual o previo con hidroxicloroquina (un mes)<br>☐ Enfermedad renal o hepática crónica. (falla renal crónica con TFG menor a 30mL o ChildPugh B o C).	
Criterios de Inclusión:<br>*Debe cumplir todos	Cumple todos ☐1.	No los cumple ☐2.
Fecha de nacimiento	D D M M M A A A A	Edad (años)
Género	☐1 Masculino ☐0 Femenino	
Fecha Ingreso Urgencias	D D M M M A A A A	H H : M M
Fecha de inclusión (fecha de la aleatorización)	D D M M M A A A A	
Fecha de toma de PCR SARS COV-2	D D M M M A A A A	
Coinfección viral	☐0 Ninguna ☐1 SARS COV-2 ☐2 Influenza A<br>☐4 H1N1 ☐5 H1 ☐6 H3<br>☐7 H5 ☐8 H7N9 ☐9 Influenza B	

SINTOMAS BASALES			
Fecha inicio de síntomas	D D M M M Y Y Y Y		O ☐1 Desconocido
Anorexia	☐1. Si	☐2. No	Duración: _______ días
Artralgias	☐1. Si	☐2. No	Duración: _______ días
Cefalea	☐1. Si	☐2. No	Duración: _______ días
Conjuntivitis	☐1. Si	☐2. No	Duración: _______ días
Diarrea	☐1. Si	☐2. No	Duración: _______ días
Disnea	☐1. Si	☐2. No	Duración: _______ días
Escalofríos	☐1. Si	☐2. No	Duración: _______ días
Esputo	☐1. Si	☐2. No	Duración: _______ días
Fatiga	☐1. Si	☐2. No	Duración: _______ días
Fiebre	☐1. Si	☐2. No	Duración: _______ días
Malestar general	☐1. Si	☐2. No	Duración: _______ días
Nauseas	☐1. Si	☐2. No	Duración: _______ días
Neumonia	☐1. Si	☐2. No	Duración: _______ días
Odinofagia	☐1. Si	☐2. No	Duración: _______ días
Tos productiva	☐1. Si	☐2. No	Duración: _______ días
Tos seca	☐1. Si	☐2. No	Duración: _______ días
Vomito	☐1. Si	☐2. No	Duración: _______ días
Otro síntoma:	_____________________		Duración: _______ días

COMORBILIDADES

	¿El sujeto presenta comorbilidades? ☐$_0$No ☐$_1$Si Favor de especificar comorbilidad:				
**Cod**	**Título**		**Cod**	**Título**	
1	Enfermedad neurológica		11	Uso de corticosteroides inhalados	
2	Enfermedad Cardiovascular		12	Uso de inmunosupresores	
3	Hipertensión arterial		13	Diabetes mellitus	
3a	Uso de Inhibidores de ECA (captopril, enalapril, etc.)		14	VIH	
4	ASMA		15	Enfermedad renal crónica	
5	EPOC		16	Enfermedad hepática	
6	Enfermedad pulmonar intersticial		17	Obesidad	
7	Tuberculosis		18	Uso de Ibuprofeno (o AINEs)	
8	Enfermedad autoinmune		19	Lupus Eritematoso, Artritis Reumatoide o enfautoinmune Precisar_____________________	
9	Alcoholismo		20	Cancer precisar________________	
10	Uso de corticoesteroides orales				

**Antecedente de vacunación**	Influenza 2019-2020 ☐$_0$. No. ☐$_1$.Si BCG ☐$_0$. No. ☐$_1$.Si Neumococo ☐$_0$. No. ☐$_1$.Si

**Hábito tabáquico**		
Tabaquismo actual ☐$_0$. **No.** ☐$_1$.**Si**	N° cigarrillos al día: ___	N° de años fumando: ___
Tabaquismo previo ☐$_0$. **No.** ☐$_1$.**Si**	Edad de inicio	Edad de abandono
Uso de cigarro electrónico en el último mes ☐$_0$. **No.** ☐$_1$.**Si**	☐$_1$. JUUL. ☐$_2$.IQOS	
Antecedente de consumo de marihuana en el último mes	☐$_0$. No. ☐$_1$.Si	

ESTADO BASAL

Datos obtenidos el día de la aleatorización; si existen múltiples mediciones durante ese día se toma el peor valor obtenido.

**Oxigenoterapia puntas nasales**	☐0. No<br>☐1. Si	**Litros por minuto _____**	D D M M M A A A A **Fecha de inicio**
**Ventilación no invasiva** al momento de la aleatorización	☐0. No<br>☐1. Si (CPAP/BiPAP)	☐2.Si (Puntas de alto flujo)	D D M M M A A A A **Fecha de inicio**
**Ventilación invasiva** al momento de la aleatorización	☐0. No	☐1. Si (Llenar parámetros en la siguiente hoja)	D D M M M Y Y Y Y **Fecha de intubación orotraqueal**
**Uso de aminas** al momento de la aleatorización	☐0. No	☐1. Si	

Monitorización basal **Exámenes complementarios**

Si son multiples mediciones anotar las peores tomadas de 00:00am a 12:00–

Fecha D D M M M A A A A	Hora 24 HRS	Lactato (mmol/L)	pO2 (mmHg))	FiO_2(10^9/L)
pCO_2 (mmHg)	pH	Leucocitos(10^3/mm^3)	Plaquetas (10^3/mm^3	SO_2(%)
Creatinina (mg/dL)	Potasio (mEq/L)	Neutrofilos(10^3/mm^3	Eosinofilos(10^3/mm^3	Linfocitos (10^3/mm^3)
BUN (mg/dL)	TP (s)	TTPA (s)	INR	
Sodio (mEq/L)	Bilirrubina directa (mg/dL)	Bilirrubina total(mg/dL)	TGO (UI/L)	TGP(UI/L)
BNP	Dímero D	PCR	Ferritina	
Signos vitales				
TA Sistólica (mmHg)	TA Diastólica (mmHg)	FC (lpm)	FR (Rpm)	Temperatura °C
Peso	Talla		I.M.C.	
Escala de Glasgow<br>☐0 **15Puntos** ☐1 13-14**Puntos**<br>☐2 10-12**Puntos** ☐3 6-9**Puntos**<br>☐4 <6**Puntos**	Flujo urinario<br>☐0 >500 ml/dl<br>☐1 <500 ml/dl<br>☐2 <200 ml/dl	Cardiovascular/aminas<br>☐0 PAM<70 mmHg<br>☐1 Dopamina a <5 o Dubatamina cualquier dosis<br>☐2 Dopamina 5.1-15 o Epi o Norepinefrina <0.1<br>☐3 Dopamina >15 o Epi o Norepinefrina >0.1		

Se encuentra con ventilación mecánica no invasiva?:
☐0. No. ☐1.Si (CPAP/BiPAP) ☐2.Si(Puntas de alto flujo)

Se encuentra con ventilación mecánica invasiva?: ☐0. No. ☐1. Si
En caso de encontrarse en ventilación mecánica invasiva llenar los siguientes parámetros

FiO_2 (los valores deben ser del 0.21 al 1)	Volumen tidal espiratorio mL	PEEP cmH_2O	Frecuencia respiratoria rpm	Presión meseta cmH_2O

Presión media cmH_2O	

Monitorización diaria Exámenes complementarios

Si son multiples mediciones anotar las peores tomadas de 00:00am a 12:00–

Fecha D D M M M A A A A	Hora 24 HRS	Lactato (mmol/L)	pO2 (mmHg))	$FiO_2(10^9/L)$
pCO_2 (mmHg)	pH	Leucocitos$(10^3/mm^3)$	Plaquetas $(10^3/mm^3$	SO_2(%)
Creatinina (mg/dL)	Potasio (mEq/L)	Neutrofilos$(10^3/mm^3$	Eosinofilos$(10^3/mm^3$	Linfocitos $(10^3/mm^3)$
BUN (mg/dL)	TP (s)	TTPA (s)	INR	
Sodio (mEq/L)	Bilirrubina directa (mg/dL)	Bilirrubina total(mg/dL)	TGO (UI/L)	TGP(UI/L)
BNP	Dímero D	PCR	Ferritina	

Signos vitales

TA Sistólica (mmHg)	TA Diastólica (mmHg)	FC (lpm)	FR (Rpm)	Temperatura °C	IMC

Escala de Glasgow<br>☐0 **15Puntos** ☐1 13-14**Puntos**<br>☐2 10-12**Puntos** ☐3 6-9**Puntos**<br>☐4 <6**Puntos**	Flujo urinario<br>☐0 >500 ml/dl<br>☐1 <500 ml/dl<br>☐2 <200 ml/dl	Cardiovascular/aminas<br>☐0 PAM<70 mmHg<br>☐1 Dopamina a <5 o Dubatamina cualquier dosis<br>☐2 Dopamina 5.1-15 o Epi o Norepinefrina <0.1<br>☐3 Dopamina >15 o Epi o Norepinefrina >0.1

Se encuentra con ventilación mecánica no invasiva: ☐0. No. ☐1.Si (CPAP/BiPAP) ☐2.Si(Puntas de alto flujo)

Se encuentra con ventilación mecánica invasiva:☐0. No. ☐1. Si

En caso de encontrarse en ventilación mecánica invasiva llenar los siguientes parámetros

FiO_2 (los valores deben ser del 0.21 al 1)	Presión media (cmH2O)	Volumen tidal espiratorio (ml)	PEEP(cmH2O)	FR (rpm)	Presión meseta (cmH2O)

¿Presenta alguna reacción adversa?☐0. No. ☐1. Si

Visuales	***Neurológicas***	***Hepatobiliar***	***Dermatológicas***	***Auditiva***
☐0. No. ☐1. Si:<br>1.Pérdida de visión------☐<br>2.Otro_______________☐<br>***Cardiovasculares***<br>☐0. No. ☐1. Si<br>1.Hipotensión----------☐<br>2.QTc prolongado----☐<br>3.Depresión onda T-☐<br>4.Otro_______________☐	☐0. No. ☐1. Si<br>1.Agitación/Confusión<br>☐<br>2.Mareo---------☐<br>3.Cefalea-------☐<br>4.Parestesias---☐<br>5.Convulsiones-☐<br>6.Neuropatía---☐<br>7.Otro____________☐	☐0. No. ☐1. Si:<br>1.Elevación de transaminasas-☐<br>2.Otros ☐<br>______________<br>***Gastrointestinales***<br>☐0. No. ☐1.Si:<br>1.Vomito☐<br>2.Nausea☐<br>3.Diarrea----☐<br>4.Otro________________<br>_______☐	☐0. No. ☐1. Si:<br>1.Prurito------------☐<br>2.Alopecia--------- -☐<br>3.Eritema/urticaria-☐<br>4.Otro___________☐<br>***Hematológicas***<br>☐0. No. ☐1. Si<br>1.Agranulocitosis☐<br>2.Trombocitopenia☐<br>3.Pancitopenia☐<br>4.Otro___________☐	☐0. No. ☐1. Si:<br>1.Tinitus-------☐<br>2.Pérdida de audición☐<br>3.Otro_______☐<br>***Otras:***<br>☐0. No. ☐1. Si:<br>______________

**Reacciones adversas grado 3 o 4:**	☐0. No. ☐1.Si
**Medicación concomitante**	☐0Azitromicina ☐1 Claritromicina ☐2Cefalosporina ☐3Carbapenémicos ☐4 Terapia biológica ☐5 Antivirales ☐6 Antiretrovirales ☐7 Esteroides sistémicos ☐8 Esteroides inhalados ☐9 Otros
**Cultivos**	☐0 No realiza ☐1 Broncoscopia ☐2 Aspirado traqueal<br>☐3 Exudado ☐4Expectoración ☐5Uresis<br>☐6 Hemocultivo ☐7 Otros ☐6 Hemocultivo ☐7Otros
**Retiro del medicamento**<br>☐0. No. ☐1.Si	**Motivo**<br>☐1 Reacciones adversas ☐2 Indicación del médico tratante ☐3Retiro del ensayo clínico<br>☐4 Otros____________________

**Factores de riesgo para QT**	
**Riesgos**	**Points**
**Edad ≥68**	**1** ☐
**Mujeres**	**1** ☐
**Diurético de asa**	**1** ☐
**m K+ ≤3.5 mEq/L**	**2** ☐
**QTc ≥450 ms al inicio**	**2** ☐
**Infarto miocárdico agudo**	**2** ☐
**≥2 drogas que prolongan QTc-**	**3** ☐

**sepsis**	3 ☐
**Falla cardíaca**	3 ☐
**Una droga que prolonga QTc**	3 ☐
**Score**	
**K+ indicates potassium; and MI, myocardial infarction.**<br>**Low risk <=6, moderate 7-10, high risk >=11.**	

**DESENLACES**		
**Número de días de adminsitración del farmaco** ☐☐	**Fecha de alta hospitalaria:** D D M M M Y Y Y	**Motivo de egreso:**<br>☐ 0 Fallece<br>☐ 1 Alta. ☐ 2 Traslado
**Ventilación no invasiva**<br>☐ 0. No. ☐ 1. Si (CPAP/BiPAP) ☐ 2.Si(Puntas de alto flujo)	D D M M M Y Y Y Y<br>**Fecha de inicio de ventilación no invasiva**	D D M M M Y Y Y Y<br>**Fecha de término de ventilación no invasiva**
**Ventilación invasiva**<br>☐ 0. No. ☐ 1. Si	D D M M M Y Y Y Y<br>**Fecha de inicio de ventilación invasiva**	D D M M M Y Y Y Y<br>**Fecha de término de ventilación invasiva**
**Reintubación**<br>☐ 0. No. ☐ 1. Si	D D M M M Y Y Y Y<br>**Fecha de re inicio de ventilación invasiva**	D D M M M Y Y Y Y<br>**Fecha de término de ventilación invasiva**
**Traqueostomía** Si ☐ No ☐	**Fecha de traqueostomía** D D D	
D D M MM Y YYY<br>**Fecha COVID día 7**<br>resultado1 ☐ negativo<br>0 ☐ positivo	D D M MM Y YYY<br>**Fecha COVID día 14**<br>resultado 1 ☐ negativo<br>0 ☐ positivo	D D M MM Y YYY<br>**Fecha COVID día 21**<br>resultado 1 ☐ negativo<br>0 ☐ positivo

Coinfecciones: ☐ 0 Ninguna ☐ 1 Staphylococcus aureus ☐ 2 Pseudomona ☐ 3 Stenotrophomonamalatophilia ☐ 4 Acinetobacter ☐ 5 klebsiella pneumoniae ☐ 6 Neumococo ☐ 7 Candida ☐ 8 otros

Notas: ______________________________

Declaración del investigador: Revisé los datos en esta Forma y confirmo que los datos están completes y son exactos.

Investigador (Nombre completo): ____

Firma ☐ 1

Fecha de firma: | D | D | M | M | M | Y | Y | Y | Y |

PATIENT ID H - ______
DATE _ _ _/ _ _/ _ _ _ _

General instructions for the form fill:

Form Fill

- All who autorize their participation must complete this form.
- For confidentially reasons in this form must be not appear complete name or initials of the patient.

General

- Plase fill all with capital letter.
- Text and comments must be briefs.
- Answer all the questions explicitly.
- All the questions must be filled. If you don't know the answer write "U" (unknown). If is not complete write "NC" (no complete).If the question is not applicable write "NA" (no aopplicable).
- If you want to select put a X

Date and Hour

- All the dates mus be written like: DD/MMM/YYY e.g 05/MAY/2009. The abbreviations of the moths are below :

January	=	JAN	May	=	MAY	September	=	SEP
February	=	FEB	June	=	JUN	October	=	OCT
March	=	MAR	July	=	JUL	November	=	NOV
April	=	Apr	August	=	AUG	December	=	DEC

In case you ignore the exact date of the event write **DD**

D	D	J	A	N	2	0	0	9
DD		MMM			AAAA			

- The hour must be filled in 24 hrs format e.g. 13:00. In mid night must be filled like 00:00

Mistakes

- **Do not write above/over the mistake, do not use liquid paper or eraser.**
- Cross out the wrong data.
- Put the right data next to the wrong one.
- Put date and initials of the person that make the correction.

PATIENT ID H - ______
DATE _ _ _/ _ _/ _ _ _ _

**PARTICIPANT INFORMATION**		
Name of the center of study	INSTITUTO NACIONAL DE ENFERMEDADES RESPIRATORIAS<br>Ismael Cosío Villegas	
Inclusion Criteria:	☐ Above 18 years<br>☐ Signed informed consent<br>☐ **Confirmed infection with COVID-19:**. by faringeal specimen and nasopharyngeal, or bronquial lavage or tracheal aspirate and/or pulmonary infection documented by RX or CT scan new opacities.<br>☐ **S**tart of symptoms less tan 14 days.<br>☐ Serious illness by COVID – 19 with at least one of the following:<br>☐ SpO2 less or same to 90 % with a height of 2240 m, in México's valley or Puebla or decrease of more tan 3% of basa saturation.<br>☐ Requieres mechanical ventilation and/or supplemental oxygen (or increseased supplemental oxygen support in chronic hipoxemia)<br>☐ Have sepsis / septic shock criteria	
Exclusion Criteria	☐ Above 80 years<br>☐ Positive pregnancy test.<br>☐ Previous allergy to chloroquine, hydroxychloroquine or amodiaquine.<br>☐ QTc>450/500mmsec male/female, EKG, Risk scale of prolonged QT interval , high risk (>=11)<br>☐ Drug with dangerous interaction with hydroxychloroquine and inability to avoid it.<br>☐ Retinopathy<br>☐ Known G6PD defincency<br>☐ History of cardiac failure, myocardial infarction, arrhythmia<br>☐ Previous ifection by COVID 2019<br>☐ Decision of the responsible physician to exclude from de study.<br>☐ Trasnferred patient to another hospital.<br>☐ Actual or previous treatment with hydroxycloroquine (a moth)<br>☐ Chronic renal or hepatic illness. (renal failure GFR less than 30mL or ChildPugh B or C).	
Inclusion Criteria:	Have all ☐1.	Doesn't have all ☐2.
Born Date	D D M M M A A A A	Age (years)
Gender	☐1 Male ☐0 Female	
Date y hour of admission to the emergency department	D D M M M A A A A	H H : M M
Recruiting site:	☐1 Emergency deparment ☐2 Floor ☐3 ICU	
Inclusion date (ramdomization date)	D D M M M A A A A	
Date of PCR SARS COV-2	D D M M M A A A A	
Viral coinfection	☐0 None ☐1 influenza A ☐2 Influenza B<br>☐3 H1N1 ☐4 H1 ☐5 H3<br>☐6 H5 ☐7 H7N9	

PATIENT ID H - ______
DATE _ _ _/ _ _/ _ _ _ _

**BASAL SYMPTOMS**			
Syntoms Start Date	D D M M M Y Y Y Y	O ☐1 Unknown	
1Anorexia	☐1. Yes	☐0. No	Duration: _______ days
2Arthralgia	☐1. Yes	☐0. No	Duration: _______ days
3 Headache	☐1. Yes	☐0. No	Duration: _______ days
4Conjunctivitis	☐1. Yes	☐0. No	Duration: _______ days
5Diarrhea	☐1. Yes	☐0. No	Duration: _______ days
6Dyspnea	☐1. Yes	☐0. No	Duration: _______ days
7Shivers	☐1. Yes	☐0. No	Duration: _______ days
8Sputum	☐1. Yes	☐0. No	Duration: _______ days
9Fatigue	☐1. Yes	☐0. No	Duration: _______ days
10Fever	☐1. Yes	☐0. No	Duration: _______ days
11Malaise	☐1. Yes	☐0. No	Duration: _______ days
12Nausea	☐1. Yes	☐0. No	Duration: _______ days
13Sorethroad	☐1. Yes	☐0. No	Duration: _______ days
14 Productive cough	☐1. Yes	☐0. No	Duration: _______ days
15Dry cough	☐1. Yes	☐0. No	Duration: _______ days
16Vomit	☐1. Yes	☐0. No	Duration: _______ days
17Other Symptom:	____________________		Duration: _______ days

PATIENT ID H - ______
DATE _ _ _/ _ _/ _ _ _ _

COMORBIDITIES

Person con comorbidities? ☐$_0$No ☐$_1$ Yes Please specify rhe comorbidities :

Cod	Title		Cod	Title	
1	Neurological Illness		11	Use of inhaled corticoesteroids	
2	Cardiac Illnes		12	Use of immunosuppressants drugs	
3	Arterial Hypertension		13	Diabetes mellitus	
3a	Uso de Inhibidores de ECA (captopril, enalapril, etc.)		14	HIV	
4	ASTHMA		15	Chronic Renal Illness	
5	COPD		16	Hepatic Illness	
6	Interstitial Lung Disease		17	Obesity	
7	Tuberculosis		18	Use of ibuprofen (or NSAIDs)	
8	Autoimmune Disease		19	Systemic Lupus Erythematosus, Rheumatoid arthritis or other autoimmune disease Specify______________________	
9	Alcoholism		20	Cancer ________________	
10	Use of oral corticoesteroids				

**Vaccination history**	Influenza 2019-2020 ☐$_0$No. ☐$_1$.Yes ☐$_2$.unknown BCG ☐$_0$. No. ☐$_1$.Yes ☐$_2$.unknown Pneumococcus ☐$_0$. No. ☐$_1$.Yes ☐$_2$.unknown

**TABAQUISM**		
Actual Tabaquism ☐$_0$. **No.** ☐$_1$.**Yes**	N° cigarette at day: ___	N° of year smoking: ___
Previous Tabaquism ☐$_0$. **No.** ☐$_1$.**Yes**	Age of start:	Age of quit:
Use of electronic cigarette in the least moth ☐$_0$. **No.** ☐$_1$. **Yes**	Kind of electronic cigarette: ☐$_1$. JUUL. ☐$_2$.IQOS ☐$_3$. Others ☐$_{4"}$. No apply	
History of marijuana use in the least month	☐$_0$. No. ☐$_1$.Yes	

BASAL STATUS

Datos obtenidos el día de la aleatorización; si existen múltiples mediciones durante ese día se toma el peor valor obtenido.

**Oxigenoterapia puntas nasales**	☐0. No<br>☐1. Yes	**Liters per minute** _____	**DATE** ___/___/___ D D M M M A A A A	**Date of start**
**Non invasive ventilation** at the moment of ramdomization	☐0. No<br>☐1.Yes (CPAP/BiPAP)	☐2.Yes (High Flow Nasal Cannula)	D D M M M A A A A	**Date of start**
**Invasive ventilation** at the moment of ramdomization	☐0. No	☐1. Yes (Fill the parameters in the following)	D D M M M Y Y Y Y	**Orotraqueal intubation date**
**Uso de aminas** at the moment of ramdomization	☐0. No	☐1. Yes		

Daily monitoring complementary exams

Day

If there are multiple measurements, write down the worst ones taken from 00:00am to 12:00–

Date<br>D D M M M A A A A	Hour 24 HRS	Lactate (mmol/L)	pO2 (mmHg))	FiO_2(0.21 a 1)
pCO_2 (mmHg)	pH	Leukocytes($10^3/mm^3$)	Platelets ($10^3/mm^3$)	SO_2(%)
Creatinine (mg/dL)	Potassium (mEq/L)	Neutrofilos($10^3/mm^3$	Eosinophils($10^3/mm^3$	Lymphocytes ($10^3/mm^3$)
BUN (mg/dL)	PT (s)	PTT (s)	INR	
Sodium (mEq/L)	Total bilirubin (mg/dL)	Direct bilirubin(mg/dL)	AST (UI/L)	ALT (UI/L)
BNP	Dimer D	PCR	Ferritin	Procalcitonin

Signos vitales

SYSTOLIC BP (mmHg)	DIASTOLIC BP (mmHg)	BPM (lpm)	F (Rpm)	Temperature °C	Weight _________<br>Height _________

Glasgow Scale<br>☐0 **15Points** ☐1 13-14**Points**<br>☐2 10-12**Points** ☐3 6-9**Points**<br>☐4 <6**Points** ☐5 No apply	Urinary Flow Rate<br>☐0 >500 ml/dl<br>☐1 <500 ml/dl<br>☐2 <200 ml/dl	Cardiovascular/amines<br>☐0 PAM<70 mmHg<br>☐1 Dopamine <5 o Dubatamine any dose<br>☐2 Dopamine 5.1-15 or Epi or Norepinephrine <0.1<br>☐3 Dopamine >15 or Epi or Norepinephrine >0.1

With non mechanical ventilation: ☐0. No. ☐1.Yes (CPAP/BiPAP) ☐2 Yes(High flow nasal cannula)

With mechanical ventilation: ☐0. No. ☐1. Yes

In case of invasive mechanical ventilation, fill the following parameters

FiO_2 (0.21 al 1)	Mid Preassure (cmH2O)	Tidal volume (ml)	PEEP (cmH2O)	F (rpm)	Plateu Pressure (cmH2O)

PATIENT ID H - ______
DATE _ _ _/ _ _/ _ _ _ _

Any adverse raction? ☐0. No. ☐1. Yes ☐2. Basal

Visuals<br>☐0. No. ☐1. Yes:<br>1.Vission Loss------☐<br>2.Other_______________☐<br>***Cardiovasculars***<br>☐0. No. ☐1. Yes<br>1.Hipotension----------☐<br>2.QTc prolongation----☐<br>3.T wave depression-☐<br>4.Other_______________☐	***Neurologicals***<br>☐0. No. ☐1. Yes<br>1.Agitation/Confussion ☐<br>2.Dizziness---------☐<br>3.Headache-------☐<br>4.Paresthesia---☐<br>5.Seizures-☐<br>6.Neurophaty---☐<br>7.Other____________☐	***Hepatobiliar***<br>☐0. No. ☐1. Yes:<br>1.Trabsanubase elevation -☐<br>2.Other ☐<br>______________<br>***Gastrointestinal***<br>☐0. No. ☐1.Yes:<br>1.Vomit ☐<br>2.Nausea☐<br>3.Diarrhea----☐<br>4.Other_____________<br>________☐	***Dermatological***<br>☐0. No. ☐1. Yes:<br>1.Itch-------------☐<br>2.Alopecia--------- -☐<br>3.Eritema/hives-☐<br>4.Otro_____________☐<br>***Hematological***<br>☐0. No. ☐1. Yes<br>1.Agranulocytosis☐<br>2.Thrombocytopenia ☐<br>3.Pancytopenia☐<br>4.Other___________ ☐	***Auditive***<br>☐0. No. ☐1. Yes:<br>1.Tinnitus-------☐<br>2. Audition loss☐<br>3.Other________☐<br>***Others:***<br>☐0. No. ☐1. Yes:<br>________________

**Adverse reactions grade 3 or 4:**	☐0. No. ☐1.Yes ☐2 .Basal
**Concomitant dugs**	☐0 None ☐1Azithromicine ☐2 Clarithromycin ☐3Cephalosporin ☐4Carbapenemics ☐5 Biologic therapy ☐6 Antivirals ☐7 Antiretrovirals ☐8 Systemic steroids ☐9 inhaled steroids ☐10 Others ___________________________
**Cultives**	☐0 None ☐1 Bronchoscopy ☐2 Traqueal aspirate ☐3 Exudate ☐4Expectoration ☐5Uresis ☐6 Blood culture ☐7 Others __________________
**Cultive Results:**	
**Remove medication**	**Motive**<br>☐0 No apply ☐1 Adverse reactions ☐2 Decision of the responsible physician ☐3Remove

PATIENT ID H - ______
DATE _ _ _/ _ _/ _ _ _ _

☐0. No. ☐1.Yes	from the clinical trial ☐4 Others___________________

Risk factors of QT prolongation

Risk	Points
Age ≥68	1 ☐
Famale sex	1 ☐
Loop Diuretic	1 ☐
m K+ <3.5 mEq/L	2 ☐
QTc ≥450 ms at the start __________	2 ☐
Acute myocardial infarction	2 ☐
≥2 drugs that prolongate the QTc-	3 ☐
Sepsis	3 ☐
Heart Failure	3 ☐
One drug that prolongate the QTc____	3 ☐
Score	
(K+: potassium) Low risk <=6, moderate 7-10, high risk >=11.	

Outcomes		
Number of days the drug was admistered ☐☐	Date when patient left the hospital: D D M M M Y Y Y	Motive of egress: ☐0 Die ☐1 Home. ☐2Transfer
Non mechanical ventilation ☐0. No. ☐1. Yes (CPAP/BiPAP) ☐2.Yes(high flow nasal cannula)	D D M M M Y Y Y Y Date when non mechanical ventilation starts	D D M M M Y Y Y Y Date when non mechanical ventilation was over
Mechanical Ventilation ☐0. No. ☐1. Yes	D D M M M Y Y Y Y Date when mechanical ventilation starts	D D M M M Y Y Y Y Date when mechanical ventilation was over

PATIENT ID H - ______
DATE _ _ _/ _ _/ _ _ _ _

**Reintubation**<br>☐0. No. ☐1. Yes	D D M M M Y Y Y Y<br>**Date of mechanical ventilation restart**	D D M M M Y Y Y Y<br>**Date of mechanical ventilation ends**
**Tracheotomy** Yes ☐ No ☐	**Date of tracheotomy** D D D	

D D M MM Y YYY	D D M MM Y YYY	D D M MM Y YYY
**Date COVID day 7**<br>Result 1 ☐ negative<br>0 ☐ positive	**Date COVID day 14**<br>Result 1 ☐ negative<br>0 ☐ positive	**Date COVID day 21**<br>Result 1 ☐ negative<br>0 ☐ positive

Coinfections: ☐ 0 None ☐ 1 Staphylococcus aureus ☐ 2 Pseudomonas ☐ 3 Stenotrophomonamalatophilia
☐ 4 Acinetobacter ☐ 5 klebsiella pneumoniae ☐ 6 Pneumococo ☐ 7 Candida ☐ 8 others

Notes: ____

Investigator statement: I reviwed the data on this forms and confirm that the data is complete and accurate.

Investigator (Full name): ____

Sign ☐1

Date of sign: D D M M M Y Y Y Y

CARDIOVASCULARES

PARAMETER	GRADE 1<br>MILD	GRADE 2<br>MODERATE	GRADE 3<br>SEVERE	GRADE 4<br>POTENTIALLY LIFE-THREATENING
**Clinical** adverse event <u>NOT</u> identified elsewhere in the grading table	Mild symptoms causing no or minimal interference with usual social & functional activities with intervention not indicated	Moderate symptoms causing greater than minimal interference with usual social & functional activities with intervention indicated	Severe symptoms causing inability to perform usual social & functional activities with intervention or hospitalization indicated	Potentially life-threatening symptoms causing inability to perform basic self-care functions with intervention indicated to prevent permanent impairment, persistent disability, or death
**Cardiac Ischemia or Infarction**<br>*Report only one*	NA	NA	New symptoms with ischemia (stable angina) <u>OR</u> New testing consistent with ischemia	Unstable angina <u>OR</u> Acute myocardial infarction
**Heart Failure**	No symptoms <u>AND</u> Laboratory or cardiac imaging abnormalities	Symptoms with mild to moderate activity or exertion	Symptoms at rest or with minimal activity or exertion (e.g., hypoxemia) <u>OR</u> Intervention indicated (e.g., oxygen)	Life-threatening consequences <u>OR</u> Urgent intervention indicated (e.g., vasoactive medications, ventricular assist device, heart transplant)
**Arrhythmia**<br>(by ECG or physical examination)<br>*Specify type, if applicable*	No symptoms <u>AND</u> No intervention indicated	No symptoms <u>AND</u> Non-urgent intervention indicated	Non-life-threatening symptoms AND Non-urgent intervention indicated	Life-threatening arrhythmia <u>OR</u> Urgent intervention indicated
**Prolonged PR Interval or AV Block**<br>*Report only one*<br>*> 16 years of age*	PR interval 0.21 to < 0.25 seconds	PR interval ≥ 0.25 seconds <u>OR</u> Type I 2nd degree AV block	Type II 2nd degree AV block <u>OR</u> Ventricular pause ≥ 3.0 seconds	Complete AV block
≤ 16 years of age	1st degree AV block (PR interval > normal for age and rate)	Type I 2nd degree AV block	Type II 2nd degree AV block <u>OR</u> Ventricular pause ≥ 3.0 seconds	Complete AV block
**Prolonged QTc Interval**[2]	0.45 to 0.47 seconds	> 0.47 to 0.50 seconds	> 0.50 seconds <u>OR</u> ≥ 0.06 seconds above baseline	Life-threatening consequences (e.g., Torsade de pointes, other associated serious ventricular dysrhythmia)

GASTROINTESTINAL

PARAMETER	GRADE 1 MILD	GRADE 2 MODERATE	GRADE 3 SEVERE	GRADE 4 POTENTIALLY LIFE-THREATENING
**Clinical** adverse event <u>**NOT**</u> identified elsewhere in the grading table	Mild symptoms causing no or minimal interference with usual social & functional activities with intervention not indicated	Moderate symptoms causing greater than minimal interference with usual social & functional activities with intervention indicated	Severe symptoms causing inability to perform usual social & functional activities with intervention or hospitalization indicated	Potentially life-threatening symptoms causing inability to perform basic self-care functions with intervention indicated to prevent permanent impairment, persistent disability, or death
**Ascites**	No symptoms	Symptoms <u>AND</u> Intervention indicated (e.g., diuretics, therapeutic paracentesis)	Symptoms recur or persist despite intervention	Life-threatening consequences
**Diarrhea**<br>*≥ 1 year of age*	Transient or intermittent episodes of unformed stools <u>OR</u> Increase of ≤ 3 stools over baseline per 24-hour period	Persistent episodes of unformed to watery stools <u>OR</u> Increase of 4 to 6 stools over baseline per 24-hour period	Increase of ≥ 7 stools per 24-hour period <u>OR</u> IV fluid replacement indicated	Life-threatening consequences (e.g., hypotensive shock)
< 1 year of age	Liquid stools (more unformed than usual) but usual number of stools	Liquid stools with increased number of stools <u>OR</u> Mild dehydration	Liquid stools with moderate dehydration	Life-threatening consequences (e.g., liquid stools resulting in severe dehydration, hypotensive shock)
**Nausea**	Transient (< 24 hours) or intermittent <u>AND</u> No or minimal interference with oral intake	Persistent nausea resulting in decreased oral intake for 24 to 48 hours	Persistent nausea resulting in minimal oral intake for > 48 hours <u>OR</u> Rehydration indicated (e.g., IV fluids)	Life-threatening consequences (e.g., hypotensive shock)
**Vomiting**	Transient or intermittent <u>AND</u> No or minimal interference with oral intake	Frequent episodes with no or mild dehydration	Persistent vomiting resulting in orthostatic hypotension <u>OR</u> Aggressive rehydration indicated (e.g., IV fluids)	Life-threatening consequences (e.g., hypotensive shock)

NEUROLÓGICO

**PARAMETER**	**GRADE 1 MILD**	**GRADE 2 MODERATE**	**GRADE 3 SEVERE**	**GRADE 4 POTENTIALLY LIFE-THREATENING**
**Clinical** adverse event NOT identified elsewhere in the grading table	Mild symptoms causing no or minimal interference with usual social & functional activities with intervention not indicated	Moderate symptoms causing greater than minimal interference with usual social & functional activities with intervention indicated	Severe symptoms causing inability to perform usual social & functional activities with intervention or hospitalization indicated	Potentially life-threatening symptoms causing inability to perform basic self-care functions with intervention indicated to prevent permanent impairment, persistent disability, or death
**Altered Mental Status** (for Dementia, see *Cognitive, Behavioral, or Attentional Disturbance* below)	Changes causing no or minimal interference with usual social & functional activities	Mild lethargy or somnolence causing greater than minimal interference with usual social & functional activities	Confusion, memory impairment, lethargy, or somnolence causing inability to perform usual social & functional activities	Delirium OR Obtundation OR Coma
**Cognitive, Behavioral, or Attentional Disturbance** (includes dementia and attention deficit disorder)<br>*Specify type, if applicable*	Disability causing no or minimal interference with usual social & functional activities OR Specialized resources not indicated	Disability causing greater than minimal interference with usual social & functional activities OR Specialized resources on part-time basis indicated	Disability causing inability to perform usual social & functional activities OR Specialized resources on a full-time basis indicated	Disability causing inability to perform basic self-care functions OR Institutionalization indicated
**Headache**	Symptoms causing no or minimal interference with usual social & functional activities	Symptoms causing greater than minimal interference with usual social & functional activities	Symptoms causing inability to perform usual social & functional activities	Symptoms causing inability to perform basic self-care functions OR Hospitalization indicated OR Headache with significant impairment of alertness or other neurologic function

RESPIRATORIO

PARAMETER	GRADE 1 MILD	GRADE 2 MODERATE	GRADE 3 SEVERE	GRADE 4 POTENTIALLY LIFE-THREATENING
**Clinical** adverse event **NOT** identified elsewhere in the grading table	Mild symptoms causing no or minimal interference with usual social & functional activities with intervention not indicated	Moderate symptoms causing greater than minimal interference with usual social & functional activities with intervention indicated	Severe symptoms causing inability to perform usual social & functional activities with intervention or hospitalization indicated	Potentially life-threatening symptoms causing inability to perform basic self-care functions with intervention indicated to prevent permanent impairment, persistent disability, or death
**Dyspnea or Respiratory Distress** *Report only one*	Dyspnea on exertion with no or minimal interference with usual social & functional activities OR Wheezing OR Minimal increase in respiratory rate for age	Dyspnea on exertion causing greater than minimal interference with usual social & functional activities OR Nasal flaring OR Intercostal retractions OR Pulse oximetry 90 to < 95%	Dyspnea at rest causing inability to perform usual social & functional activities OR Pulse oximetry < 90%	Respiratory failure with ventilator support indicated (e.g., CPAP, BPAP, intubation)

SENSORIAL

PARAMETER	GRADE 1 MILD	GRADE 2 MODERATE	GRADE 3 SEVERE	GRADE 4 POTENTIALLY LIFE-THREATENING
**Clinical** adverse event **NOT** identified elsewhere in the grading table	Mild symptoms causing no or minimal interference with usual social & functional activities with intervention not indicated	Moderate symptoms causing greater than minimal interference with usual social & functional activities with intervention indicated	Severe symptoms causing inability to perform usual social & functional activities with intervention or hospitalization indicated	Potentially life-threatening symptoms causing inability to perform basic self-care functions with intervention indicated to prevent permanent impairment, persistent disability, or death
**Hearing Loss** *≥ 12 years of age*	NA	Hearing aid or intervention not indicated	Hearing aid or intervention indicated	Profound bilateral hearing loss (> 80 dB at 2 kHz and above) OR Non-serviceable hearing (i.e., >50 dB audiogram and <50% speech discrimination)
**Visual Changes** (assessed from baseline)	Visual changes causing no or minimal interference with usual social & functional activities	Visual changes causing greater than minimal interference with usual social & functional activities	Visual changes causing inability to perform usual social & functional activities	Disabling visual loss in affected eye(s)

SISTÉMICO

PARAMETER	GRADE 1 MILD	GRADE 2 MODERATE	GRADE 3 SEVERE	GRADE 4 POTENTIALLY LIFE-THREATENING
**Clinical** adverse event NOT identified elsewhere in the grading table	Mild symptoms causing no or minimal interference with usual social & functional activities with intervention not indicated	Moderate symptoms causing greater than minimal interference with usual social & functional activities with intervention indicated	Severe symptoms causing inability to perform usual social & functional activities with intervention or hospitalization indicated	Potentially life-threatening symptoms causing inability to perform basic self-care functions with intervention indicated to prevent permanent impairment, persistent disability, or death
**Acute Allergic Reaction**	Localized urticaria (wheals) with no medical intervention indicated	Localized urticaria with intervention indicated OR Mild angioedema with no intervention indicated	Generalized urticaria OR Angioedema with intervention indicated OR Symptoms of mild bronchospasm	Acute anaphylaxis OR Life-threatening bronchospasm OR Laryngeal edema
**Chills**	Symptoms causing no or minimal interference with usual social & functional activities	Symptoms causing greater than minimal interference with usual social & functional activities	Symptoms causing inability to perform usual social & functional activities	NA
**Cytokine Release Syndrome**[8]	Mild signs and symptoms AND Therapy (i.e., antibody infusion) interruption not indicated	Therapy (i.e., antibody infusion) interruption indicated AND Responds promptly to symptomatic treatment OR Prophylactic medications indicated for ≤ 24 hours	Prolonged severe signs and symptoms OR Recurrence of symptoms following initial improvement	Life-threatening consequences (e.g., requiring pressor or ventilator support)
**Fatigue or Malaise** *Report only one*	Symptoms causing no or minimal interference with usual social & functional activities	Symptoms causing greater than minimal interference with usual social & functional activities	Symptoms causing inability to perform usual social & functional activities	Incapacitating symptoms of fatigue or malaise causing inability to perform basic self-care functions
**Fever** (non-axillary temperatures only)	38.0 to < 38.6°C or 100.4 to < 101.5°F	≥ 38.6 to < 39.3°C or ≥ 101.5 to < 102.7°F	≥ 39.3 to < 40.0°C or ≥ 102.7 to < 104.0°F	≥ 40.0°C or ≥ 104.0°F
**Pain**[9] (not associated with study agent injections and not specified elsewhere) *Specify location*	Pain causing no or minimal interference with usual social & functional activities	Pain causing greater than minimal interference with usual social & functional activities	Pain causing inability to perform usual social & functional activities	Disabling pain causing inability to perform basic self-care functions OR Hospitalization indicated

LABORATORIOS

PARAMETER	GRADE 1 MILD	GRADE 2 MODERATE	GRADE 3 SEVERE	GRADE 4 POTENTIALLY LIFE-THREATENING
**Clinical** adverse event <u>**NOT**</u> identified elsewhere in the grading table	Mild symptoms causing no or minimal interference with usual social & functional activities with intervention not indicated	Moderate symptoms causing greater than minimal interference with usual social & functional activities with intervention indicated	Severe symptoms causing inability to perform usual social & functional activities with intervention or hospitalization indicated	Potentially life-threatening symptoms causing inability to perform basic self-care functions with intervention indicated to prevent permanent impairment, persistent disability, or death
**Albumin, Low** (g/dL; *g/L*)	3.0 to < LLN<br>*30 to < LLN*	≥ 2.0 to < 3.0<br>*≥ 20 to < 30*	< 2.0<br>*< 20*	NA
**Alkaline Phosphatase, High**	1.25 to < 2.5 x ULN	2.5 to < 5.0 x ULN	5.0 to < 10.0 x ULN	≥ 10.0 x ULN
**ALT or SGPT, High**<br>*Report only one*	1.25 to < 2.5 x ULN	2.5 to < 5.0 x ULN	5.0 to < 10.0 x ULN	≥ 10.0 x ULN
**AST or SGOT, High**<br>*Report only one*	1.25 to < 2.5 x ULN	2.5 to < 5.0 x ULN	5.0 to < 10.0 x ULN	≥ 10.0 x ULN
**Bilirubin**<br>***Direct Bilirubin[13], High***<br>*> 28 days of age*	NA	NA	> ULN with other signs and symptoms of hepatotoxicity.	> ULN with life-threatening consequences (e.g., signs and symptoms of liver failure)
Total Bilirubin, High<br>*> 28 days of age*	1.1 to < 1.6 x ULN	1.6 to < 2.6 x ULN	2.6 to < 5.0 x ULN	≥ 5.0 x ULN
**Calcium, High**<br>(mg/dL; *mmol/L*)<br>*≥ 7 days of age*	10.6 to < 11.5<br>*2.65 to < 2.88*	11.5 to < 12.5<br>*2.88 to < 3.13*	12.5 to < 13.5<br>*3.13 to < 3.38*	≥ 13.5<br>*≥ 3.38*
**Calcium, Low**<br>(mg/dL; *mmol/L*)<br>*≥ 7 days of age*	7.8 to < 8.4<br>*1.95 to < 2.10*	7.0 to < 7.8<br>*1.75 to < 1.95*	6.1 to < 7.0<br>*1.53 to < 1.75*	< 6.1<br>*< 1.53*

**Cardiac Troponin I, High**	NA	NA	NA	Levels consistent with myocardial infarction or unstable angina as defined by the local laboratory
**Creatine Kinase, High**	3 to < 6 x ULN	6 to < 10x ULN	10 to < 20 x ULN	≥ 20 x ULN
**Creatinine, High**<br>**Report only one*	1.1 to 1.3 x ULN	> 1.3 to 1.8 x ULN <u>OR</u> Increase to 1.3 to < 1.5 x participant's baseline	> 1.8 to < 3.5 x ULN <u>OR</u> Increase to 1.5 to < 2.0 x participant's baseline	≥ 3.5 x ULN <u>OR</u> Increase of ≥ 2.0 x participant's baseline
**Creatinine Clearance[14] or eGFR, Low**<br>**Report only one*	NA	< 90 to 60 ml/min or ml/min/1.73 m^2<br><u>OR</u><br>10 to < 30% decrease from participant's baseline	< 60 to 30 ml/min or ml/min/1.73 m^2<br><u>OR</u><br>30 to < 50% decrease from participant's baseline	< 30 ml/min or ml/min/1.73 m^2<br><u>OR</u><br>≥ 50% decrease from participant's baseline or dialysis needed
**Glucose**<br>(mg/dL; *mmol/L*)<br>***Fasting, High***	110 to 125<br>*6.11 to < 6.95*	> 125 to 250<br>*6.95 to < 13.89*	> 250 to 500<br>*13.89 to < 27.75*	≥ 500<br>*≥ 27.75*
Nonfasting, High	116 to 160<br>*6.44 to < 8.89*	> 160 to 250<br>*8.89 to < 13.89*	> 250 to 500<br>*13.89 to < 27.75*	≥ 500<br>*≥ 27.75*
**Glucose, Low**<br>(mg/dL; *mmol/L*)<br>*≥ 1 month of age*	55 to 64<br>*3.05 to <3.55*	40 to < 55<br>*2.22 to < 3.05*	30 to < 40<br>*1.67 to < 2.22*	< 30<br>*< 1.67*
< 1 month of age	50 to 54<br>*2.78 to < 3.00*	40 to < 50<br>*2.22 to < 2.78*	30 to < 40<br>*1.67 to < 2.22*	< 30<br>*< 1.67*
**Lactate, High**	ULN to < 2.0 x ULN without acidosis	≥ 2.0 x ULN without acidosis	Increased lactate with pH < 7.3 without life-threatening consequences	Increased lactate with pH < 7.3 with life-threatening consequences
**Magnesium[15], Low**<br>(mEq/L; *mmol/L*)	1.2 to < 1.4<br>*0.60 to < 0.70*	0.9 to < 1.2<br>*0.45 to < 0.60*	0.6 to < 0.9<br>*0.30 to < 0.45*	< 0.6<br>*< 0.30*
**Phosphate, Low**<br>(mg/dL; *mmol/L*)<br>*> 14 years of age*	2.0 to < LLN<br>*0.65 to < LLN*	1.4 to < 2.0<br>*0.45 to < 0.65*	1.0 to < 1.4<br>*0.32 to < 0.45*	< 1.0<br>*< 0.32*
**Potassium, High**<br>(mEq/L; *mmol/L*)	5.6 to < 6.0<br>*5.6 to < 6.0*	6.0 to < 6.5<br>*6.0 to < 6.5*	6.5 to < 7.0<br>*6.5 to < 7.0*	≥ 7.0<br>*≥ 7.0*
**Potassium, Low**<br>(mEq/L; *mmol/L*)	3.0 to < 3.4<br>*3.0 to < 3.4*	2.5 to < 3.0<br>*2.5 to < 3.0*	2.0 to < 2.5<br>*2.0 to < 2.5*	< 2.0<br>*< 2.0*
**Sodium, High**<br>(mEq/L; *mmol/L*)	146 to < 150<br>*146 to < 150*	150 to < 154<br>*150 to < 154*	154 to < 160<br>*154 to < 160*	≥ 160<br>*≥ 160*
**Sodium, Low**<br>(mEq/L; *mmol/L*)	130 to < 135<br>*130 to < 135*	125 to < 130<br>*125 to < 130*	121 to < 125<br>*121 to < 125*	≤ 120<br>*≤ 120*
**Uric Acid, High**<br>(mg/dL; *mmol/L*)	7.5 to < 10.0<br>*0.45 to < 0.59*	10.0 to < 12.0<br>*0.59 to < 0.71*	12.0 to < 15.0<br>*0.71 to < 0.89*	≥ 15.0<br>*≥ 0.89*

**Absolute Neutrophil Count (ANC), Low**<br>(cells/mm³; *cells/L*)<br>*> 7 days of age*	800 to 1,000<br>*0.800 x 10^9 to 1.000 x 10^9*	600 to 799<br>*0.600 x 10^9 to 0.799 x 10^9*	400 to 599<br>*0.400 x 10^9 to 0.599 x 10^9*	< 400<br>*< 0.400 x 10^9*
**INR, High**<br>(not on anticoagulation therapy)	1.1 to < 1.5 x ULN	1.5 to < 2.0 x ULN	2.0 to < 3.0 x ULN	≥ 3.0 x ULN
**Hemoglobin[16], Low**<br>(g/dL; *mmol/L*)[17]<br>*≥ 13 years of age (male only)*	10.0 to 10.9<br>*6.19 to 6.76*	9.0 to < 10.0<br>*5.57 to < 6.19*	7.0 to < 9.0<br>*4.34 to < 5.57*	< 7.0<br>*< 4.34*
≥ 13 years of age (female only)	9.5 to 10.4<br>*5.88 to 6.48*	8.5 to < 9.5<br>*5.25 to < 5.88*	6.5 to < 8.5<br>*4.03 to < 5.25*	< 6.5<br>*< 4.03*
**PTT, High**<br>(not on anticoagulation therapy)	1.1 to < 1.66 x ULN	1.66 to < 2.33 x ULN	2.33 to < 3.00 x ULN	≥ 3.00 x ULN
**Platelets, Decreased**<br>(cells/mm³; *cells/L*)	100,000 to < 125,000<br>*100.000 x 10^9 to < 125.000 x 10^9*	50,000 to < 100,000<br>*50.000 x 10^9 to < 100.000 x 10^9*	25,000 to < 50,000<br>*25.000 x 10^9 to < 50.000 x 10^9*	< 25,000<br>*< 25.000 x 10^9*
**PT, High**<br>(not on anticoagulation therapy	1.1 to < 1.25 x ULN	1.25 to < 1.50 x ULN	1.50 to < 3.00 x ULN	≥ 3.00 x ULN
**WBC, Decreased**<br>(cells/mm³; *cells/L*)<br>*> 7 days of age*	2,000 to 2,499<br>*2.000 x 10^9 to 2.499 x 10^9*	1,500 to 1,999<br>*1.500 x 10^9 to 1.999 x 10^9*	1,000 to 1,499<br>*1.000 x 10^9 to 1.499 x 10^9*	< 1,000<br>*< 1.000 x 10^9*

SALUD
SECRETARÍA DE SALUD

INER

Instituto Nacional de Enfermedades Respiratorias
'Ismael Cosío Villegas'

FORMATO DE REPORTE DE CASO

Protocolo C13 20 HYDRA
HIDROXICLOROQUINA PARA EL TRATAMIENTO DE INFECCIÓN RESPIRATORIA GRAVE POR COVID-19: ENSAYO CLÍNICO CONTROLADO MULTICÉNTRICO

IDdel Sujeto: H-______ - ☐☐☐

Fecha de nacimiento: ____________________ **Edad:**_____años______meses
DD MMM AAAA

Fecha de firma de consentimientoinformado:____________________
DD MMM AAAA

SEGUIMIENTO PROGRAMADO	Fecha de realización	¿Se realizo?
ESCRUTINIO/ ALEATORIZACIÓN		
Día 1		
Día 2		
Día 3		
Día 4		
Día 5		
Día 6		
Día 7		
Día 8		
Día 9		
Día 10		
Desenlace (seguimiento día 30)		

ANEXO 1. FINALIZACIÓN DEL ESTUDIO

	No	Si
¿Terminó el paciente el estudio como estaba planeado?		

- Si contestó **SI**, por favor firme al final de estahoja.
- Si contestó **NO**, continúe con los siguientesincisos.

Fecha en que **abandonó/se retiró** del estudio (DD/MMM/AAAA):	
Fecha de la **última administración** del medicamento del estudio (DD/MMM/AAAA):	
Fecha de **última evaluación** clínica del estudio (Última visita realizada) (DD/MMM/AAAA):	

Seleccione la razón por la que se suspendió el estudio:

☐ Evento adverso 3- 4

☐ Enfermedad aguda que contraindica el fármaco

☐ Retiro del consentimiento por el paciente/ responsable legal

☐ Retiro del consentimiento por del médico tratante

☐ Traslado a otra Unidad Hospitalaria

☐ Otro (Especificar): ____

Certifico que toda la información contenida en este Formato de Reporte de Caso es fidedigna y fue obtenida en concordancia con los lineamientos que establecen las Buenas Prácticas de Investigación Clínica y Regulaciones Nacionales e Internacionales.

Firma delinvestigadorprincipal **Fecha (DD/MMM/AAAA)**

INER

ANEXO 2. MEDICACIÓN CONCOMITANTE

	NOMBRE GENÉRICO	DOSIS DIARIA	INDICACIÓN PATOLOGÍA	VÍA *	INICIO DD/MMM/AAAA	TÉRMINO DD/MMM/AAAA
1						
2						
3						
4						
5						
6						
7						
8						
9						
10						
11						
12						
13						
14						
15						
16						
17						
18						
19						
20						

* **(1)** Oral **(2)** Intramuscular **(3)** Intravascular **(4)** Inhalado **(5)** Tópico

ANEXO 3. DESVIACIONES Y VIOLACIONES

	DESCRIPCIÓN	TIPO	FECHA	ACCIONES	SE NOTIFICÓ A CEI	FECHA DE NOTIFICACIÓN
		**1.** Desviación<br>**2.** Violación	DD/MMM/AAAA	DESCRIBIR	**0.** No **1.** Si	DD/MMM/AAAA
1						
2						
3						
4						
5						
6						
7						
8						
9						
10						

ID: H- _ _ _ - _ _ _

ANEXO 4. EVENTOS ADVERSOS

	EVENTO ADVERSO	INTENSIDAD	EAS	INICIO	TÉRMINO	RELACIÓN CON MEDICAMENTO DE ESTUDIO	ACCIONES	DESENLACE
	Diagnóstico	**1.** Leve<br>**2.** Moderado<br>**3.** Serio	**0.** No<br>**1.** Si	DD/MMM/AAAA	DD/MMM/AAAA	**1.** Ninguna **2.** Dudosa<br>**3.** Posible **4.**Probable<br>**5.** Muy probable	**1.** Ninguna **2.** Medicación concomitante<br>**3.** Discontinuación del medicamento deestudio<br>**4.** Hospitalización<br>**5.** Otro(especificar)	**1.** Resuelto **2.**Mejoría<br>**3.** Sincambio<br>**4.** Empeoró **5.**Muerte<br>**6.** Pérdida de seguimiento
**1**								
**2**								
**3**								
**4**								
**5**								
**6**								

Dirección de Investigación
Subdirección de Investigación Clínica
Depto. de Investigación en Tabaquismo y EPOC

INER/DITABE//2020
Ciudad de México a 7 de mayo 2020.

Asunto: Respuesta a observaciones del Comité de Investigación y Ética

Presidente del Comité de Ética en Investigación
Presidente del Comité de Investigación

Instituto Nacional de Enfermedades Respiratorias Ismael Cosío Villegas

P R E S E N T E

En relación a las observaciones realizadas el día 7 de mayo del presente año con número de oficio INER/CEI/144/20 al protocolo titulado **HIDROXICLOROQUINA PARA EL TRATAMIENTO DE INFECCIÓN RESPIRATORIA GRAVE POR COVID-19: ENSAYO CLÍNICO CONTROLADO MULTICÉNTRICO,** con código institucional C13-20 del cual soy investigadora principal, me permito subrayar los siguientes puntos:

1. El protocolo **HIDROXICLOROQUINA PARA EL TRATAMIENTO DE INFECCIÓN RESPIRATORIA GRAVE POR COVID-19: ENSAYO CLÍNICO CONTROLADO MULTICÉNTRICO**, fue sometido como un ensayo clínico aleatorizado cegado con estratificación en relación al uso de ventilación mecánica en el momento de la aleatorización. Tenemos dos grandes grupos aquellos pacientes que en el momento de la aleatorización ya se encuentran con apoyo de ventilación mecánica y el otro grupo aquellos que no lo presentan.

2. La obligación que me atañe como investigadora y del resto del equipo de investigadores es el reporte de cualquier evento adverso, sea o no relacionado al uso del fármaco del actual protocolo. Hasta el momento hemos notificado 7 defunciones, en el resumen de cada una de estas notificaciones se ha especificado que han sido pacientes quienes al momento de la aleatorización ya se encontraban con apoyo de ventilación mecánica. El número total de sujetos incluidos en el protocolo hasta el día de hoy es de 55.

3. La información con la que contamos actualmente sobre las defunciones son las siguientes:
   H-003: Brazo B (estratificación paciente con ventilación mecánica), fármaco lote AMXA015. Causa de defunción: Enfermedad por COVID-19.
   H-011: Brazo B (estratificación paciente con ventilación mecánica), fármaco lote AMXA015. Causa de defunción: Enfermedad por COVID-19.
   H-012: Brazo B (estratificación paciente con ventilación mecánica), fármaco lote AMXA001. Causa de defunción: Enfermedad por COVID-19.
   H-019: Brazo B (estratificación paciente con ventilación mecánica), fármaco lote AMXA015. Causa de defunción: Enfermedad por COVID-19.
   H-026: Brazo B (estratificación paciente con ventilación mecánica), fármaco lote AMXA001. Causa de defunción: Enfermedad por COVID-19.
   H-021: Brazo B (estratificación paciente con ventilación mecánica), fármaco lote AMXA015. Causa de defunción: Enfermedad por COVID-19.
   H-029: Brazo B (estratificación paciente con ventilación mecánica), fármaco lote AMXA001. Causa de defunción: Enfermedad por COVID-19.

   Brazo B, se refiere a la estratificación del sujeto por la condición de encontrarse con apoyo mecánico de la ventilación en el momento de la estratificación.
   El número de lote corresponde ya sea a placebo o a hidroxicloroquina 200 mg. (cegado). Uno de los grupos tiene 4 fallecimientos y el otro 3 todos atribuibles a COVID-19, lo cual no parece disparejo entre los grupos, y como porcentaje del total es mas bien bajo. Ignoramos por supuesto cual corresponde al tratamiento y cual al placebo.

4. En relación a los efectos adversos no serios le anexo por separado cada uno de los mismos.

La mortalidad reportada hasta febrero de este año en países Asiáticos y Europeos ha llegado a ser del 33.33% del total de casos confirmados. (1,2) Desgraciadamente el requerimiento de ventilación mecánica en los pacientes con SARS-Cov-2 incrementa el riesgo de morir de forma importante; por ejemplo; en un estudio retrospectivo realizado en China, de 191 pacientes analizados, 32 de ellos requirieron apoyo con ventilación mecánica de los cuales 31 (97%) fallecieron. (3)

En Estados Unidos de América el 27 de marzo a través de la CDC (Centers for Disease Control and Prevention, por sus siglas en inglés) se habían reportado 4,226 casos confirmados de los cuales marcadores de gravedad se documentaron necesidad de hospitalización y apoyo de unidad de cuidados intensivos. (4) El porcentaje de pacientes que requirieron apoyo mecánico ventilatorio fue 10 veces mayor que en China, y de estos la necesidad de otorgar tratamiento de reemplazo renal y uso de vasopresores se incremento de forma sustancial. (5)

En nuestro país se tiene estimada una tasa de infección del 80%, con una mortalidad estimada del 10% (6) del total de pacientes, pero de los que están en ventilación mecánica es mucho mayor, precisamente porque están más graves y podría estar entre el 30 y el 50%.

Referencias:

1. Lai C, Wang C, Wang Y, Hsueh S, Ko W, Hsueh P. Global epidemiology of coronavirus disease 2019: disease incidence, daily cumulative index, mortality, and their association with country healthcare resources and economic status. Int J Antimicrob Agents [Internet]. 2020;105946. Available from: https://doi.org/10.1016/j.ijantimicag.2020.105946
2. Notes C-. C or r e sp ondence Adaptations and Lessons in the Province of Bergamo. 2020;
3. Zhou F, Yu T, Du R, Fan G, Liu Y, Liu Z, et al. Clinical course and risk factors for mortality of adult inpatients with COVID-19 in Wuhan, China: a retrospective cohort study. Lancet. 2020;395.
4. Report MW. Severe Outcomes Among Patients with Coronavirus Disease 2019 (COVID-19) — United States , February 12 – March 16 , 2020. 2020;69(12):343–6.
5. City NY, City NY, Appendix S, March B. C or r e sp ondence Clinical Characteristics of Covid-19 in New York City. 2020;1–3.
6. Grech V. Early Human Development Unknown unknowns – COVID-19 and potential global mortality. Early Hum Dev [Internet]. 2020;144(March):105026. Available from: https://doi.org/10.1016/j.earlhumdev.2020.105026

Agradezco su atención, y quedo a sus órdenes para cualquier información adicional.

Atentamente

DRA. CARMEN MARGARITA HERNANDEZ CARDENAS
INVESTIGADOR PRINCIPAL.
JEFA DE ÁREAS CRÍTICAS

Dirección de Investigación
Subdirección de Investigación Clínica
Departamento de Investigación en Tabaquismo y EPOC

INER/DITABE/143/2020
Ciudad de México, a 14 de julio del 2020

DRA. BLANCA MARGARITA BAZAN PERKINS
PRESIDENTE DEL COMITE DE INVESTIGACIÓN

LIC. ADRIANA ESPINOSA JOVE
PRESIDENTE DEL COMITE DE ETICA EN INVESTIGACION

Por la presente hago de su conocimiento que el reclutamiento de nuevos pacientes para el proyecto *"Hidroxicloroquina para el tratamiento de infección respiratoria grave por COVID-19: ensayo clínico controlado" Número de protocolo C13-20* del que soy investigador responsable, ha sido suspendido desde hace días.

Las razones para hacerlo, incluyen el aviso casi simultáneo de detención o cancelación de varios ensayos que involucran hidroxicloroquina por ineficacia, no tanto por toxicidad o riesgos aumentados, varios de los cuales citan en su comunicado.

Si bien hasta la fecha de hoy, Julio 15, no se ha publicado formalmente en alguna revista científica revisada por pares, ninguno de los estudios referidos, situación poco habitual para detener un ensayo clínico, la evidencia que se describe es consistente, e incluye ensayos clínicos controlados con un tamaño de muestra mucho mayor que el nuestro que suman varios miles de pacientes estudiados con HCQ.

En total en nuestro estudio se han reclutado 205 pacientes (en total en los 3 centros registrados, INER, Ixtapaluca y Oaxaca), de los cuales aproximadamente 150 han terminado el mes de seguimiento y los demás se están siguiendo todavía hasta completar 30 días, lo que sucederá a principios del mes de Agosto.

En los análisis de seguridad que se han realizado sin abrir código, no hemos encontrado diferencias significativas en la mortalidad entre los 2 brazos. Esto es consistente con una ausencia significativa de daño pero también de beneficio, ya que nuestro indicador primario de efectividad es la mortalidad a 30 días. En el análisis detallado se probarán otros desenlaces adicionales.

Es importante notar, que la OMS ya había suspendido temporalmente el brazo de hidroxicloroquina previamente(5) con base en un estudio observacional extenso multicéntrico y multinacional con 96,000 pacientes, pero por problemas en la calidad de los datos, o dudas en la calidad, el artículo de la prestigiada revista Lancet, fue retractado. Con la retracción, el brazo de HCQ se reactivó.

Instituto Nacional de Enfermedades Respiratorias Ismael Cosío Villegas
Comité de Investigación
21 JUL. 2020
RECIBIDO
INER

Instituto Nacional de Enfermedades Respiratorias Ismael Cosío Villegas
Comité de Ética en Investigación
21 JUL. 2020
RECIBIDO
INER

Recientemente se publicaron 2 estudios observacionales que encuentran beneficio con la HCQ, con la desventaja de que son observacionales y sujetos a sesgos difíciles de controlar en el análisis.

PLAN ACTUAL Y ASUNTOS FALTANTES

1- Completar el seguimiento de los pacientes reclutados, incluyendo las pruebas de laboratorio y gabinete comprometidas en el protocolo, hasta terminar el período de seguimiento (30 dias).
2- Capturar y depurar la base de datos de los pacientes.
3- Hacer un reporte formal al comité científico y ético del INER.
4- Por lo mismo el personal que labora en el proyecto continuará haciéndolo hasta completar los puntos 1,2,3 en los cuales está involucrado.

REFERENCES

1. National Institutes of Health. Nih halts clinical trial of hydroxychloroquine. 2020 June 20, 2020]. Available from: https://http://www.nih.gov/news-events/news-releases/nih-halts-clinical-trial-hydroxychloroquine.
2. Randomised Evaluation of COVid-19 thERapY (RECOVERY) Trial. No clinical benefit from use of hydroxychloroquine in hospitalised patients with COVID-19. 2020. Available from: https://http://www.recoverytrial.net/news/statement-from-the-chief-investigators-of-the-randomised-evaluation-of-covid-19-therapy-recovery-trial-on-hydroxychloroquine-5-june-2020-no-clinical-benefit-from-use-of-hydroxychloroquine-in-hospitalised-patients-with-covid-19.
3. World Health Organization. Who discontinues hydroxychloroquine and lopinavir/ritonavir treatment arms for COVID-19. 2020. Available from: https://http://www.who.int/news-room/detail/04-07-2020-who-discontinues-hydroxychloroquine-and-lopinavir-ritonavir-treatment-arms-for-covid-19.
4. NOVARTIS. Novartis discontinues hydroxychloroquine clinical trial based on slow enrollment, remains committed to pandemic research efforts. 2020. Available from: https://http://www.novartis.com/news/media-releases/novartis-discontinues-hydroxychloroquine-clinical-trial-based-slow-enrollment-remains-committed-pandemic-research-efforts.
5. World Health Organization. Who halts hydroxychloroquine trial over safety concerns. 2020.

Sin más por el momento, reciban un cordial saludo.

Atentamente,

DRA. CARMEN MARGARITA HERNANDEZ CARDENAS
INVESTIGADOR PRINCIPAL

SALUD
SECRETARÍA DE SALUD
INER
INSTITUTO NACIONAL
DE ENFERMEDADES
RESPIRATORIAS
ISMAEL COSÍO VILLEGAS

Comité de Ética en Investigación
INER/CEI/287/2020
Comité de Investigación
INER/CI/265/2020

Ciudad de México, a 28 de julio de 2020

Dra. Carmen Margarita Hernández Cárdenas
Investigador Responsable
Presente

Estimada Dra. Hernández Cárdenas:

Por este medio agradecemos el escrito INER/DITABE/143/2020 en donde informa que se ha suspendido el reclutamiento de nuevos participantes para el protocolo de Investigación **"HIDROXICLOROQUINA PARA EL TRATAMIENTO DE INFECCIÓN RESPIRATORIA GRAVE POR COVID-19: ENSAYO CLÍNICO CONTROLADO"**, con número de registro **C13-20**.

Le recordamos que es responsabilidad del Investigador Principal notificar a la COFEPRIS la terminación anticipada del proyecto, para dar cumplimiento al numeral 8.8 de la NOM-012-SSA3-2012.

Quedamos en espera del informe final del proyecto.

Agradeciendo su amable atención, reciba un cordial saludo.

Atentamente

Lic. Adriana Espinosa Jove
Presidente del Comité de Ética en Investigación.

Dra. En C. Blanca M. Bazán Perkins
Presidente del Comité de Investigación.

2020
LEONA VICARIO
