# Supplementary material for: Hydroxychloroquine for the treatment of severe respiratory infection by COVID-19: A randomized controlled trial
Source: PLoS One. 2021 Sep 28;16(9):e0257238. doi: 10.1371/journal.pone.0257238 (PMC8478184; doi:10.1371/journal.pone.0257238)
Supplement: S3 File — (PDF) [file pone.0257238.s007.pdf]

MANUAL DE PROCEDIMIENTOS
HIDROXICLOROQUINA PARA EL TRATAMIENTO DE INFECCIÓN RESPIRATORIA GRAVE POR COVID-19: ENSAYO CLÍNICO CONTROLADO.

Número de aprobación por comité Institucional de Ética:

Centro Coordinador:

Instituto Nacional de Enfermedades Respiratorias Ismael Cosío Villegas

Contenidos

Introducción 2
Resumen del protocolo: 2
- Tipo de investigación 2
- Planteamiento del problema 2
- Antecedentes 3
- Justificación 3
- Hipótesis 3
- Objetivo primario y secundario 3
- Metodología 4

Personal del estudio y responsabilidades 4
Cronograma de evaluaciones 5
Reclutamiento y plan de retención 5
- Reclutamiento 5
  - Criterios de inclusión 5
  - Criterios de exclusión: 6
  - Criterios de retiro/eliminación: 6

Plan de compensación para sujetos 7
Intervención del estudio 7
Aleatorización 7
- Cegado y fin del cegado 8

Reporte de seguridad 8
- Riesgos potenciales conocidos 8
- Riesgos potenciales de la hidroxicloroquina 8
- Riesgos a la privacidad 8

Retención 9
Colección de datos y forma de reporte de caso 9
- Instrucciones generales para completar las formas de reporte de caso 9

Instrucciones generales para completar la base de datos 10
Almacenamiento de documentos de estudio 10
Manejo de datos ***10***
Procedimientos de control de calidad 10
Actividades de monitoreo de seguridad de datos y seguridad 11
Procedimientos de confidencialidad 11
Seguridad 11
Operacionalización de variables ***11***
Plan de análisis de datos detallado ***19***
Estadística descriptiva y bivariada. 19

Introducción:

El COVID–19 es un nuevo tipo de neumonía viral descrito en diciembre 2019 en Wuhan – China; representa un problema de salud pública importante por su rápida propagación y la capacidad de generar neumonía grave en pacientes susceptibles; al momento no se a descrito ningún tratamiento específico para el virus siendo su manejo principalmente el soporte vital en los casos más graves. Se describió la actividad in vitro de la cloroquina contra el virus SARS-COV pero al momento no se cuenta con evidencia de que este tratamiento ni su derivado hidroxicloroquina pueda disminuir la presencia de desenlaces clínicos de interés (requerimiento de soporte ventilatorio invasivo, mortalidad, tiempo de asistencia ventilatoria mecánica invasiva y tiempo de hospitalización) en sujetos con enfermedad grave por COVID – 19; el objetivo del ensayo es estimar si es que el tratamiento con hidroxicloroquina comparado con placebo en enfermedad pulmonar grave por COVID – 19 puede disminuir la mortalidad intrahospitalaria.

Resumen del protocolo:

Tipo de investigación

Ensayo clínico controlado y aleatorizado doble ciego diseñado para evaluar la seguridad y eficacia de la hidroxicloroquina para el tratamiento de la enfermedad grave por COVID – 19.

Planteamiento del problema

El COVID – 19 es un nuevo tipo de neumonía viral descrito en diciembre 2019 en Wuhan – China; representa un problema de salud público

importante por su rápida propagación y la capacidad de generar neumonía grave en pacientes susceptibles; al momento no se a descrito ningún tratamiento específico para el virus siendo su manejo principalmente el soporte.

Antecedentes El brote de infección respiratoria por el coronavirus 2019 (2019-nCOV) inició en China en diciembre 2019, en los últimos días el brote se ha ido extendiendo a otros países llegando a declararse el riesgo de una epidemia internacional. Hasta el momento no se cuenta con un tratamiento efectivo disponible para el COVID - 19, sin embargo, in vitro se reportó la efectividad de la cloroquina en concentraciones capaces de ser obtenidas in vivo en pacientes que la usan. La cloroquina e hidroxicloroquina, son medicamentos originalmente antimaláricos, en uso regular por décadas y con una toxicidad limitada, a un costo muy bajo, y en caso de ser efectivo in vivo, podría ser un medicamento de gran utilidad tanto en países desarrollados como en desarrollo. El efecto antiviral amplio, se ha atribuido al incremento del pH de los endosomas, que se requieren para la fusión del virus con la célula y también por interferencia con la glicosilación de los receptores celulares del SARS-COV.

La tasa de letalidad general de la infección por COVID - 19 se ha estimado alrededor del 2%, pero en los casos graves en falla respiratoria, o requiriendo cuidados críticos, es considerablemente mayor, entre 10-15%, y fácilmente podría incrementarse hasta el 20-30% dependiendo de los criterios de entrada a una unidad de terapia intensiva y la disponibilidad de unidades especializadas.

Justificación Se carece de un tratamiento farmacológico aprobado para el COVID - 19, y basados en la efectividad in Vitro de la cloroquina, su seguridad demostrada por décadas, su costo bajo, se propone un ECC, doble ciego, para evaluar la eficacia y seguridad de la hidroxicloroquina en enfermos hospitalizados con enfermedad respiratoria grave por COVID - 19.

Hipótesis El tratamiento con hidroxicloroquina 400 mg/día por 10 días comparado con placebo reduce mortalidad intrahospitalaria en sujetos con enfermedad respiratoria grave por COVID – 19.

Objetivo primario y secundario Estimar si el tratamiento por 10 días con 400 mg/día de hidroxicloroquina comparada con placebo reduce mortalidad intrahospitalaria, medida a los 30 dias. Los desenlaces secundarios serán días de ventilación mecánica,

requerimiento de soporte ventilatorio mecánico, días de hospitalización y la incidencia acumulada de eventos adversos serios y eventos adversos grado 3 y 4.

Metodología Ensayo clínico aleatorizado doble ciego. Se incluirá a sujetos mayores de 18 años con diagnóstico de enfermedad grave de COVID-19 confirmada por RT-PCR y enfermedad grave que acepten participar. La aleatorización será realizada en software dedicado por personal no involucrado en el tratamiento, diseño ni análisis del estudio. Se estratificarán a los sujetos en dos grupos (sujetos que requieran hospitalización y sujetos que requieran soporte ventilatorio al momento de la aleatorización). Se realizará el análisis en STATA y en software estadístico R-Rstudio. Se compararán los desenlaces principales mediante pruebas paramétricas y no paramétricas de acuerdo al tipo de variable y distribución. Se Calcula el tamaño de muestra de acuerdo a la mortalidad en la población con enfermedad grave por COVID – 19 estimándose una muestra de 500 sujetos.

Personal del estudio y responsabilidades

Nombre	Afiliación	Institución	Responsabilidades
Dra Carmen Hernandez Cárdenas	Unidad de Cuidados Intensivos Respiratorios	INER	Investigador Principal
Dr. Cristobal Guadarrama	Unidad de Urgencias Respiratorias	INER	Subinvestigador
Dr. Rogelio Perez Padilla	Departamento de Investigación en Tabaquismo y EPOC	INER	Subinvestigador
Dr. Joel Armando Vasquez	Departamento de Investigación en Tabaquismo y EPOC	INER	Subinvestigador
Dra Ireri Isadora Thirion Romero	Departamento de Investigación en Tabaquismo y EPOC	INER	Subinvestigador
Dr. Luis Felipe Jurado Camacho	Unidad de Cuidados Intensivos Respiratorios	INER	Subinvestigador
Dra Arantxa	Departamento de Investigación en Tabaquismo y EPOC	INER	Coordinadora Institucional

Cronograma de evaluaciones

	CRIBADO	TIEMPO BASAL	DIARIO HASTA DÍA 10	DIARIO HASTA ALTA HOSPITALARIA
**VENTANA EN DÍAS +/-**	-1 o 1	1		
**CRIBADO**				
Elegibilidad	X			
Verificar resultado de sars-cov-2	X			
Consentimiento informado	X			
Toma de mediciones basales		X		
**INTERVENCION**				
Aleatorización		x		
Administracion de hidroxicloroquina/placebo			Diario hasta el día 10	
Procedimientos del estudio				
Registro de po2 y fio2				Diario hasta el alta hospitalaria

Reclutamiento y plan de retención

Reclutamiento

- Se anticipa que los sujetos con COVID-19 se presentarán a los centros participantes; por tanto, no se realizarán otros esfuerzos para reclutar a los sujetos necesarios.
- Se diseminará información acerca del ensayo a otros centros y profesionales médicos.
- Se evaluará para elegibilidad a todos los sujetos que ingresen a urgencias con sospecha de enfermedad grave por COVID-19; se aleatorizará e iniciará la intervención sólo tras la firma del consentimiento informado.

Criterios de inclusión

1. Firma de Consentimiento informado por el sujeto (o representante legal) entendiendo y aceptando los procedimientos y medicación del estudio descritos a continuación.
    a. Aleatorización a hidroxocloroquina o placebo idéntico.

b. Recolección de muestra orofaringea y muestras sanguíneas para confirmación y seguimiento de COVID – 19.
c. Hospitalización o ingreso a unidad de cuidados intensivos.

2. En caso de ser mujer prueba de embarazo negativa.
3. Al menos 18 años cumplidos al momento de la aleatorización.
4. Confirmación por laboratorio de infección por SARS-CoV-2 determinada por RT-PCR de muestra faríngea, nasofaríngea, lavado broncoalveolar o aspirado traqueal antes de la aleatorización.
5. Enfermedad grave por COVID – 19 definida por al menos uno de los siguientes:
    a. SpO2 menor o igual a 90 % a una altura de 2240 m, en el valle de México o Puebla o disminución de más del 3% de saturación basal
    b. Requiere ventilación mecánica y/o oxígeno suplementario (o incremento del apoyo de oxigeno suplementario en hipoxemia crónica)
    c. Cumple criterios de sepsis/choque séptico.

Criterios de exclusión:

1. Mayor de 80 años.
2. Hipersensibilidad nueva o conocida a hidroxicloroquina manifestada por anafilaxia.
3. Consumo previo a la hospitalización de hidroxicloroquina o cloroquina
4. Decisión del médico responsable de eliminarlo del estudio.
5. Transferencia del paciente a otra unidad hospitalaria.
6. Antecedente de enfermedad hepática crónica. (Child-Pugh B o C)
7. Antecedente de enfermedad renal crónica. (TFG menor o igual a 30)

Criterios de retiro/eliminación:

1. Decisión del médico responsable de retirar al participante del estudio.
2. Transferencia del paciente a otra unidad hospitalaria.
3. Hipersensibilidad nueva a hidroxicloroquina manifestada por anafilaxia.
4. Efecto adverso grado 3 o 4
5. Decisión del participante o representante de retirarse del estudio.

Plan de compensación para sujetos

El INER en su carácter de Patrocinador de la investigación vigilará el seguimiento de los participantes y la presencia de reacciones adversas; dando seguimiento clínico a los sujetos afectados.

Intervención del estudio

Los sujetos serán aleatorizados tras la firma del consentimiento informado a uno de los siguientes grupos:

- Grupo intervención: Hidroxicloroquina 200mg VO o por sonda nasogástrica/orogástrica cada 12 horas por 10 días. En pacientes que no toleren la vía oral se administrará la tableta tras el procedimiento estándar de administración por sonda.
- Grupo control: placebo idéntico, 1 tableta VO o por sonda nasogástrica/orogástrica cada 12 horas por 10 días. En pacientes que no toleren la vía oral se administrará la tableta tras el procedimiento estándar de administración por sonda.

Nota: Para administrar la tableta por sonda oro/nasogástrica se procederá a molerla en mortero, se mezcla con 10cc de agua destilada y se la administra con una jeringa; tras la administración se lavará la sonda orogástrica con 20 cc de agua destilada.

Aleatorización

Los sujetos serán aleatorizados tras la firma de consentimiento informado al grupo intervención o placebo; el médico tratante se comunicará vía telefónica al COORDINADOR INSTITUCIONAL quien registrará el estrato correspondiente e indicará el número de sobre que contiene la medicación par administración subsecuente. Sólo el COORDINADOR INSTITUCIONAL tendrá la lista de aleatorización con los códigos de los sujetos.

La aleatorización será estratificada por:

1. Centro reclutador
2. Gravedad de la enfermedad:

a. COVID-19 crítica: requiere ventilación mecánica invasiva o no invasiva.
b. COVID-19 grave con necesidad de hospitalización: SpO2 menor a 90% a aire ambiente o caída de 3% de la SpO2 basal o incremento de los requerimientos de oxígeno, taquipnea (>25 rpm)

Cegado y fin del cegado

El grupo al que pertenece el sujeto será cegado a los médicos y personal de enfermería tratante y al analista de datos.

Se finalizará el cegado en caso de que el médico tratante indique la necesidad de retirar del estudio o se cumplan otros criterios de retiro/eliminación.

Reporte de seguridad

Riesgos potenciales conocidos

Los riesgos potenciales asociados en este estudio incluyen posibles reacciones adversas a hidroxicloroquina y fallas en el mantenimiento de la confidencialidad.

Riesgos potenciales de la hidroxicloroquina

La hidroxicloroquina es un agente terapéutico conocido y relativamente seguro; algunos sujetos pueden experimentar molestias abdominales manifestadas por nausea, vómitos y/o diarrea; efectos leves de resolución espontánea al terminar el tratamiento o con medidas sintomática.

En algunos casos se encuentra retinopatía progresiva; (frecuencia de 1% al 10% tras cinco años de consumo y con dosis mayores a 6mg/Kg/día); se limita la frecuencia de esta complicación al no superar una dosis de 400mg/día y el tratamiento por 10 días. Se produce debido a la unión a melanina y depósito en la retina. Se monitorizará la presencia de reacciones adversas graves y grado 3 y 4 durante la duración del tratamiento y hasta el alta hospitalaria.

Riesgos a la privacidad

Los sujetos proveerán información personal relacionada con su estado de salud y antecedentes patológicos y no patológicos; se intentará mantener esta información confidencial dentro de los límites de la ley; de todas formas, existe una pequeña probabilidad de que personas no autorizadas vean los datos del sujeto. Todos los archivos y evaluaciones serán almacenadas en un gabinete bajo llave y dentro una

oficina cerrada con llave en el centro. Los archivos electrónicos serán protegidos mediante password.

Solo las personas involucradas en la planificación, supervisión, monitoreo o auditorias del estudio tendrán acceso a la información colectada.

Toda publicación de este ensayo utilizará información de-identificada; organizaciones que podrían inspeccionar y/o copiar la información de la investigación para evaluación de la calidad y el análisis de datos se limitan a grupos como el Comité de ética e investigación institucional, sponsor y otras autoridades regulatorias pertinentes.

Retención

Al incluir en el estudio sólo sujetos que requieran hospitalización no se esperan pérdidas más que cuando se solicite alta por parte de los familiares o cumplan criterios de retiro/eliminación.

Colección de datos y forma de reporte de caso

Instrucciones generales para completar las formas de reporte de caso

- La Forma de Reporte de Caso debe ser completada para todos los participantes que acepten y firmen el consentimiento informado.
- A excepción de la hoja correspondiente a “INFORMACION DE CONTACTO” ninguna hoja de la Forma de Reporte de Caso debe llevar el nombre ni otros datos que identifiquen al participante.
- La hoja correspondiente a “INFORMACION DE CONTACTO” debe ser resguardada bajo llave por separado del resto de la forma.
- Llenar todas las entradas con mayúsculas.
- Todo el texto y comentarios deben ser breves; en caso de que falte espacio se puede usar el reverso de la hoja; todo comentario debe ir fechado y con nombre y firma del responsable.
- Responder todas las preguntas explícitamente.
- No dejar preguntas sin responder. Si la respuesta no se conoce escribir “D”(Desconoce). Si no se realizó “NR” (No se realizó). Si la pregunta no es aplicable escribir “NA” (No aplicable).
- Si se requiere una selección tachar con X.

- Todas las entradas de fecha deben aparecer en el formato DD—MMM-AAAA, en caso de que se desconozca la fecha exacta de un evento debe anotarse DD.
- Las horas deben llenarse en un formato de 24-horas; las entradas de media noche se anotan 00:00.
- En caso de errores:
    - No escriba encima, no use líquido corrector ni borradores.
    - Tache el dato erróneo sin obliterarlo.
    - Claramente corrija el dato erróneo al lado de la entrada incorrecta.
    - Coloque la fecha y las iniciales de la persona que hace la corrección.

Instrucciones generales para completar la base de datos

- La base de datos será llenada en Redcap o en un programa de base de datos; deben transcribirse los datos tal y como están en la forma de recolección de datos.
- Fechas en formato mes/día/año.
- No usar mayúsculas ni espacios; los espacios deben ser remplazados por _.
- En caso de no contar con algún dato llenar con NA.

Almacenamiento de documentos de estudio

Las CRF serán almacenadas en el centro coordinador bajo llave; con acceso sólo al coordinador del centro e investigador principal hasta la conclusión del estudio.

Manejo de datos

Procedimientos de control de calidad

Los datos serán transcritos de los CRF a la base de datos y cuando completa y limpia se transferirá a STATA o al programa de análisis; tendrá mensajes de validación y errores de transcripción activados.

Se verificará aleatoriamente el 20% de las CRF con el expediente de cada sujeto buscando errores en el llenado de las CRF, se reportará el porcentaje de CRF con errores.

Actividades de monitoreo de seguridad de datos y seguridad

Procedimientos de confidencialidad

La base de datos será llenada con un código por sujeto; se eliminarán datos que identifiquen a los sujetos como numero de expediente, nombre y fecha de ingreso a la institución.

Seguridad

EL equipo del protocolo revisará de forma cegada datos de eventos adversos grado 3 o 4 y eventos adversos graves cada día por 10 días y el momento del alta hospitalaria. Si se encuentra un numero preocupante de eventos adversos inesperados o las muertes están disparejas en los grupos aleatorizados, se solicitará al Comité de Ética e Investigación que revise los datos sin cegamiento en una reunión ad hoc.

El Comité de Ética e Investigación solicitará información o reuniones ad hoc extra si existen otras preocupaciones acerca de la seguridad. El estudio no parará el reclutamiento esperando las revisiones del Comité de Ética e Investigación, aunque el DSMB puede recomendar suspensión temporal o permanente del reclutamiento en base a las revisiones de seguridad.

Operacionalización de variables

Variable	Periodo	Definición	Código
**Criterios de inclusión**	Aleatorización	Características que se deben tener para participar en el estudio.	1: Cumple todos<br>2: No los cumple
**Fecha de ingreso a urgencias**	Aleatorización	Fecha de hospitalización a urgencias	Fecha (Dd/Mm/Aa)<br>Hora (Hh:Mm)
**Edad**	Aleatorización	Edad en años cumplidos	Años
**Genero**	Aleatorización	Género reportado por sujeto o familiares	1: Masculino<br>0: Femenino
**Fecha de inclusión**	Aleatorización	Fecha de reclutamiento a protocolo	Fecha (Dd/Mm/Aa)
**Días de inicio de sintomatología**	Aleatorización	Días cumplidos desde el inicio de síntomas reportado por el sujeto o familiares.	1, 2, 3, etc…
**Comorbilidades**	Aleatorización	Presencia de comorbilidades reportada por sujeto o familiares.<br><br>Se reportan hasta tres comorbilidades.	0: ninguna<br>1: Respiratoria crónica<br>2: Cardiovascular<br>3: Renal<br>4: Metabólica<br>5: otras (anotar)
**Ventilación no**	Aleatorización	Uso de asistencia ventilatoria	0: No

**invasiva**		no invasiva.	1: Si (BiPAP/CPAP)<br>2: Si (Puntas de alto flujo)
**Fecha de inicio de VNI**	Aleatorización	Fecha de inicio de ventilación no invasiva	Fecha (Dd/Mm/Aa)
**Ventilación invasiva**	Aleatorización	Uso de asistencia ventilatoria invasiva	0: No<br>1: Si
**Fecha de intubación orotraqueal**	Aleatorización	Fecha en que se realizó la intubación orotraqueal	Fecha (Dd/Mm/Aa)
**Uso de aminas**	Aleatorización	Uso de aminas vasoactivas (dopamina, norepinefrina o adrenalina)	0: No<br>1: Si
**Lactato**	-Aleatorización<br>-Seguimiento día 2 a día 10.<br>-Día de conclusión (alta o fallecimiento).	Forma ionizada del lactato. Un intermediario normal en la fermentación (oxidación, metabolismo) del carbohidratos.	mmol/L
**PaO2**	-Aleatorización<br>-Seguimiento día 2 a día 10.<br>-Día de conclusión (alta o fallecimiento).	Presión arterial de oxígeno	mmHg
**FiO2**	-Aleatorización<br>-Seguimiento día 2 a día 10.<br>-Día de conclusión (alta o fallecimiento).	Fracción inspiratoria de oxígeno	10^9/L
**Pco2**	-Aleatorización<br>-Seguimiento día 2 a día 10.<br>-Día de conclusión (alta o fallecimiento).	Presión parcial de CO2	mmHg
**Ph**	-Aleatorización<br>-Seguimiento día 2 a día 10.<br>-Día de conclusión (alta o fallecimiento).	Concentración de hidrogeniones	Unidades

**Leucocitos**	-Aleatorización<br>-Seguimiento día 2 a día 10.<br>-Día de conclusión (alta o fallecimiento).	Células nucleadas en sangre	$10^3/mm^3$
**Plaquetas**	-Aleatorización<br>-Seguimiento día 2 a día 10.<br>-Día de conclusión (alta o fallecimiento).	Las células en forma de disco no nucleadas se formaron en el megacariocito y se encuentran en la sangre de todos los mamíferos. Están involucrados principalmente en la coagulación de la sangre.	$10^9/L$
**Creatinina**	-Aleatorización<br>-Seguimiento día 2 a día 10.<br>-Día de conclusión (alta o fallecimiento).	Producto resultante del catabolismo muscular.	mg/dL
**Potasio**	-Aleatorización<br>-Seguimiento día 2 a día 10.<br>-Día de conclusión (alta o fallecimiento).	Catión principal en el líquido intracelular del músculo y otras células.	mEq/L
**Neutrofilos**	-Aleatorización<br>-Seguimiento día 2 a día 10.<br>-Día de conclusión (alta o fallecimiento).	Leucocitos granulares que tienen un núcleo con tres a cinco lóbulos conectados por hilos delgados de cromatina, y citoplasma que contiene gránulos finos discretos y se puede teñir con tintes neutros.	$/10^9/L$
**Eosinofilos**	-Aleatorización<br>-Seguimiento día 2 a día 10.<br>-Día de conclusión (alta o fallecimiento).	Leucocitos granulares con un núcleo que generalmente tiene dos lóbulos conectados por un hilo delgado de cromatina y citoplasma que contiene gránulos gruesos y redondos de tamaño uniforme y manchables por la eosina.	$10^9/L$
**BUN**	-Aleatorización<br>-Seguimiento día 2 a día 10.<br>-Día de	Producto final del catabolismo proteico.	mg/dL

	conclusión (alta o fallecimiento).		
**TP**	-Aleatorización<br>-Seguimiento día 2 a día 10.<br>-Día de conclusión (alta o fallecimiento).	Determinación del tiempo de coagulación del plasma citratado, tras la adición de un exceso de tromboplastina tisular y calcio. Sirve para medir la vía extrínseca de la coagulación, así como la vía común	S
**INR**	-Aleatorización<br>-Seguimiento día 2 a día 10.<br>-Día de conclusión (alta o fallecimiento).	Sistema establecido por la Organización Mundial de la Salud y Comité internacional en trombosis y hemostasia para monitorear e informar las pruebas de coagulación sanguínea. Bajo este sistema, los resultados se estandarizan usando el Índice de sensibilidad internacional para la combinación particular de reactivo de prueba / instrumento utilizado.	Unidades
**TTPA**	-Aleatorización<br>-Seguimiento día 2 a día 10.<br>-Día de conclusión (alta o fallecimiento).	Consiste en medir el tiempo de coagulación del plasma citratado, en contacto con calcio y fosfolípidos (cefalina). Mide la vía intrínseca de la coagulación y la vía común.	segundos
**Sodio**	-Aleatorización<br>-Seguimiento día 2 a día 10.<br>-Día de conclusión (alta o fallecimiento).	Determinante primario de la osmolaridad plasmática y contribuye a la regulación del volumen extracelular	mEq/L
**Bilirrubina directa**	-Aleatorización<br>-Seguimiento día 2 a día 10.<br>-Día de conclusión (alta o fallecimiento).	Bilirrubina conjugada por el hígado, está unida a ácido glucorónico.	mg/dL
**Bilirrubina total**	-Aleatorización	Suma de bilirrubina indirecta y	mg/dL

	-Seguimiento día 2 a día 10.<br>-Día de conclusión (alta o fallecimiento).	directa.	
**AST**	-Aleatorización<br>-Seguimiento día 2 a día 10.<br>-Día de conclusión (alta o fallecimiento).	Miembro de un grupo de enzimas que catalizan la transferencia de un grupo amino de un aminoácido a un cetoácido, dando lugar a la formación de un nuevo aminoácido y dejando como residuo un nuevo tipo de cetoácido. AST o TGO.	UI/L
**ALT**	-Aleatorización<br>-Seguimiento día 2 a día 10.<br>-Día de conclusión (alta o fallecimiento).	Son enzimas que transforman proteínas en ATP para las células hepáticas. Alaninoaminotransferasa o transaminasa glutámico-pirúvica (ALT TGP)	UI/L
**Volumen tidal espiratorio**	-Aleatorización<br>-Seguimiento día 2 a día 10.<br>-Día de conclusión (alta o fallecimiento).	Volumen espiratorio que queda al final del volumen corriente.	ml
**PEEP**	-Aleatorización<br>-Seguimiento día 2 a día 10.<br>-Día de conclusión (alta o fallecimiento).	Presión supratmosferica aplicada por el ventilador al final de la espiración	cmH2O
**Frecuencia respiratoria**	-Aleatorización<br>-Seguimiento día 2 a día 10.<br>-Día de conclusión (alta o fallecimiento).	Número de respiraciones durante un periodo de tiempo	Rpm
**Presión meseta**	-Aleatorización<br>-Seguimiento día 2 a día 10.<br>-Día de	Presión a los dos segundos de una maniobra de pausa inspiratoria. Verificar flujo cero.	cmH2O

	conclusión (alta o fallecimiento).		
**Presión media**	-Aleatorización<br>-Seguimiento día 2 a día 10.<br>-Día de conclusión (alta o fallecimiento).	Presión promedio durante un ciclo respiratorio completo.	CmH2O
**Efectos adversos**	-Aleatorización<br>-Seguimiento día 2 a día 10.<br>-Día de conclusión (alta o fallecimiento).	Cualquier otra respuesta del fármaco que es nociva, no intencionada y que se produce a dosis habituales para la profilaxis, diagnóstico o tratamiento.	0: No<br>1: Si
**Efectos adversos visuales**	-Aleatorización<br>-Seguimiento día 2 a día 10.<br>-Día de conclusión (alta o fallecimiento).	Cualquier respuesta del sistema ocular al fármaco que es nociva, no intencionada y que se produce a dosis habituales para la profilaxis, diagnóstico o tratamiento.	0: No<br>1: Si<br>Pérdida de visión<br>Otro____
**Efectos adversos cardiovasculares**	-Aleatorización<br>-Seguimiento día 2 a día 10.<br>-Día de conclusión (alta o fallecimiento).	Cualquier respuesta del sistema cardiovascular al fármaco que es nociva, no intencionada y que se produce a dosis habituales para la profilaxis, diagnóstico o tratamiento.	0: No<br>1: Si<br>Hipotensión<br>QTc prolongado<br>Depresión onda T<br>Otro___
**Efectos adversos neurológicos**	-Aleatorización<br>-Seguimiento día 2 a día 10.<br>-Día de conclusión (alta o fallecimiento).	Cualquier respuesta del sistema neurológico al fármaco que es nociva, no intencionada y que se produce a dosis habituales para la profilaxis, diagnóstico o tratamiento.	0: No<br>1: Si<br>Confusión<br>Mareo<br>Cefalea<br>Parestesias<br>Convulsiones<br>Neuropatía<br>Otros____
**Efectos adversos hepatobiliar**	-Aleatorización<br>-Seguimiento día 2 a día 10.<br>-Día de conclusión (alta o fallecimiento).	Cualquier respuesta del sistema hepatobiliar al fármaco que es nociva, no intencionada y que se produce a dosis habituales para la profilaxis, diagnóstico o tratamiento.	0: No<br>1: Si<br>Elevación de transaminasas

**Efectos adversos gastrointestinales**	-Aleatorización<br>-Seguimiento día 2 a día 10.<br>-Día de conclusión (alta o fallecimiento).	Cualquier respuesta del sistema gastrointestinal al fármaco que es nociva, no intencionada y que se produce a dosis habituales para la profilaxis, diagnóstico o tratamiento.	0: No<br>1: Si<br>Vomito/nausea<br>Diarrea<br>Otro____
**Efectos adversos dermatológicos**	-Aleatorización<br>-Seguimiento día 2 a día 10.<br>-Día de conclusión (alta o fallecimiento).	Cualquier respuesta dermatológica al fármaco que es nociva, no intencionada y que se produce a dosis habituales para la profilaxis, diagnóstico o tratamiento.	0: No<br>1: Si<br>Prurito<br>Alopecia<br>Eritema/urticaria<br>Otro___
**Efectos adversos hematológicos**	-Aleatorización<br>-Seguimiento día 2 a día 10.<br>-Día de conclusión (alta o fallecimiento).	Cualquier respuesta hematológica al fármaco que es nociva, no intencionada y que se produce a dosis habituales para la profilaxis, diagnóstico o tratamiento.	0: No<br>1: Si<br>Agranulocitosis/trombocitopenia<br>Pancitopenia<br>Otro_____
**Efectos adversos auditivos**	-Aleatorización<br>-Seguimiento día 2 a día 10.<br>-Día de conclusión (alta o fallecimiento).	Cualquier respuesta del sistema auditivo al fármaco que es nociva, no intencionada y que se produce a dosis habituales para la profilaxis, diagnóstico o tratamiento.	0: No<br>1: Si<br>Tinitus<br>Pérdida de audición<br>Otro____
**Efectos adversos otros**	-Aleatorización<br>-Seguimiento día 2 a día 10.<br>-Día de conclusión (alta o fallecimiento).	Cualquier otra respuesta del fármaco que es nociva, no intencionada y que se produce a dosis habituales para la profilaxis, diagnóstico o tratamiento.	0: No<br>1: Si<br>_________
**Detección de reacciones adversas grado 3 o 4**	-Aleatorización<br>-Seguimiento día 2 a día 10.<br>-Día de conclusión (alta o fallecimiento).	Detecciones de efectos adversos grado 3 o 4.	0: No<br>1: Si
**Reacciones adversas Grado 3 (Grave)**	-Aleatorización<br>-Seguimiento día 2 a día 10.<br>-Día de conclusión	El resultado para el paciente es sintomático y exige una intervención quirúrgica o médica mayor, acorta la esperanza de vida o causa un	0: No<br>1: Si

	(alta o fallecimiento).	daño o una pérdida funcional importante y permanente o de largo duración.<br>Especificar_______	
**Reacciones adversas Grado 4 (Muerte)**	-Aleatorización<br>-Seguimiento día 2 a día 10.<br>-Día de conclusión (alta o fallecimiento).	Sopesando las probabilidades, el incidente causó la muerte o la propicio a corto plazo<br>Especificar___________	0: No<br>1: Si
**Administración fármaco**	Desenlace	Se administró el medicamento durante 10 días	0: Si<br>1: No
**Fecha de alta hospitalaria o fallecimiento**	Desenlace	Si fallece o se va de alta, fecha en la que ocurre el evento	0: Fallece<br>1: Alta<br>Fecha (Dd/Mm/Aa)
**Días totales de hospitalización**	Desenlace	Días cumplidos desde el inicio desde la aleatorización hasta el alta hospitalaria o fallecimiento	1, 2, 3, etc...
**Días totales de asistencia ventilatoria mecánica invasiva**	Desenlace	Días cumplidos desde el inicio desde la aleatorización hasta el alta hospitalaria o fallecimiento	1, 2, 3, etc.
**Ventilación no invasiva**	Desenlace	Uso de asistencia ventilatoria no invasiva.	0: No<br>1: Si (BiPAP/CPAP)<br>2: Si (Puntas de alto flujo)
**Fecha de inicio de ventilación no invasiva**		Fecha en la que se inició el uso de asistencia ventilatoria no invasiva	Fecha (Dd/Mm/Aa)
**Fecha de término de ventilación no invasiva**		Fecha en la que se suspendió el uso de asistencia ventilatoria no invasiva	Fecha (Dd/Mm/Aa)
**Ventilación invasiva**	Desenlace	Uso de asistencia ventilatoria invasiva	0: No<br>1: Si
**Fecha de inicio de ventilación invasiva**	-	Fecha en la que se inició el uso de asistencia ventilatoria invasiva	Fecha (Dd/Mm/Aa)
**Fecha de término de ventilación invasiva**	-	Fecha en la que se suspendió el uso de asistencia ventilatoria invasiva<br>Desconexión de AVM si traqueostomizado	Fecha (Dd/Mm/Aa)

Plan de análisis de datos

El análisis estadístico se realizará software estadístico STATA o R-R studio utilizando la última actualización disponible al momento del análisis de datos. Será realizado de forma cegada al tratamiento recibido por los individuos y tras de-identificación de los datos.

Tamaño de muestra

A) **TAMAÑO DE MUESTRA**

La mortalidad reportada en el COVID-19 grave se encuentra entre el 8% a incluso al 60% dependiendo la fuente y la OMS sugiere al menos 400 sujetos para la estimación de la muestra en ensayos clínicos que evalúen terapias para el COVD-19 (3). Al momento se desconocen muchas características del comportamiento y desenlaces del COVID-19 especialmente en nuestro medio; por eso utilizamos la estimación de una mortalidad alrededor del 15% en COVID-19 grave con una reducción del 50% de la mortalidad con el tratamiento obteniendo en el cálculo de muestra un número de sujetos similar al recomendado por la OMS.

Nivel de significancia de 2 colas 95, alfa 5%

Potencia (1-beta, posibilidad de detección): 80%

Tratados vs controles 1:1

Mortalidad estimada en controles 15%

Mortalidad estimada en experimentales 7.5%, reducción de mortalidad de 7.5%, que es el 50% (RR 0.5).

<u>Estimaciones (Open-Epi)</u>

	Kelsey		Fleiss	Fleiss with CC
Muestra experimental		280	279	305
Muestra control		280	279	305
Muestra total :	560	558	610	

Se decide incluir 600 sujetos para el ensayo clínico, es decir 300 sujetos en el grupo de tratamiento experimental y 300 sujetos en el grupo control.

Estadística descriptiva y bivariada.

Se utilizará estadística descriptiva de acuerdo al tipo de variable y distribución. Se comparará la mortalidad entre el grupo placebo e intervención mediante prueba exacta de fisher. Se compararán tiempos a los desenlaces y supervivencia global y por grupo ajustando por variables confusoras (edad, tiempo del inicio de síntomas al inicio de tratamiento, comorbilidades) mediante modelo de riesgos proporcionales de cox.

Análisis:

- La tasa de mortalidad (30 días), la proporción de pacientes que necesitan ventilación mecánica y los días de ventilación mecánica, se compararán en un

modelo multivariado, que incluye un indicador de estratos de aleatorización, ajustando las variables de confusión (edad, tiempo desde el inicio de los síntomas hasta el inicio de tratamiento, comorbilidades), se realizará una comparación similar para los resultados secundarios: duración de la hospitalización, duración de la oxigenoterapia.

- También compararemos el tiempo hasta la muerte, en forma cruda y ajustando las variables de confusión (edad, tiempo desde el inicio de los síntomas hasta el inicio del tratamiento, comorbilidades) utilizando el modelo de riesgos proporcionales de Cox. El mismo modelo se utilizará para estimar el tiempo a egreso y el tiempo a mejoría.
-
-
- Habrá un Comité de eventos adversos y evaluación de riesgos y análisis intermedio, un grupo asesor multidisciplinario, compuesto por un experto en estadística, un investigador clínico, un experto en ética y un clínico relacionado con la influenza y el coronavirus, ninguno involucrado en el ensayo clínico y, por lo tanto, independiente, que en el medio de los ensayos tendrá acceso a los datos y al código de aleatorización para evaluar los datos, incluida la seguridad y los resultados.

Investigador clínico: Gustavo Lugo, farmacólogo clínico, anestesiólogo e intensivista que actuará como coordinador. Dra. Angélica Portillo, especialista en oído, nariz y garganta y con una maestría en ciencias médicas.

Estadísticas: Dra. Rosario Fernández, Jefa del Departamento de Epidemiología del INER.

Ética: Dr. Patricio Santillán, director médico de INER.

Clínico: Dr. Arturo Martínez, Infectólogo, experto en infecciones similares a la gripe y ahora en infección por COVID-19.

CONSENTIMIENTO INFORMADO PARA PARTICIPAR EN UN ESTUDIO DE INVESTIGACIÓN MÉDICA

Título del estudio: "*Hidroxicloroquina para el tratamiento de infección respiratoria grave por COVID-19: ensayo clínico controlado".*

Investigador Principal: Dra. Carmen Margarita Hernández Cárdenas.

Sede dónde se realizará el estudio: Instituto nacional de Enfermedades Respiratorias Ismael Cosío Villegas

Nombre del participante: ____

A usted se le está invitando a participar en este estudio de investigación médica. Antes de decidir si acepta o no, debe conocer y comprender cada uno de los siguientes apartados. Este proceso se conoce como consentimiento informado. Siéntase con absoluta libertad para preguntar sobre cualquier aspecto que le ayude a aclarar sus dudas al respecto.

¨El presente estudio tiene el objetivo de evaluar si la hidroxicloroquina es un tratamiento ***eficaz y seguro*** *en pacientes con enfermedad pulmonar grave por COVID -19 (también conocido como coronavirus)".*

"Al momento no se cuenta con un tratamiento para esta enfermedad por lo que estudiar la hidroxicloroquina podría ser beneficioso para usted y otros sujetos con enfermedad pulmonar grave por COVID-19 o coronavirus¨.

"Los beneficios que esperan encontrarse con la hidroxicloroquina en el tratamiento de la enfermedad pulmonar grave por COVID-19 o coronavirus son los siguientes: menor riesgo de morir por complicaciones, menor tiempo de internamiento en el hospital, menor número de ***complicaciones pulmonares graves****"*

Nota: "¿Qué son las ***complicaciones pulmonares graves****?: Cuando una persona sufre de una enfermedad respiratoria grave como el COVID-19 o coronavirus sus pulmones pueden dañarse tanto que no son capaces de proveer oxigeno a la sangre; cuando el nivel de oxígeno en la sangre llega a niveles muy bajos es necesario remplazar el funcionamiento de los pulmones con aparatos conocidos como* ***ventiladores mecánicos****; esta es una complicación muy grave de la enfermedad donde el riesgo de morir es mucho más elevado".*

"Su participación en el estudio, las pruebas de laboratorio y tratamiento serán gratuitos y la información obtenida será confidencial".

CONSENTIMIENTO INFORMADO PARA PARTICIPAR EN UN ESTUDIO DE INVESTIGACIÓN MÉDICA

Título del estudio: "*Hidroxicloroquina para el tratamiento de infección respiratoria grave por COVID-19: ensayo clínico controlado".*

Una vez que haya comprendido el estudio y si usted desea participar se le pedirá que firme esta carta de consentimiento, de la cual se le entregará una copia firmada y fechada. **Cabe destacar que la participación en este estudio o la negación a**

participar no intervendrá de ninguna forma en su tratamiento y seguimiento y que cualquier momento puede decidir dejar de participar en el estudio.
Su participación consiste en:

"Si usted desea participar voluntariamente y firma esta carta de consentimiento procederemos a asignarle a uno de dos grupos al ***azar****; el primer grupo recibirá* ***hidroxicloroquina*** *400mg cada 24 horas por vía oral durante 10 días y el segundo grupo recibirá un* ***placebo*** *idéntico cada 24 horas por 10 días"*

Nota: ¿Que es el ***azar*** *y por qué se asigna quien recibirá el tratamiento de esta forma?: La forma para evaluar nuevos tratamientos está establecida por normas y organismos tanto internacionales como nacionales; se asigna el tratamiento al azar para evitar que tanto el participante como el grupo que analiza los datos y los resultados conozcan quien recibe el tratamiento y quien el placebo; de esta manera puede confirmarse la utilidad del tratamiento sin la posibilidad de que existan otros factores que modifiquen los resultados; el azar se establece mediante programas de computación que indican que sujeto recibirá tratamiento o placebo; esto quiere decir que nadie puede saber de antemano que grupo le corresponderá.*

Nota: ¿Qué es la ***hidroxicloroquina****? La hidroxicloroquina es un medicamente ampliamente estudiado y usado para otras enfermedades como la malaria y la artritis reumatoide; se cree que la hidroxicloroquina podría tener efectos sobre el virus que produce la enfermedad COVID-19 o coronavirus, pero antes de poder utilizar este tratamiento en todos los sujetos con esta enfermedad debe confirmarse su eficacia y seguridad en un estudio como el que está siendo invitado a participar.*

Nota: ¿Qué es un ***placebo****? Un placebo es un medicamento similar a la hidroxicloroquina en forma, sabor y olor, pero que no tiene ningún efecto ni beneficioso ni nocivo; puede estar hecho de sustancias como azúcar o almidón; un nuevo tratamiento siempre debe compararse con placebo para saber si su efecto se puede atribuir al medicamento o a otros factores.*

CONSENTIMIENTO INFORMADO PARA PARTICIPAR EN UN ESTUDIO DE INVESTIGACIÓN MÉDICA

Título del estudio: "*Hidroxicloroquina para el tratamiento de infección respiratoria grave por COVID-19: ensayo clínico controlado".*

"Se tomarán datos de su expediente clínico relacionados con su tratamiento y seguimiento además de datos personales relacionados con su estado de salud actual y previo, estos serán confidenciales.".

"Se tomarán muestras semanales para detectar COVID-19 o coronavirus en muestras obtenidas de ***hisopado nasofaríngeo*** *o de secreciones respiratorias durante su hospitalización".*

Nota: ¿Qué es el ***hisopado nasofaríngeo****? Es una técnica para tomar muestras de vía aérea que consiste en introducir un hisopo por la nariz hasta la rinofaringe; esta se enviará a un laboratorio para determinar la presencia de COVID-19.*

"Se tomarán muestras de sangre por punción venosa, llamadas biometría hemática y química sanguínea durante su hospitalización, cada día por 10 días y posteriormente y hasta su alta hospitalaria de forma semanal; estas pruebas se realizarán para buscar si presenta efectos adversos a la medicación y evalúan la función de sus riñones y de su hígado".

"Lo único que se modificará en su tratamiento es el medicamento llamado hidroxicloroquina o placebo comentado anteriormente, el resto del tratamiento siempre será decidido por su equipo médico y nosotros no tendremos influencia en él"

"El estudio concluirá el momento de su alta hospitalaria; no asistirá a ninguna consulta adicional por parte de este estudio".

"Sus responsabilidades como participante se limitan a concluir con el tratamiento (a menos que desee retirarse del estudio) y compartir datos relacionados con sus antecedentes de salud sin faltar a la verdad"

Riesgos asociados con el estudio: Este estudio implica un **riesgo mayor al mínimo** para el participante; esto quiere decir que los riesgos esperados son superiores a los relacionados con el tratamiento que tendría si decidiera no participar; los riesgos potenciales son: posibles reacciones adversas a la hidroxicloroquina, posibles lesiones asociadas con la toma de muestra nasofaríngea y posibles lesiones asociadas a la toma de muestras venosas, otro riesgo potencial es la pérdida de confidencialidad.

CONSENTIMIENTO INFORMADO PARA PARTICIPAR EN UN ESTUDIO DE INVESTIGACIÓN MÉDICA

Título del estudio: "*Hidroxicloroquina para el tratamiento de infección respiratoria grave por COVID-19: ensayo clínico controlado".*

*"La **hidroxicloroquina** es un medicamento conocido y relativamente seguro, algunas personas pueden experimentar **molestias abdominales** manifestadas por **nauseas**, **vómitos** y/o **diarrea**; estos efectos son leves y se resuelven de forma espontánea al terminar el tratamiento o con medidas sintomáticas".*

*"En algunos casos se puede encontrar perdida parcial de la visión, es decir, usted puede empezar a ver borroso, a esto se le llama **retinopatía progresiva** (1 al 10% tras cinco años de consumo de hidroxicloroquina y con dosis mucho mayores a las administradas en este estudio) se limita la frecuencia de esta complicación al usar una dosis de 400mg al día por sólo 10 días; se realizará una evaluación por oftalmología sólo en caso de presentar molestias visuales ya que se aconseja monitorizar esta complicación a partir de un año de tratamiento; existen grupos con un riesgo mayor para esta complicación como ser sujetos con enfermedad crónica del hígado y enfermedad crónica del riñón; por lo que si usted presenta alguna de esas enfermedades no será invitado a participar".*

*"Los potenciales riesgos de la toma de un **hisopado nasofaríngeo** son molestias por el procedimiento manifestadas por tos y lagrimeo y en casos raros sangrado nasal que se limita realizando presión sobre la nariz"*

*"Los potenciales riesgos de la **punción venosa** son dolor y hematoma en el área de punción, esta complicación se limita realizando presión sobre la vena tras la punción y se resuelve espontáneamente"*

"Se toman medidas para evitar que su información personal esté protegida y no pueda ser usada por personas no autorizadas; todos los archivos son almacenados bajo llave y los documentos electrónicos protegidos mediante contraseña; sólo las personas involucradas en la planificación, supervisión, monitoreo o auditorias del estudio tendrán acceso a la información".

"Toda publicación de este ensayo utilizará información sin identificar a los participantes; las organizaciones que podrían inspeccionar y/o copiar la información para evaluación de la calidad y el análisis de los datos se limitan a grupos como el Comité de Ética en Investigación Institucional y otras autoridades regulatorias pertinentes".

CONSENTIMIENTO INFORMADO PARA PARTICIPAR EN UN ESTUDIO DE INVESTIGACIÓN MÉDICA

Título del estudio: "*Hidroxicloroquina para el tratamiento de infección respiratoria grave por COVID-19: ensayo clínico controlado".*

Aclaraciones:

- Su decisión de aceptar o no en el estudio es completamente voluntaria.
- No habrá ninguna consecuencia desfavorable en caso de no aceptar la invitación o en el caso de que desee retirarse del estudio una vez iniciado este.
- Si decide autorizar su participación en el estudio; usted puede solicitar retirarse del mismo en el momento que lo desee pudiendo informar o no las razones de su decisión, las cuales será respetada en su integridad.
- No tendrá un costo extra a la atención habitual
- No recibirá pago por su participación.
- En el transcurso del estudio usted podrá solicitar información actualizada sobre el mismo al investigador responsable y podrá retirarse del estudio si lo desea.
- La información obtenida en este estudio, utilizada para la identificación de cada paciente, será mantenida con estricta confidencialidad por el grupo de investigadores.
- El estudio incluye un sistema de monitoreo interno con reglas para suspensión o eliminación de sujetos que estén participando; si usted u otro participante presenta algún tipo de reacción adversa grave durante el estudio se procederá a informarle, terminar con su participación con el estudio e iniciar el seguimiento y tratamiento de la complicación en caso de ser necesario.

Si considera que no hay dudas ni preguntas acerca de su participación, puede, si así lo desea, firmar la Carta de Consentimiento Informado que forma parte de este documento

Para dudas sobre sus derechos o el protocolo de estudio en general, comentarios, problemas relacionados con el estudio, reclamos o aclaraciones favor de contactarse con:

Contacto Investigador Principal: Dra. Carmen Hernández 54871700 ext 5213

Contacto Presidenta Comité de Ética: Lic. Adriana Espinosa Jove 54871700 ext 5254

CARTA DE CONSENTIMIENTO INFORMADO

Título del estudio: "*Hidroxicloroquina para el tratamiento de infección respiratoria grave por COVID-19: ensayo clínico controlado".*

Yo, ____________________________________ he leído y comprendido la información anterior y mis preguntas han sido respondidas de manera satisfactoria. He sido informado y entiendo que los datos obtenidos en el estudio pueden ser publicados o difundidos con fines científicos. Convengo en autorizar mi participación en este estudio de investigación. Recibiré una copia firmada y fechada de esta forma de consentimiento.

_____________________________________ _____________________

(Participante o nombre y firma de representante legal)

Firma Fecha

_____________________________________ _____________________

Testigo 1: Nombre y firma Dirección

Relación con el participante ______________ Fecha______________

_____________________________________ _____________________

Testigo 2: Nombre y firma Dirección

Relación con el participante ______________ Fecha______________

Esta parte debe ser completada por el Investigador (o su representante):

He explicado al Sr(a). ___________________ la naturaleza y los propósitos de la investigación; le he explicado acerca de los riesgos y beneficios que implica la participación en el estudio. He contestado a las preguntas en la medida de lo posible y he preguntado si tiene alguna duda. Acepto que he leído y conozco la normatividad correspondiente para realizar investigación con seres humanos y me apego a ella.

Una vez concluida la sesión de preguntas y respuestas, se procedió a firmar el presente documento.

_____________________________________ _____________________

Nombre y firma del investigador

PROTOCOLO TRANSCRITO DE LA FORMA QUE SOLICITA EL INER PARA APROBARLO

HIDROXICLOROQUINA PARA EL TRATAMIENTO DE INFECCIÓN RESPIRATORIA GRAVE POR COVID-19: ENSAYO CLÍNICO CONTROLADO.

INVESTIGADOR RESPONSABLE Y ADSCRIPCIÓN

Debe contener: nombre, título y adscripción del investigador principal

Dra. Carmen Margarita Hernández Cárdenas
Jefe Áreas Críticas

INVESTIGADORES PARTICIPANTES Y ADSCRIPCIONES

Debe contener: nombres, títulos y adscripciones de los investigadores participantes.

Dr. Cristóbal Guadarrama Adscrito Unidad de Urgencias Respiratorias
Dr. Luis Felipe Jurado Camacho Adscrito Unidad de Cuidados Intensivos Respiratorios
Dr. José Rogelio Pérez Padilla Departamento en Investigación de Tabaquismo y EPOC
Dra. Ireri Isadora Thirión Romero Departamento en Investigación de Tabaquismo y EPOC
Dr. Joel Armando Vasquez Pérez Departamento en Investigación de Tabaquismo y EPOC

FECHA DE INICIO 10 – abril 2020
FECHA DE TÉRMINO 10 abril 2021

	1	2	3	4	5	6	7	8	9	10	11	21	13	14	15	16	17
Ejecución	x	x	x	x	x	x	x										
Análisis								x	x	X							
Preparación de la publicación										x	x	x					

ULTIMAS PUBLICACIONES

Acute Respiratory Distress Syndrome Secondary to Influenza A(H1N1)pdm09: Clinical Characteristics and Mortality Predictors. Hernández-Cárdenas CM, Serna-Secundino H, García-Olazarán JG, Aguilar-Pérez CL, Rocha-Machado J, Campos-Calderón LF, Lugo-Goytia G. Rev Invest Clin. 2016 Sep-Oct;68(5):235-244. PMID: 27941959 Classifying Acute Respiratory Distress Syndrome Severity: Correcting the Arterial Oxygen Partial Pressure to Fractional Inspired Oxygen at Altitude. Pérez-Padilla R, Hernández-Cárdenas CM, Lugo-Goytia G. Rev Invest Clin. 2016 Jul-Aug;68(4):169-70. PMID: 27623033 Clinical Characteristics and Mortality of Influenza A H1N1 and Influenza-Like Illness in Mexico City in the 2013-2014 Winter Season. Martínez-Briseño D, Torre-Bouscoulet L, Herrera-Zamora Jde J, Díaz-Rico J, Sandoval-Macías G, Pérez-Padilla R, Hernández-Cárdenas C, Regalado-Pineda J, Salas-Hernández J, Santillán-Doherty P. Rev Invest Clin. 2016 May-Jun;68(3):147-53. PMID: 27409002 Influenza A (H1N1pdm09)-Related Critical Illness and Mortality in Mexico and Canada, 2014. Dominguez-Cherit G, De la Torre A, Rishu A, Pinto R, Ñamendys-Silva SA, Camacho-Ortiz A, Silva-Medina MA, Hernández-Cárdenas C, Martínez-Franco M, Quesada-Sánchez A, López-Gallegos GC, Mosqueda-Gómez JL, Rivera-Martinez NE, Campos-Calderón F, Rivero-Sigarroa E, Hernández-Gilsoul T, Espinosa-Pérez L, Macías AE, Lue-Martínez DM, Buelna-Cano C, Ramírez-García Luna AS, Cruz-Ruiz NG, Poblano-Morales M, Molinar-Ramos F, Hernandez-Torre M, León-Gutiérrez MA, Rosaldo-Abundis O, Baltazar-Torres JÁ, Stelfox HT, Light B, Jouvet P, Reynolds S, Hall R, Shindo N, Daneman N, Fowler RA. Crit Care Med. 2016 Oct;44(10):1861-70. doi: 10.1097/CCM.0000000000001830.

PMID: 2735908 Population Pharmacokinetics of Gentamicin in Mexican Children with severe malnutrition Lares-Asseff I, Pérz-Guillé MG, Camacho Vieyra GA, Pérez AG, Peregrina NB, **Lugo Goytia** G. Pediatr Infect Dis J. 2016 Aug;35(8):872-8. doi: 10.1097/INF.0000000000001204. PMID: 27420805

DEFINICIÓN DEL PROBLEMA

El COVID – 19 es un nuevo tipo de neumonía viral descrito en diciembre 2019 en Wuhan – China; representa un problema de salud público importante por su rápida propagación y la capacidad de generar neumonía grave en pacientes susceptibles; al momento no se a descrito ningún tratamiento específico para el virus siendo su manejo principalmente el soporte.

PREGUNTA DE INVESTIGACIÓN

¿El tratamiento por 10 días con 400 mg/día de hidroxicloroquina comparada con placebo reduce la mortalidad intrahospitalaria en sujetos con enfermedad grave por COVID - 19?

ANTECEDENTES

Se deberá proveer información de base para poder entender la relevancia del presente estudio y el propósito y fundamento del estudio.

Se deberá incluir un resumen de los datos de estudios no clínicos que tengan potencialmente una importancia clínica y de estudios clínicos y epidemiológicos relevantes para el presente estudio, así como las referencias de la literatura relevantes al estudio y que provean un sustento para el estudio.

El brote de infección respiratoria por el coronavirus 2019 (2019-nCOV) inició en diciembre del 2019, en la ciudad de Wuhan, provincia de Hubei (China) [1-5]. De esta ciudad, el brote se ha ido extendiendo a varias provincias de China y a otros países [6]. Hasta el 16 de Febrero del 2020, se habían reconocido 28 países con casos confirmados, con 70,548 infecciones y 1770 muertes en China, y 413 infecciones en Japón [6]. Como en otros coronavirus, el del SARS, y el del MERS, la infección original humana probablemente se obtuvo de contacto con animales, pero se ha demostrado una eficaz transmisión entre humanos, con una tasa de crecimiento mayor a 1, estimada de 2.6 [7], y una duplicación rápida de casos que aseguran no solo el mantenimiento de la epidemia si no su expansión.

Hasta el momento actual no se cuenta con un tratamiento efectivo disponible para el COVID-19, sin embargo in vitro, se reporto recientemente la efectividad in vitro del Remdesivir, y de la cloroquina, esta ultima en concentraciones capaces de ser obtenidas in vivo en pacientes que la usan [8]. La cloroquina e hidroxicloroquina, son medicamentos originalmente antimaláricos, en uso regular por décadas y con una toxicidad limitada, a un costo muy bajo, y en caso de ser efectivo in vivo, podría ser un medicamento de gran utilidad. El efecto antiviral amplio, se ha atribuido al incremento del pH de los endosomas, que se requieren para la fusión del virus con la célula y también por interferencia con la glicosilación de los receptores celulares del SARS-COV[9-11]

Adicionalmente al efecto antiviral, se le han atribuido efectos inmunomoduladores que podrían potenciar el efecto antiviral y el impacto sistémico de las infecciones graves.
La gama de presentaciones clínicas del 2019-CoV, es amplia, desde infecciones asintomáticas hasta infecciones fatales, con daño pulmonar grave, falla respiratoria, y falla multiorgánica. El daño grave se ha asociado, como en la influenza grave, a una reacción inflamatoria sistémica o una tormenta de citocinas, que podría ser atenuada por el efecto inmunomodulador de la cloroquina o hidroxicloroquina.

La tasa de letalidad general de la enfermedad por COVID-19 se ha estimado alrededor del 2%, pero en los casos graves en falla respiratoria, o requiriendo cuidados críticos, es considerablemente mayor, entre 10-15%, pero fácilmente podría incrementarse hasta el 20-30% dependiendo de los criterios de entrada a una unidad de terapia intensiva.
Recientemente se publicó una carta [12] que describe que en Febrero 17, el Consejo Estatal de China dio un aviso de prensa indicando que la Cloroquina habia demostrado marcada eficacia y seguridad aceptable para el tratamiento de la neumonía por COVID-19 en ensayos multicéntricos de China con más de 100 pacientes de 10 hospitales.

JUSTIFICACIÓN

Se carece de un tratamiento farmacológico aprobado para el COVID - 19, y basados en la efectividad in Vitro de la cloroquina, su seguridad demostrada por décadas, su costo bajo, se propone un ECC, doble ciego, para evaluar la eficacia y seguridad del compuesto derivado hidroxicloroquina en sujetos hospitalizados con enfermedad respiratoria grave por COVID - 19.

HIPÓTESIS

El tratamiento con Hidroxicloroquina 400 mg/día por 10 días reduce la mortalidad intrahospitalaria comparada con placebo en sujetos con enfermedad respiratoria grave por COVID-19.

OBJETIVO (S) GENERAL (ES)

Estimar si el tratamiento por 10 días con 400 mg/día de hidroxicloroquina reduce la mortalidad intrahospitalaria comparada con placebo en sujetos con enfermedad grave por COVID – 19.

OBJETIVOS ESPECÍFICOS

A) Días de hospitalización
B) Necesidad de apoyo ventilatorio mecánico
C) Días de soporte ventilatorio mecánico.

Incidencia de efectos adversos grado 3 y 4.

DISEÑO EXPERIMENTAL

1. INVESTIGACIÓN CLÍNICA x

FAVOR DE INDICAR EL TIPO DE ESTUDIO

x EXPERIMENTAL

x LONGITUDINAL

x PROSPECTIVO

"Indicar lo que se pretende obtener para el conocimiento en el tema de estudio al desarrollar esta investigación"

1. Tratamiento x

Indicar la línea de investigación de la que se deriva este proyecto:

1. Programa de Estudio sobre la Influenza y otros Virus Respiratorios. x

MATERIAL Y MÉTODOS

UTILIZAR LAS HOJAS NECESARIAS

B) Lugar del estudio

C) Descripción de la población de estudio

D) Procedimientos del estudio

E) Número necesario de sujetos de investigación

F) Criterios de inclusión y exclusión

G) Captura, procesamiento, análisis e interpretación de la información.

H) Áreas críticas y Hospitalización del Instituto Nacional de Enfermedades Respiratorias Ismael Cosío Villegas

I) Mayores de 18 años con diagnóstico confirmado de COVID – 19 por RT-PCR por muestra faríngea, naso-faríngea o en aspirado traqueal/lavado bronquioalveolar, con diagnóstico de enfermedad grave por COVID - 19

J) Ensayo clínico controlado aleatorizado, doble ciego, fase III.
   a. Se aleatorizará de forma estratificada a sujetos que ingresen al INER con diagnóstico de enfermedad grave por COVID – 19. La aleatorización será central realizada en software dedicado. Los médicos tratantes, enfermería y resto del equipo tratante y los evaluadores de los desenlaces serán cegados al grupo que corresponden cada uno de los sujetos.
   b. Se estratificarán a los sujetos incluidos en dos grupos para la aleatorización:
      i. Grupo A: Sujetos con diagnostico de enfermedad grave por COVID 19 que requieran hospitalización.
      ii. Grupo B: Sujetos con diagnostico de enfermedad grave por COVID 19 que requieran manejo ventilatorio invasivo y/o manejo en la unidad de cuidados intensivos respiratorios al momento de la aleatorización.
   c. **Desenlace principal:**

En sujetos del grupo A: Proporción de pacientes que requieren ventilación mecánica invasiva y/o tasa de muerte a 30 días posterior a la aleatorización

En sujetos del grupo B: Duración en días de ventilación mecánica invasiva o tasa de muerte a 30 días posterior a la aleatorización

d. **Desenlaces secundarios:**
   i. Días de hospitalización
   ii. Necesidad de apoyo ventilatorio mecánico
   iii. Días de soporte ventilatorio mecánico.
   iv. Incidencia de efectos adversos grado 3 y 4.

e. **<u>Pruebas realizadas:</u>**
   i. Panel de 10 virus respiratorios y de COVID-19 basal, laboratorios como biometría hemática, química sanguínea y pruebas de funcionamiento hepático que corresponden a una toma de muestra venosa de 5 mL el equivalente a una cucharada de postre.
   ii. **<u>Cada semana:</u>** prueba viral RT-PCR en aspirado faríngeo, nasofaríngeo, o en muestra bronquial o de lavado bronquial o aspirado traqueal en intubados. Y semanalmente laboratorios como biometría hemática, química sanguínea y pruebas de funcionamiento hepático -que corresponden a una toma de muestra venosa de 5 mL el equivalente a una cucharada de postre- para seguimiento de reacciones adversas. Dentro de esta toma sanguínea en el tubo de química sanguínea se analizará kit de citosinas al primer y séptimo día.

f. **<u>Intervenciones específicas del protocolo:</u>**
   i. Los sujetos que ingresen al grupo experimental recibirán hidroxicloroquina por vía oral o por sonda nasogástrica, 400 mg al día por 10 días. El grupo placebo recibirá una tableta idéntica por 10 días. Se propone un periodo de tratamiento de 10 días principalmente por que no se conoce un periodo óptimo; los grupos de trabajo sugieren un uso entre 5 a 14 días; la caja de hidroxicloroquina contiene 20 tabletas de 200mg; justificamos el periodo de 10 días por encontrarse en el punto intermedio entre las varias recomendaciones y por ser más práctico utilizar una caja por sujeto

<u>Seguimiento:</u> El seguimiento durará hasta el alta hospitalaria.

Los efectos adversos serán reportados al comité de ética, COFEPRIS y al productor del fármaco (Sanofi México, S.A. de C.V.)

ANALISIS ESTADÍSTICO

Se realizará el análisis en software estadístico R-Rstudio. Se utilizará estadística descriptiva de acuerdo al tipo de variable y distribución. Se comparará la mortalidad entre el grupo placebo e intervención mediante prueba exacta de fisher. Se compararán tiempos a los desenlaces y supervivencia global y por grupo ajustando por variables confusoras (edad, tiempo del inicio de síntomas al inicio de tratamiento, comorbilidades) mediante modelo de riesgos proporcionales de cox.

Análisis:

- La tasa de mortalidad (30 días), la proporción de pacientes que necesitan ventilación mecánica y los días de ventilación mecánica, se compararán en un modelo multivariado, que incluye un indicador de estratos de aleatorización, ajustando las variables de confusión (edad, tiempo desde el inicio de los síntomas hasta el inicio de tratamiento, comorbilidades), se realizará una comparación similar para los resultados secundarios: duración de la hospitalización, duración de la oxigenoterapia.
- También compararemos el tiempo hasta la muerte ajustando las variables de confusión (edad, tiempo desde el inicio de los síntomas hasta el inicio del tratamiento, comorbilidades) utilizando el modelo de riesgos proporcionales de Cox.
- Habrá un CMDS, un grupo asesor multidisciplinario, compuesto por un experto en estadística, un investigador clínico, un experto en ética y un clínico relacionado con la influenza y el coronavirus, ninguno involucrado en el ensayo clínico y, por lo tanto, independiente, que en el medio de los ensayos tendrá acceso a los datos y al código de aleatorización para evaluar los datos, incluida la seguridad y los resultados.

  Investigador clínico: Gustavo Lugo, farmacólogo clínico, anestesiólogo e intensivista que actuará como coordinador. Dra. Angélica Portillo, especialista en oído, nariz y garganta y con una maestría en ciencias médicas.

  Estadísticas: Dra. Rosario Fernández, Jefa del Departamento de Epidemiología del INER.

  Ética: Dr. Patricio Santillán, director médico de INER.

  Clínico: Dr. Arturo Martínez, Infectólogo, experto en infecciones similares a la gripe y ahora en infección por COVID-19.

K) TAMAÑO DE MUESTRA

La mortalidad reportada en el COVID-19 grave se encuentra entre el 8% a incluso al 60% dependiendo la fuente[1,2] y la OMS sugiere al menos 400 sujetos para la estimación de la muestra en ensayos clínicos que evalúen terapias para el COVD-19 (3). Al momento se desconocen muchas características del comportamiento y desenlaces del COVID-19 especialmente en nuestro medio; por eso utilizamos la estimación de una mortalidad alrededor del 15% en COVID-19 grave con una reducción del 50% de la mortalidad con el tratamiento obteniendo en el cálculo de muestra un número de sujetos similar al recomendado por la OMS.

Nivel de significancia de 2 colas 95, alfa 5%
Potencia (1-beta, posibilidad de detección): 80%
Tratados vs controles 1:1
Mortalidad estimada en controles 15%
Mortalidad estimada en experimentales 7.5%, reducción de mortalidad de 7.5%, que es el 50% (RR 0.5).

<u>Estimaciones (Open-Epi)</u>

	Kelsey	Fleiss	Fleiss with CC	
Muestra experimental	280	279	305	
Muestra control		280	279	305

Muestra total : 560 558 610

Se decide incluir 600 sujetos para el ensayo clínico, es decir 300 sujetos en el grupo de tratamiento experimental y 300 sujetos en el grupo control.

E) **Criterios de inclusión**

L) Firma de Consentimiento informado por el sujeto (o representante legal) entendiendo y aceptando los procedimientos y medicación del estudio descritos a continuación:
   a. Aleatorización a hidroxocloroquina o placebo idéntico.
   b. Recolección de muestra orofaringea (o aspirado traqueal o lavado bronquioalveolar) y muestras sanguíneas para confirmación y seguimiento de COVID – 19.
   c. Hospitalización o ingreso a unidad de cuidados intensivos.

M) En caso de ser mujer prueba de embarazo negativa.

N) Inicio de síntomas menor a 14 días.

O) Al menos 18 años cumplidos al momento de la aleatorización.

P) Confirmación por laboratorio de infección por SARS-CoV-2 determinada por RT-PCR de muestra faríngea, nasofaríngea, lavado broncoalveolar o aspirado traqueal antes de la aleatorización y/o infección pulmonar documentada en placa de tórax o tomografía definida por lesiones difusas predominantemente periféricas y subpleurales del tipo consolidación y vidrio deslustrado.

Q) Enfermedad grave por COVID – 19 definida por al menos uno de los siguientes:
   a. SpO2 menor o igual a 90 % a una altura de 2240 m, en el valle de México o Puebla o disminución de más del 3% de saturación basal
   b. Requiere ventilación mecánica y/o oxígeno suplementario (o incremento del apoyo de oxigeno suplementario en hipoxemia crónica)
   c. Cumple criterios de sepsis/choque séptico.

Criterios de exclusión:

R) Mayor de 80 años

S) Hipersensibilidad conocida a hidroxicloroquina manifestada por anafilaxia.

T) Consumo previo a la hospitalización de hidroxicloroquina o cloroquina. Tratamiento actual o del mes pasado.

U) Decisión del médico responsable de excluir al participante del estudio.

V) Antecedente de enfermedad hepática crónica. (Child-Pugh B o C).

W) Antecedente de enfermedad renal crónica. (TFG menor o igual a 30).

X) Prueba de embarazo positiva.

Y) Contraindicación para iniciar o continuar con hidroxicloroquina (incluidos, entre otros, antecedentes de anafilaxia, un QTc> 450 / 500mmseg por ECG, interacción farmacológica peligrosa o patología retiniana).

Z) Infección previa por COVID-19

AA) Traslado del paciente a otra unidad hospitalaria.

. **Criterios de retiro/eliminación:**

BB) Decisión del médico responsable de retirar al participante del estudio.

CC) Transferencia del paciente a otra unidad hospitalaria.

DD) Hipersensibilidad nueva a hidroxicloroquina manifestada por anafilaxia.

EE) Efecto adverso grado 3 o 4

FF) Decisión del participante o representante de retirarse del estudio.

Centros que pueden participar además del INER (Centros COVID)

- Instituto Nacional de Ciencias Médicas y Nutrición "Salvador Zubirán"
- Hospital Juárez de México
- Hospital General de México "Dr. Eduardo Liceaga"
- Hospital Regional de Alta Especialidad de Iztapaluca
- Hospital Regional de Alta Especialidad de Mérida
- Hospital Regional de Alta Especialidad de Oaxaca

CONSIDERACIONES ÉTICAS

a) Indique el tipo de riesgo de la investigación.

El tipo de riesgo deberá ser considerado de a cuerdo a lo establecido en el Art. 17 del REGLAMENTO de la Ley General de Salud en Materia de Investigación para la Salud:

- Investigación sin riesgo.
- Investigación con riesgo mínimo.
- Investigación con riesgo mayor que el mínimo.

b) Para los protocolos de investigación en animales, hacer mención de la reglamentación vigente:

- Reglamento de la Ley General de Salud en Materia de Investigación para la Salud, Título Séptimo, de la Investigación que incluya la utilización de animales de experimentación.
- Ley Federal de Sanidad Animal.
- Reglamento de la Ley Federal de Sanidad Animal.
- NOM-062-ZOO-1999, Especificaciones técnicas para la producción, cuidado y uso de animales de laboratorio.
- Guía para el cuidado y uso de los animales de laboratorio "Guide for the Care and use of Laboratory Animals eigth Edition, Institute for Laboratory Animal Research Council".
- Lineamientos para el sacrificio humanitario de los animales "AVMA Guidelines for the Euthanasia of Animals: 2013 Edition".

Este estudio implica un **riesgo mayor al mínimo** para el participante; esto quiere decir que los riesgos esperados son superiores a los relacionados con el tratamiento que tendría si decidiera no participar; los riesgos potenciales son: posibles reacciones adversas a la hidroxicloroquina, posibles lesiones asociadas con la toma de muestra nasofaríngea y posibles lesiones asociadas a la toma de muestras venosas.

REFERENCIAS BIBLIOGRÁFICAS

UTILIZAR LAS HOJAS NECESARIAS

1. Chan JF, Yuan S, Kok KH, To KK, Chu H, Yang J, Xing F, Liu J, Yip CC, Poon RW, Tsoi HW, Lo SK, Chan KH, Poon VK, Chan WM, Ip JD, Cai JP, Cheng VC, Chen H, Hui CK, Yuen KY. A

familial cluster of pneumonia associated with the 2019 novel coronavirus indicating person-to-person transmission: a study of a family cluster. *Lancet* 2020.

2. Chen N, Zhou M, Dong X, Qu J, Gong F, Han Y, Qiu Y, Wang J, Liu Y, Wei Y, Xia J, Yu T, Zhang X, Zhang L. Epidemiological and clinical characteristics of 99 cases of 2019 novel coronavirus pneumonia in Wuhan, China: a descriptive study. *Lancet* 2020.

3. Holshue ML, DeBolt C, Lindquist S, Lofy KH, Wiesman J, Bruce H, Spitters C, Ericson K, Wilkerson S, Tural A, Diaz G, Cohn A, Fox L, Patel A, Gerber SI, Kim L, Tong S, Lu X, Lindstrom S, Pallansch MA, Weldon WC, Biggs HM, Uyeki TM, Pillai SK, Washington State -nCo VCIT. First Case of 2019 Novel Coronavirus in the United States. *The New England journal of medicine* 2020.

4. Zhou P, Yang XL, Wang XG, Hu B, Zhang L, Zhang W, Si HR, Zhu Y, Li B, Huang CL, Chen HD, Chen J, Luo Y, Guo H, Jiang RD, Liu MQ, Chen Y, Shen XR, Wang X, Zheng XS, Zhao K, Chen QJ, Deng F, Liu LL, Yan B, Zhan FX, Wang YY, Xiao GF, Shi ZL. A pneumonia outbreak associated with a new coronavirus of probable bat origin. *Nature* 2020.

5. Zhu N, Zhang D, Wang W, Li X, Yang B, Song J, Zhao X, Huang B, Shi W, Lu R, Niu P, Zhan F, Ma X, Wang D, Xu W, Wu G, Gao GF, Tan W, China Novel Coronavirus I, Research T. A Novel Coronavirus from Patients with Pneumonia in China, 2019. *The New England journal of medicine* 2020.

6. Centers of Disease Control. 2019 Novel Coronavirus. 2020 [cited 2020 February 7]; Available from: https://http://www.cdc.gov/coronavirus/2019-ncov/locations-confirmed-cases.html

7. Zhao S, Musa SS, Lin Q, Ran J, Yang G, Wang W, Lou Y, Yang L, Gao D, He D, Wang MH. Estimating the Unreported Number of Novel Coronavirus (2019-nCoV) Cases in China in the First Half of January 2020: A Data-Driven Modelling Analysis of the Early Outbreak. *Journal of clinical medicine* 2020: 9(2).

8. Wang M, Cao R, Zhang L, Yang X, Liu J, Xu M, Shi Z, Hu Z, Zhong W, Xiao G. Remdesivir and chloroquine effectively inhibit the recently emerged novel coronavirus (2019-nCoV) in vitro. *Cell research* 2020.

9. Savarino A. Use of chloroquine in viral diseases. *The Lancet Infectious diseases* 2011: 11(9): 653-654.

10. Savarino A, Boelaert JR, Cassone A, Majori G, Cauda R. Effects of chloroquine on viral infections: an old drug against today's diseases? *The Lancet Infectious diseases* 2003: 3(11): 722-727.

11. Yan Y, Zou Z, Sun Y, Li X, Xu KF, Wei Y, Jin N, Jiang C. Anti-malaria drug chloroquine is highly effective in treating avian influenza A H5N1 virus infection in an animal model. *Cell research* 2013: 23(2): 300-302.

12. Gao J, Tian Z, Yang X. Breakthrough: Chloroquine phosphate has shown apparent efficacy in treatment of COVID-19 associated pneumonia in clinical studies. *Bioscience trends* 2020.

DESGLOSE DE RECURSOS HUMANOS Y TÉCNICOS REQUERIDOS PARA EL ESTUDIO

Especificar aquellos que requieran ser autorizados por la Subdirección de Servicios Auxiliares de Diagnóstico y Paramédicos

RECURSO	NÚMERO REQUERIDO	ORIGEN O SITIO DONDE SE REALIZA	FUENTE DEL FINANCIAMIENTO
Investigador principal	1	INER	INER
Sub investigadores	8	INER	INER
Coordinador de estudio	1	INER	CONACYT
Ejecutadores	3	INER	CONACYT
Oxígeno	500	INER	INER
Hospitalizacion urgencias	500	INER	INER
Hospitalizacion pabellón	500	INER	INER
Hospitalizacion UCIR	500	INER	INER
Kits de laboratorio	500	INER	INER
Ventilación mecánica	500	INER	INER
Medicamentos	500	INER	INER
Los hospitales participantes tendran una distribución similar			
