# Supplementary material for: Hydroxychloroquine for the treatment of severe respiratory infection by COVID-19: A randomized controlled trial
Source: PLoS One. 2021 Sep 28;16(9):e0257238. doi: 10.1371/journal.pone.0257238 (PMC8478184; doi:10.1371/journal.pone.0257238)
Supplement: S4 File — (PDF) [file pone.0257238.s008.pdf]

1. PROJECT PRESENTATION
   1.1 Applicant information: Carmen Margarita Hernández Cárdenas MD. Critical care Chief.
   1.2 Descriptive title of the project: **HYDROXYCHLOROQUINE FOR THE TREATMENT OF SEVERE RESPIRATORY INFECTION BY COVID-19: MULTI-CENTRIC CONTROLLED CLINICAL TRIAL**

2. PROJECT SUMMARY

COVID - 19 is a new type of viral pneumonia described in December 2019 in Wuhan - China; it represents a major public health problem due to its rapid spread and the ability to generate severe pneumonia in susceptible patients; at the moment, no specific treatment for the virus has been described, its management being mainly a supportive one. Our objective is to estimate whether treatment with hydroxychloroquine 400 mg / day for 10 days compared to placebo reduces in-hospital mortality in subjects with severe respiratory disease due to COVID-19. Secondary outcomes will be days of mechanical ventilation, requirement of mechanical ventilatory support, days of hospitalization and the cumulative incidence of serious adverse events.

Design: Double-blind randomized controlled trial to evaluate the safety and efficacy of hydroxychloroquine for the treatment of severe disease by COVID-19.

Subjects elegible Adults older than 18 years with a diagnosis of severe disease (requiring hospitalization or intensive care) of COVID-19 (confirmed by RT-PCR). Subjects will be stratified into two groups (subjects not requiring or requiring ventilatory support at the time of randomization). The analysis will be carried out in R-Rstudio statistical software, considering an interim analysis when half of the sample size is recruited (150 per group).

3. DESCRIPTION OF THE PROJECT

3.1 INTRODUCTION AND BACKGROUND

The outbreak of respiratory infection by the coronavirus 2019 (SARSCOV-2) started in December 2019, in Wuhan City, Hubei Province (China) [1-5]. From this city, the outbreak has been spreading to various provinces of China and other countries [6]. As of February 16, 2020, 28 countries had been recognized with confirmed cases, with 70,548 infections and 1,770 deaths in China, and 413 infections in Japan. [6]. By March 24, > 190 Countries have been affected.

As in other coronaviruses, SARS and MERS, the original human infection was probably obtained from contact with animals, but effective human-to-human transmission has been demonstrated, with a growth rate greater than 1, estimated in 2.6[7], and a rapid duplication of cases that ensure not only the maintenance of the epidemic but also its expansion.

Until now, there is no effective treatment available for COVID-19, however the in-vitro effectiveness of Remdesivir and chloroquine was recently reported, the latter in concentrations capable of being obtained in in-vivo patients who use it [8]. Chloroquine and hydroxychloroquine are originally antimalarial drugs. The have had regular use for decades with a well documented benefit/risk profile, at a very low cost, and if they are proven effective in vivo, they could be very useful drugs. The broad antiviral effect has been attributed to increased pH of endosomes, which are required for virus-cell fusion and also by interference with glycosylation of SARS-VOC cell receptors.[9-11] In addition to the antiviral effect, immunomodulatory effects have been found that could enhance the antiviral effect and the systemic impact of serious infections.

The range of 2019-CoV clinical presentations is wide, from asymptomatic infections to fatal infections, with severe lung damage, respiratory failure, and multi-organ failure. As in severe influenza, severe damage has been associated with a systemic inflammatory reaction or cytokine storm, which could be attenuated by the immunomodulatory and antinflammatory effect of chloroquine or hydroxychloroquine.

The overall fatality rate of COVID-19 disease has been estimated at around 2.3%, but in severe cases of respiratory failure, requiring hospitalization or critical care, it is considerably higher, between 10-15%, but could easily increase to 20-30% in those requiring intensive care and mechanical ventilation.

A letter was recently published [12] Describing that on February 17, the Chinese State Council issued a press release stating that Chloroquine had demonstrated marked efficacy and acceptable safety for the treatment of COVID-19 pneumonia in multicentre trials in China with more than 100 patients out of 10 hospitals, but no report in a peer revewed journal has appeared.

3.2 JUSTIFICATION

There is no approved pharmacological treatment for COVID-19, and based on the in-vitro effectiveness of chloroquine, its proven safety for decades, its low cost, a double-blind RCT is proposed to evaluate the efficacy and safety of Hydroxychloroquine derived

compound, generally with better tolerance and safety, in hospitalized patients with severe respiratory disease due to COVID - 19.

3.3 GENERAL OBJECTIVE

Estimate whether 10-day treatment with 400 mg / day of hydroxychloroquine (HCQ), 200 mg BID, reduces the 30 day mortality in hospitalized patients with severe COVID-19 disease.

3.3.1 SPECIFIC OBJECTIVES

A) Assess whether days of hospitalization are decreased with HCQ compared to placebo.
B) Assess whether days of supplemental oxygen therapy are decreased with HCQ compared with placebo.
C) Assess whether the days for viral test negative (one per week) decrease with HCQ compared with placebo
D) Assess if the need of ventilatory support with HCQ is decreased compared with placebo.
E) Assess if the days of mechanical ventilation in patients treated with HCQ decrease compared with placebo.
D) Evaluate the safety of HCQ.

3.4 THEORETICAL AND CONCEPTUAL APPROACH

Phase III, multicenter, randomized, double-blind, controlled clinical trial; The coordinating center will be INER and it is expected to recruit other hospital centers in Mexico City and in other states. Planned to be carried out in critical areas (Emergencies, intensive care, postoperative recovery) and in Hospitalization services of the National Institute of Respiratory Diseases Ismael Cosío Villegas and participating hospitals.

Subjects eligible will be adults over 18 years of age with a confirmed diagnosis of COVID-19 by RT-PCR (in a pharyngeal, nasopharyngeal sample or in a tracheal aspirate / bronchial lavage) that requires hospitalization for severe or critical COVID-19.

1. **Blinding:** Patients who agree to participate, who are hospitalized at INER or collaborating centers, with a diagnosis of severe disease due to COVID-19 will be randomized. The randomization will be centralized, carried out by a dedicated software, and randomization will be utilized to label the experimental drug and the placebo. The treating doctors, the nursing staff and the rest of the treating team and the evaluators of the outcomes will be blinded to the group corresponding to each one of the subjects.

2. **Risk stratification** Subjects included in two groups will be stratified for randomization:
    a. Stratum A: Subjects diagnosed with severe disease by COVID-19 requiring hospitalization that do not require invasive ventilatory management.
    b. Stratum B: Subjects diagnosed with severe disease by COVID-19 requiring invasive ventilatory management and / or management in the intensive care respiratory unit at the time of randomization.
3. **Main outcome:**
    a. Mortality rate at 30 days after randomization.
4. **Secondary outcomes:**
    a. Proportion of patients needing invasive ventilatory support (stratum A only)
    b. Duration in days of invasive mechanical ventilation for patients requiring such procedure
    c. Time in days from randomization to hospital discharge.
    d. Time in days from randomization to end of oxygen therapy
    e. Time in days to negative viral test using weekly RT-PCR testing
    f. Incidence of AEs and AEs leading to treatment discontinuation
    g. • Incidence of serious adverse events

5. **Tests carried out:**
    a. Panel of 10 respiratory viruses and 2019-nCoV
    b. Every week: RT-PCR viral test in pharyngeal, nasopharyngeal aspirate, and in bronchial sample or bronchial lavage or tracheal aspirate in intubated.
    c. Routine tests (Blood cell count, blood chemistry and liver function tests and ECG or cardiac monitoring)
6. **Specific protocol interventions:**
    a. Subjects entering the experimental group will receive hydroxychloroquine orally or by nasogastric tube (refer to the protocol procedures manual for the method of crushing tablets and administration via nasogastric tube), 200 mg every 12 hours, for 10 days. The placebo group will receive an identical placebo for 10 days. 10 days are tentatively proposed although the working groups suggest a use between 5 to 14 days; the box of hydroxychloroquine contains 20 tablets of 200mg; We justify the 10-day period because it is somewhere in between the various recommendations and because it is more practical to use one box per subject
    b. Patients in both strata will be treated according to protocols of participating institution under the responsability of the physician in charge: all interventions, medications for all purposes, or non-drug interventions will be registered.
    c. Patients will be followed up to 30 days after randomization by telephone if discharge occur earlier than Day 30
    d. The Investigator is responsible for detecting, documenting, and recording events that meet the definition of an AE or SAE and remain responsible for following up AEs that are serious, considered related to the study

intervention or study procedures, or that caused the participant to discontinue the study

e. Adverse events will be reported to CE and to manufacturer of the drug (Sanofi México, S.A. de C.V.)

Statistical analysis: The analysis will be carried out in R-Rstudio statistical software. Descriptive statistics will be used according to the type of variable and distribution.
The main outcomes will be compared using parametric and non-parametric tests according to the type of variable and distribution.
Death rate (at 30 days), the primary outcome, will be compared between treatment groups using a logistic regression model, adjusting for age, time from onset of symptoms to randomization, comorbidities (diabetes, hypertension, obesity, tobaco smoking) taking into account the randomization strata.
From the secondary endpoints, proportion of patients needing mechanical ventilation, will be compared also by a similar logistic regression model as that proposed for 30 days-death rate. Days of mechanical ventilation, days of hospitalization and days of oxygen therapy will be compared in a multivariate model, including an indicator of randomization strata, adjusting for confounding variables (age, time from onset of symptoms to start of treatment, and comorbidities, especially diabetes, obesity, tobacco smoking, hypertension)
We also will compare time to death and time to a negative viral test, adjusting for confounding variables (age, time from onset of symptoms to start of treatment, comorbidities) using the Cox proportional hazards model.

There will be a CMDS, a multidisciplinary advisory group, made up of an expert in statistics, a clinical researcher, an expert in ethics and a clinician related to influenza and coronavirus, none involved in the clinical trial and therefore independent, who in the middle of the Trials will have access to the data and the randomization code to evaluate the data including safety and efficacy results. The study results will remain blinded to trial team and sponsor representatives, so that not to impact final interpretation of the study results.

Clinical Researcher: Gustavo Lugo, clinical pharmacologist, anesthesiologist and intensivist who will act as coordinator. Dra. Angélica Portillo, Ear nose and Throat specialist and with a masters degree in medical sciences.

Statistics: Dr. Rosario Fernández, Head of the Department of Epidemiology of INER.

Ethics: Dr. Patricio Santillan, medical director of INER
Clinician: Dr. Arturo Martínez, Infectologist, expert in influenza-like infections and now in COVID-19 infection

The aim of the committee is to assess the advantages and disadvantages of continuing the trial in the analysis in the middle of the sample, especially if it is considered ethical to continue the recruitment, given the results found in the middle of the study.

In principle, there are no controlled studies with which to compare but they may appear in the course of the trial.

An interim analysis is planned, upon completing half of the sample, that can identify if a noticeable difference is found in the main outcome measure, demonstrating an advantage in effectiveness, which, due to its magnitude, leads to stopping the trial.

Also, to indicate significant adverse effects or toxicity.
The final statistical analysis will be carried out taking into account this intermediate analysis. The CMDS will provide a written report (not accesible to anyone outside the CMDS) of its observations. It will be considered sufficient to stop the trial because efficacy in the first interim analysis (1 interim and final), if P <0.0054 and the final analysis will remain significant with p <0.0492 (according to O´Brien-Flemming). It can also be stopped if probability of success at the final analysis, given the interim results, is too low.

1. **Sample size:** Mortality reported in severe COVID-19 ranges depending on the source, but in China among hospitalized patients with pneumonia has been reported from 4-15% (1,2,4). The WHO suggests at least 400 subjects for sample estimation in clinical trials evaluating therapies for COVD-19, although severe outcomes or deaths increase considerably in hospitalized patients or those in intensive care as those elegible for this trial. (3). At the moment, many behavioral characteristics and outcomes of COVID-19 are unknown, especially in our setting; For this reason, we used the estimate of a mortality of around 15% in severe COVID-19 with a 50% reduction in mortality with treatment, obtaining in the sample calculation a number of subjects similar to that recommended by the WHO.
    a. 2-tailed significance level 95, alpha 5%
    b. Potency (1-beta, chance of detection): 80%
    c. Treaties vs. controls 1: 1
    d. Estimated mortality in controls 15%
    e. Estimated mortality in experimental 7.5%, reduction in mortality of 7.5%, which is 50% (RR 0.5).

    <u>Estimations (Open-Epi)</u>

	Kelsey	Fleiss	Fleiss with CC
Experimental tests	**280**	279	305

Control sample	**280**	279	305
Total samples:	**560**	558	610

The sample would be rounded to **300** per group. Total study group = 600 subjects

2. **Entry criteria**
    a. At least 18 years old
    b. Signed informed consent.
    c. Confirmed infection of COVID-19 by RT-PCR and pharyngeal and nasopharyngeal specimen, or in bronchoalveolar lavage or tracheal aspirate.
    d. Documented lung infection due to opacities in the chest plate or in a chest computed tomography without contrast medium
    e. Hypoxemic type respiratory failure, documented by a SaO2 <90% in ambient air, or a PaO2 / FIO2 <250, at an altitude of 2240 m, in the Valley of Mexico or Puebla, or values equivalent to other altitudes above the sea level
    f. <14 days from onset of symptoms
3. **Exclusion criteria:**
    a. Positive pregnancy test.
    b. Contraindication to start or continue hydroxychloroquine (including but not limited to history of pre-existing maculopathy, history of prolonged QTc syndrome or other cardiac condition that, by the judgement of the Investigator, would put the patient at higher risk for QTc prolongation or sudden cardiac death, severe skin reactions, patient with uncorrected hypokalemia or hypomagnesemia, a contraindicated drug interaction according to local drug labelling, , known hypersensitivity to hydroxychloroquine or chloroquine, severe hepato-cellular disorder, severe renal insufficiency
    c. Previous COVID-19 infection
    d. Decision of the responsible doctor not to participate.
    e. Transfer of the patient to another hospital unit
    f. Current or last month treatment with hydroxychloroquine.

4. **WORKING GROUP**

- Dra. Carmen Margarita Hernández Cárdenas - Critical Care Department Chief
- Dr. Luis Felipe Jurado Camacho – Critical Care Unit Physician
- Dr. José Rogelio Pérez Padilla – COPD and Tobacco Research Department Chief
- Dra. Ireri Isadora Thirión Romero - COPD and Tobacco Research Department
- Dr. Sebastián Rodríguez Llamazares - COPD and Tobacco Research Department
- Dr. Joel Armando Vasquez Pérez- COPD and Tobacco Research Department
- Dra. Alejandra Ramirez Venegas - COPD and Tobacco Research Department

5. **BENEFITS AND RESULTS.**

Currently, there is a lack of a vaccine for SARSCoVid-19 and effective treatment, so if a utility of hydroxychloroquine treatment is demonstrated, in hospitalized patients, there would be an accessible, cheap medicine for patients with severe lung damage. The treatment would be widely applicable and highly useful mainly for developing countries.

6. **EXPECTED DELIVERABLES**

Specific Objective	Activities	Deliverables
Compare outcomes in control and experimental groups.	Clinical Trial	If it turns out beneficial, a safe accesible and cheap drug for COVID19 chemoprophylaxis will now be available.
Manuscript publication	Result report	Scientific publication
Scientific Congress Presentation	Result discusión	Scientific congress presentation

7. **EXECUTION CALENDAR**

Period in months

	1	2	3	4	5	6	7	8	9	10	11	12	13	14	15	16	17	18	19	20	21	22	23	24
Execution	x	x	x	x	x	x	x	x																
Analysis								x	x	x														
Publication preparation											x	X												

8. **REQUESTED FINANCING**

The recruited patient will not be charged for clinical care even if he/she requires intensive care. In addition to the costs of care, the project requests that the viral tests be repeated once a week, which is what has been proposed in any case for hospitalized patients, to be discharged preferably with the negative test. In addition to healthcare and testing costs, the primary requirement would be the purchase of the drug and the identical placebo, and the hiring of personnel to coordinate the trial, monitors.

9. **REFERENCES**

1. Chan JF, Yuan S, Kok KH, To KK, Chu H, Yang J, Xing F, Liu J, Yip CC, Poon RW, Tsoi HW, Lo SK, Chan KH, Poon VK, Chan WM, Ip JD, Cai JP, Cheng VC, Chen H, Hui CK, Yuen KY. A familial cluster of pneumonia associated with the 2019 novel coronavirus indicating person-to-person transmission: a study of a family cluster. *Lancet* 2020.
2. Chen N, Zhou M, Dong X, Qu J, Gong F, Han Y, Qiu Y, Wang J, Liu Y, Wei Y, Xia J, Yu T, Zhang X, Zhang L. Epidemiological and clinical characteristics of 99 cases of 2019 novel coronavirus pneumonia in Wuhan, China: a descriptive study. *Lancet* 2020.
3. Holshue ML, DeBolt C, Lindquist S, Lofy KH, Wiesman J, Bruce H, Spitters C, Ericson K, Wilkerson S, Tural A, Diaz G, Cohn A, Fox L, Patel A, Gerber SI, Kim L, Tong S, Lu X, Lindstrom S, Pallansch MA, Weldon WC, Biggs HM, Uyeki TM, Pillai SK, Washington State -nCo VCIT. First Case of 2019 Novel Coronavirus in the United States. *The New England journal of medicine* 2020.
4. Zhou P, Yang XL, Wang XG, Hu B, Zhang L, Zhang W, Si HR, Zhu Y, Li B, Huang CL, Chen HD, Chen J, Luo Y, Guo H, Jiang RD, Liu MQ, Chen Y, Shen XR, Wang X, Zheng XS, Zhao K, Chen QJ, Deng F, Liu LL, Yan B, Zhan FX, Wang YY, Xiao GF, Shi ZL. A pneumonia outbreak associated with a new coronavirus of probable bat origin. *Nature* 2020.
5. Zhu N, Zhang D, Wang W, Li X, Yang B, Song J, Zhao X, Huang B, Shi W, Lu R, Niu P, Zhan F, Ma X, Wang D, Xu W, Wu G, Gao GF, Tan W, China Novel Coronavirus I, Research T. A Novel Coronavirus from Patients with Pneumonia in China, 2019. *The New England journal of medicine* 2020.
6. Centers of Disease Control. 2019 Novel Coronavirus. 2020 [cited 2020 February 7]; Available from: https://http://www.cdc.gov/coronavirus/2019-ncov/locations-confirmed-cases.html
7. Zhao S, Musa SS, Lin Q, Ran J, Yang G, Wang W, Lou Y, Yang L, Gao D, He D, Wang MH. Estimating the Unreported Number of Novel Coronavirus (2019-nCoV) Cases in China in the First Half of January 2020: A Data-Driven Modelling Analysis of the Early Outbreak. *Journal of clinical medicine* 2020: 9(2).
8. Wang M, Cao R, Zhang L, Yang X, Liu J, Xu M, Shi Z, Hu Z, Zhong W, Xiao G. Remdesivir and chloroquine effectively inhibit the recently emerged novel coronavirus (2019-nCoV) in vitro. *Cell research* 2020.
9. Savarino A. Use of chloroquine in viral diseases. *The Lancet Infectious diseases* 2011: 11(9): 653-654.
10. Savarino A, Boelaert JR, Cassone A, Majori G, Cauda R. Effects of chloroquine on viral infections: an old drug against today's diseases? *The Lancet Infectious diseases* 2003: 3(11): 722-727.
11. Yan Y, Zou Z, Sun Y, Li X, Xu KF, Wei Y, Jin N, Jiang C. Anti-malaria drug chloroquine is highly effective in treating avian influenza A H5N1 virus infection in an animal model. *Cell research* 2013: 23(2): 300-302.
12. Gao J, Tian Z, Yang X. Breakthrough: Chloroquine phosphate has shown apparent efficacy in treatment of COVID-19 associated pneumonia in clinical studies. *Bioscience trends* 2020.
