# Supplementary material for: Hydroxychloroquine for the treatment of severe respiratory infection by COVID-19: A randomized controlled trial
Source: PLoS One. 2021 Sep 28;16(9):e0257238. doi: 10.1371/journal.pone.0257238 (PMC8478184; doi:10.1371/journal.pone.0257238)
Supplement: S1 Flow chart — (DOCX) [file pone.0257238.s009.docx]

Supplementary Figure1.

Assessed for eligibility (n=567)

Excluded (n=353)

♦  Not meeting inclusion criteria (n=211)

♦  Declined to participate (n=131)

♦  Other reasons: (n=11) participated in another clinical trial

Analysed (n= 106) None excluded.

None lost to follow up.

Treating physician discontinued intervention (n= 8)

Discontinued intervention because of adverse reactions (n= 4)

**Allocated to hydroxychloroquine (n=106)**

♦ Received allocated intervention (n=102)

♦ Did not receive allocated intervention (n=4)

n=1, randomized after acceptance with clinical and radiologic presentation consistent with COVID-19, but with PCR delayed, then the two firsts RT-PCR were negative and did not receive treatment.

n=3, randomized but the drug was lost in the ward and did not receive treatment.

None lost to follow up.

Treating physician discontinued intervention (n= 12)

Discontinued intervention because of adverse reactions (n= 4)

**Allocated to placebo (n=108)**

♦ Received allocated intervention (n=105)

Did not receive allocated intervention (n=3)

n=1, randomized after acceptance with clinical and radiologic presentation consistent with COVID-19, but with PCR delayed, then the two firsts RT-PCR were negative and did not receive treatment.

n=1, randomized but the drug was lost and did not receive treatment.

n=1, randomized but participated in another clinical trial

Analysed (n=108). None excluded.

## Analysis

## Follow-Up

Randomized (n= 214)

## Allocation

Flow Chart.
