# Supplementary material for: Hydroxychloroquine for the treatment of severe respiratory infection by COVID-19: A randomized controlled trial
Source: PLoS One. 2021 Sep 28;16(9):e0257238. doi: 10.1371/journal.pone.0257238 (PMC8478184; doi:10.1371/journal.pone.0257238)
Supplement: S1 Checklist — (DOC) [file pone.0257238.s010.doc]

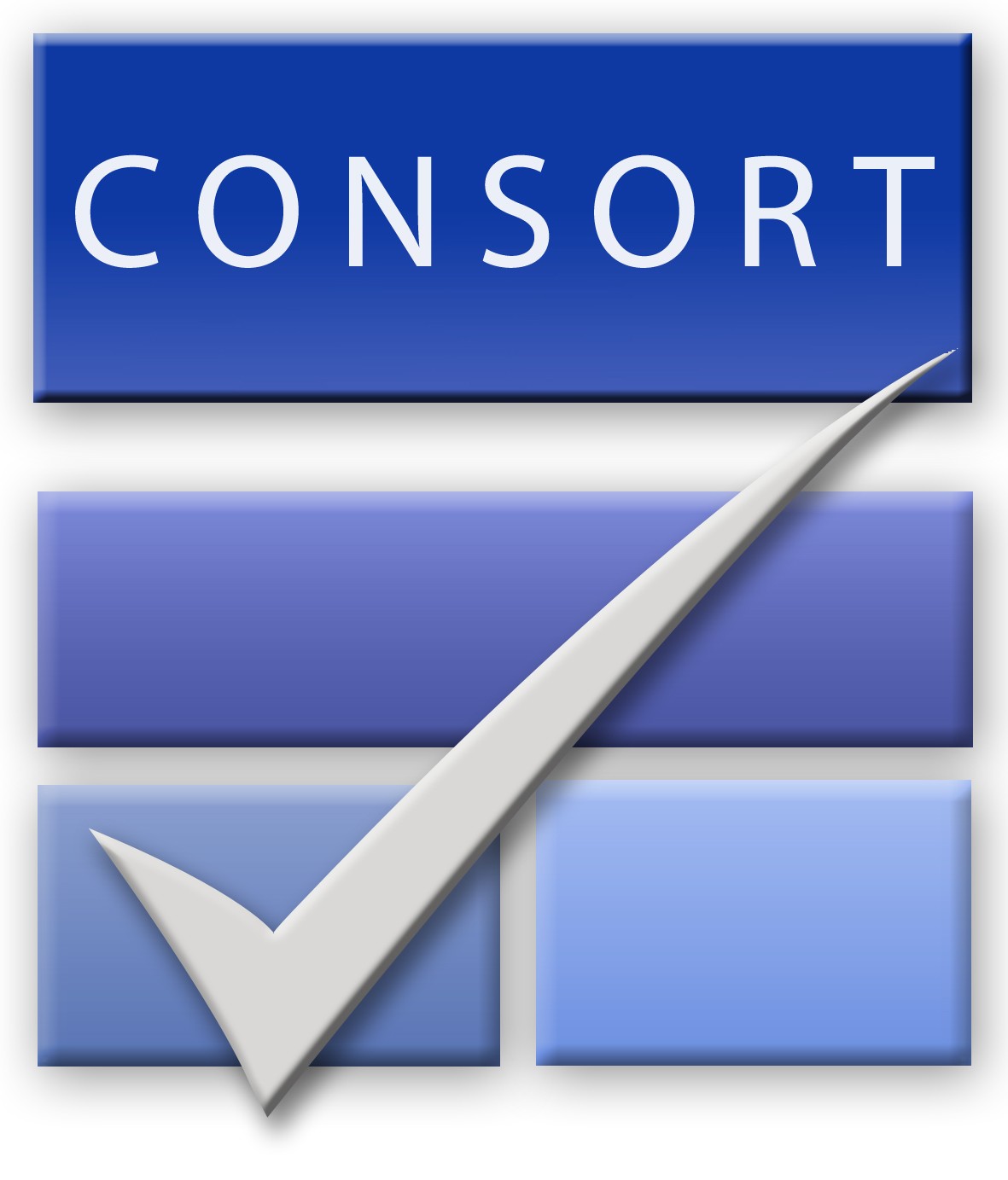
CONSORT 2010 checklist of information to include when reporting a randomised trial*

| Section/Topic | Item No | Checklist item | Reported on page No |
| --- | --- | --- | --- |
| Title and abstract | | | |
|  | 1a | Identification as a randomised trial in the title | Page 1, line 3 |
|  | 1b | Structured summary of trial design, methods, results, and conclusions (for specific guidance see CONSORT for abstracts) | Page 2, line 24 |
| Introduction | | | |
| Background and objectives | 2a | Scientific background and explanation of rationale | Page 3, line 47 |
|  | 2b | Specific objectives or hypotheses | Page 3, line 63 |
| Methods | | | |
| Trial design | 3a | Description of trial design (such as parallel, factorial) including allocation ratio | Page 3-4, line 67 |
|  | 3b | Important changes to methods after trial commencement (such as eligibility criteria), with reasons | Page 4, line 70 |
| Participants | 4a | Eligibility criteria for participants | Page 4, line 85 |
|  | 4b | Settings and locations where the data were collected | Page 5, line 106 |
| Interventions | 5 | The interventions for each group with sufficient details to allow replication, including how and when they were actually administered | Page 6, line 117 |
| Outcomes | 6a | Completely defined pre-specified primary and secondary outcome measures, including how and when they were assessed | Page 6, line 126 |
|  | 6b | Any changes to trial outcomes after the trial commenced, with reasons | Page 6, line 134 |
| Sample size | 7a | How sample size was determined | Page 6, line 131 |
|  | 7b | When applicable, explanation of any interim analyses and stopping guidelines | Page 6, line 134 |
| Randomisation: |  |  |  |
| Sequence generation | 8a | Method used to generate the random allocation sequence | Page 7, line 141 |
|  | 8b | Type of randomisation; details of any restriction (such as blocking and block size) | Page 7, line 141 |
| Allocation concealment mechanism | 9 | Mechanism used to implement the random allocation sequence (such as sequentially numbered containers), describing any steps taken to conceal the sequence until interventions were assigned | Page 7, line 141 |
| Implementation | 10 | Who generated the random allocation sequence, who enrolled participants, and who assigned participants to interventions | Page 7, line 145 |
| Blinding | 11a | If done, who was blinded after assignment to interventions (for example, participants, care providers, those assessing outcomes) and how | Page 8, line 160 |
|  | 11b | If relevant, description of the similarity of interventions | NA |
| Statistical methods | 12a | Statistical methods used to compare groups for primary and secondary outcomes | Page 8, line 164 |
|  | 12b | Methods for additional analyses, such as subgroup analyses and adjusted analyses | Page 8, line 166 |
| Results | | | |
| Participant flow (a diagram is strongly recommended) | 13a | For each group, the numbers of participants who were randomly assigned, received intended treatment, and were analysed for the primary outcome | Page 8, line 179 |
|  | 13b | For each group, losses and exclusions after randomisation, together with reasons | Page 8-9, line 181 |
| Recruitment | 14a | Dates defining the periods of recruitment and follow-up | Page 8, line 179 |
|  | 14b | Why the trial ended or was stopped |  |
| Baseline data | 15 | A table showing baseline demographic and clinical characteristics for each group | Page 9, line 198 |
| Numbers analysed | 16 | For each group, number of participants (denominator) included in each analysis and whether the analysis was by original assigned groups | Page 12, line 217 |
| Outcomes and estimation | 17a | For each primary and secondary outcome, results for each group, and the estimated effect size and its precision (such as 95% confidence interval) | Page 14, line 246 |
|  | 17b | For binary outcomes, presentation of both absolute and relative effect sizes is recommended | Page 17, line 276 |
| Ancillary analyses | 18 | Results of any other analyses performed, including subgroup analyses and adjusted analyses, distinguishing pre-specified from exploratory | Page 17, line 297 |
| Harms | 19 | All important harms or unintended effects in each group (for specific guidance see CONSORT for harms) | Page 18, line 285 |
| Discussion | | | |
| Limitations | 20 | Trial limitations, addressing sources of potential bias, imprecision, and, if relevant, multiplicity of analyses | Page 21, line 363 |
| Generalisability | 21 | Generalisability (external validity, applicability) of the trial findings | Page 21, line 371 |
| Interpretation | 22 | Interpretation consistent with results, balancing benefits and harms, and considering other relevant evidence | Page 21, line 371 |
| Other information | | |  |
| Registration | 23 | Registration number and name of trial registry | Page 4, line 81. |
| Protocol | 24 | Where the full trial protocol can be accessed, if available | Page 4, Line 71 |
| Funding | 25 | Sources of funding and other support (such as supply of drugs), role of funders | Page 24, line 417 |

*We strongly recommend reading this statement in conjunction with the CONSORT 2010 Explanation and Elaboration for important clarifications on all the items. If relevant, we also recommend reading CONSORT extensions for cluster randomised trials, non-inferiority and equivalence trials, non-pharmacological treatments, herbal interventions, and pragmatic trials. Additional extensions are forthcoming: for those and for up to date references relevant to this checklist, see [www.consort-statement.org](http://www.consort-statement.org/).
